# Morpho-phylogenetic evidence reveals novel hyphomycetous fungi on medicinal plants in Southwestern China

**DOI:** 10.1080/21501203.2024.2444436

**Published:** 2025-01-20

**Authors:** Hong-Zhi Du, Ning-Guo Liu, Na Wu, Ratchadawan Cheewangkoon, Jian-Kui Liu

**Affiliations:** a School of Life Science and Technology, Center for Informational Biology, University of Electronic Science and Technology of China, Chengdu, China; b School of Pharmacy, Guizhou University of Traditional Chinese Medicine, Guiyang, China; c Department of Entomology and Plant Pathology, Faculty of Agriculture, Chiang Mai University, Chiang Mai, Thailand; d Innovative Agriculture Research Centre, Faculty of Agriculture, Chiang Mai University, Chiang Mai, Thailand; e School of Food and Pharmaceutical Engineering, Guizhou Institute of Technology, Guiyang, China; f Center of Excellence in Fungal Research, Mae Fah Luang University, Chiang Rai, Thailand

**Keywords:** 12 new species, asexual morph, Dothideomycetes, phylogeny, Sordariomycetes, taxonomy

## Abstract

During a survey of saprobic fungi on medicinal plants in Southwestern China, thirty-nine hyphomycetous collections belonging to Dictyosporiaceae, Melanommataceae, and Stachybotryaceae were identified, representing nineteen distinct species. These taxa were characterised and identified based on morphological and culture characteristics, coupled with phylogenetic analyses of combined sequences of calmodulin (*cmdA*), the internal transcribed spacer region of ribosomal DNA (ITS), nuclear large subunit ribosomal DNA (LSU), RNA polymerase second-largest subunit (*rpb2*), the translation elongation factor 1-alpha (*tef1-α*), and β-tubulin (*tub2*). Twelve novel species are described, including *Camposporium alangii*, *C. polygoni*, *Dendryphiella verrucosispora*, *Jalapriya cheirospora*, *Memnoniella cnidiicola*, *M. guttulatispora*, *M. reniformis*, *M. reynoutriae*, *M. verrucosispora*, *Sirastachys aspidistrae*, *Sir. ellipsoidispora*, and *Striatibotrys biguttulatispora*. Additionally, three new host records, *M. alishanensis*, *Sir. castanedae*, and *Stachybotrys chartarum*, and four new hosts and geographical records of *M. ellipsoidea*, *M. pseudonilagirica*, *Str. rhabdospora*, and *Virgatospora echinofibrosa* are reported. *Memnoniella nilagirica* is revised and synonymised under *M. pseudonilagirica*. Key morphological characteristics, hosts specificity, and distributional data of *Memnoniella*, *Sirastachys*, and *Striatibotrys* were summarised. This study provides comprehensive illustrations, descriptions, and notes for each new taxon and record, marking the first report of these species from medicinal plants.

## 1. Introduction

Medicinal plants are rich resources of biological ingredients, which play a vital role in preventing and controlling diseases in human life (Nalawade et al. 2003; Cole et al. 2007; Samy and Gopalakrishnakone 2007; Sofowora et al. 2013; Schmidt 2017; Rahman et al. 2019; Ali et al. 2021; Atanasov et al. 2021). Over 70% of the global population relies on medicinal plants, with approximately 25% of modern drugs and up to 60% of antitumor drugs being derived from natural products (Brower 2008; Newman and Cragg 2012; David et al. 2014). The demand for medicinal plants has been rapidly growing worldwide (Hassan 2012; Rasool et al. 2020). The quality of medicinal plants, influenced by various factors including fungal pathogens, is crucial for clinical efficacy (Abtahi and Nourani 2017). Some endophytes can produce biologically active secondary metabolites (Helaly et al. 2018; Chen et al. 2021; Du et al. 2022), affecting the growth and quality of medicinal plants. Thus, the relationships between medicinal plants and microfungi remain a focal point of research (Weber et al. 2005; Sun et al. 2008; Abtahi and Nourani 2017; Keshri et al. 2021).

Melanommataceae G. Winter, established by Winter (1885) with *Melanomma* Nitschke ex Fuckel as the type genus, is distinguished by globose or depressed ascomata, trabeculate pseudoparaphyses, bitunicate and fissitunicate asci, and pigmented, phragmosporous ascospores (Hyde et al. 2013; Tian et al. 2015; Hongsanan et al. 2020). As one of the species-rich families within Pleosporales Luttr. ex M.E. Barr, Melanommataceae encompasses primarily saprobic and hyperparasitoidal fungi that typically inhabit the twigs and bark of woody plants across terrestrial, marine, and freshwater ecosystems (Tian et al. 2015; Hongsanan et al. 2020; Tennakoon et al. 2024). Currently, 36 genera are recognised within this family (Hongsanan et al. 2020; Gao et al. 2023; Tennakoon et al. 2024). Dictyosporiaceae Boonmee & K.D. Hyde (Pleosporales) was formally introduced by Boonmee et al. (2016) with *Dictyosporium* Corda designated as the type genus. This family was established to include species characterised by cheiroid, digitate, or dictyosporous, pale brown to brown conidia, and currently comprises 21 genera (Shen et al. 2022; Tian et al. 2022; Hyde et al. 2024). The majority of Dictyosporiaceae asexual morphs are hyphomycetous (Kirschner et al. 2013; Boonmee et al. 2016; Li et al. 2017; Liu et al. 2017, 2024; Iturrieta-González et al. 2018; Fu et al. 2021), with six genera (*Immotthia* M.E. Barr, *Pseudocoleophoma* Kaz. Tanaka & K. Hiray., *Pseudoconiothyrium* Crous & R.K. Schumach., *Pseudocyclothyriella* Phukhams. & Phookamsak, *Sajamaea* Flakus, Piatek & Rodr. Flakus, and *Verrucoccum* V. Atienza, D. Hawksw. & Pérez-Ort.) exhibiting coelomycetous asexual morphs (Tanaka et al. 2015; Piątek et al. 2020; Atienza et al. 2021; Jiang et al. 2021). The conidiogenous cells of *Dendryphiella* Bubák & Ranoj. are tretic, while other asexual morphs within the family show blastic or phialidic development (Boonmee et al. 2016; Liu et al. 2017; Piątek et al. 2020; Atienza et al. 2021; Jiang et al. 2021; Shen et al. 2022). Sexual morphs within Dictyosporiaceae are typified by semi-immersed to immersed, subglobose to globose, brown to black ascomata, with bitunicate, cylindrical to clavate asci containing septate, hyaline, sheathed ascospores, as seen in *Dictyosporium*, *Gregarithecium* Kaz. Tanaka & K. Hiray., *Immotthia*, *Pseudocoleophoma*, and *Verrucoccum* (Barr 1987; Tanaka et al. 2015; Jayasiri et al. 2019; Atienza et al. 2021; Lu et al. 2022).

Stachybotryaceae L. Lombard & Crous (Hypocreales) was introduced by Crous et al. (2014), encompassing 41 genera (Hyde et al. 2020b, 2024). Species of Stachybotryaceae have sporodochial or synnematal conidiomata, phialidic conidiogenous cells, and produce conidia in chains or aggregated in slimy masses (Seifert and Gams 2011; Crous et al. 2014; Wang et al. 2015; Lin et al. 2016, 2017; Lombard et al. 2016; Hyde et al. 2017). Lombard et al. (2016) expanded the phylogenetic framework of Stachybotryaceae through multi-locus analyses (ITS, LSU, *cmdA*, *rpb2*, *tef1-α*, and *tub2*). Subsequently, numerous novel species of Stachybotryaceae have been described based on morphology and phylogenetic evidence (Lechat et al. 2017; Crous et al. 2018; Zheng et al. 2019; Hyde et al. 2020b; Samarakoon et al. 2021; Tennakoon et al. 2021; Tian et al. 2021; Yeh et al. 2023). Members of Stachybotryaceae inhabit diverse environments (soil, marine, freshwater, air, etc.) with various life modes including saprobes (Li and Jiang 2011; Samarakoon et al. 2021; Tennakoon et al. 2021), plant pathogens (Pathak and Chauhan 1976; Zhao et al. 2010), human pathogens (Mason et al. 1998; Hossain et al. 2004) and endophytes (Cao et al. 2009; Raghavendra and Newcombe 2012; Busby et al. 2016; Zhang et al. 2019; Yang et al. 2020). Additionally, a few *Stachybotrys* Corda species, such as *S. chartarum* (Ehrenb.) S. Hughes, *S. elegans* (Pidopl.) W. Gams, and *S. microsporus* (B.L. Mathur & Sankhla) S.C. Jong & E.E. Davis produce mycotoxins with veterinary and medical significance (Jarvis et al. 1995; Mason et al. 1998; Hossain et al. 2004; Lichtenstein et al. 2010; Hyde et al. 2019).

Our recent collections encompass eight genera, *Camposporium* Harkn. (Melanommataceae); *Dendryphiella* and *Jalapriya* D’souza, H.Y. Su, Z. Luo & K.D. Hyde (Dictyosporiaceae); and *Memnoniella* Höhn., *Sirastachys* L. Lombard & Crous, *Stachybotrys*, *Striatibotrys* L. Lombard & Crous, and *Virgatospora* Finley (Stachybotryaceae). An increasing number of new species and records for these genera have been reported from various hosts, with most species identified as saprotrophic fungi from a range of habitats and ecological niches (Lin et al. 2016; Doilom et al. 2017; Liu et al. 2017; Hyde et al. 2020a, 2020b; Mapook et al. 2020; Calabon et al. 2021; Fu et al. 2021; Tennakoon et al. 2021, 2023; Xu et al. 2021; Tian et al. 2024). However, reports of these genera specifically on medicinal plants remain limited. Given the significantly economic, ecological, and medicinal value of these hosts, it is essential to investigate the taxonomy and phylogeny of microfungi associated with medicinal flora to expand our understanding of fungal diversity within these specialised niches. Additionally, because the quality of medicinal plants is closely tied to their clinical efficacy, this study aims to establish a foundation for exploring potential interactions between medicinal plant quality and associated microfungi, thus contributing valuable insights for the quality assurance of Chinese herbal medicines.

During the investigation of hyphomycetous fungi from medicinal plants in Southwestern China, this study explores the diversity within the families Dictyosporiaceae, Melanommataceae, and Stachybotryaceae, documenting 19 hyphomycetous species. Morphological analyses, integrated with multi-locus phylogenetic assessments, were conducted to refine the classification of these collections. This investigation introduces 12 novel species, three new host records, and four new host and geographic records, all detailed herein.

## 2. Materials and methods

### 2.1. Collection and examination of specimens

**Figure 1.**
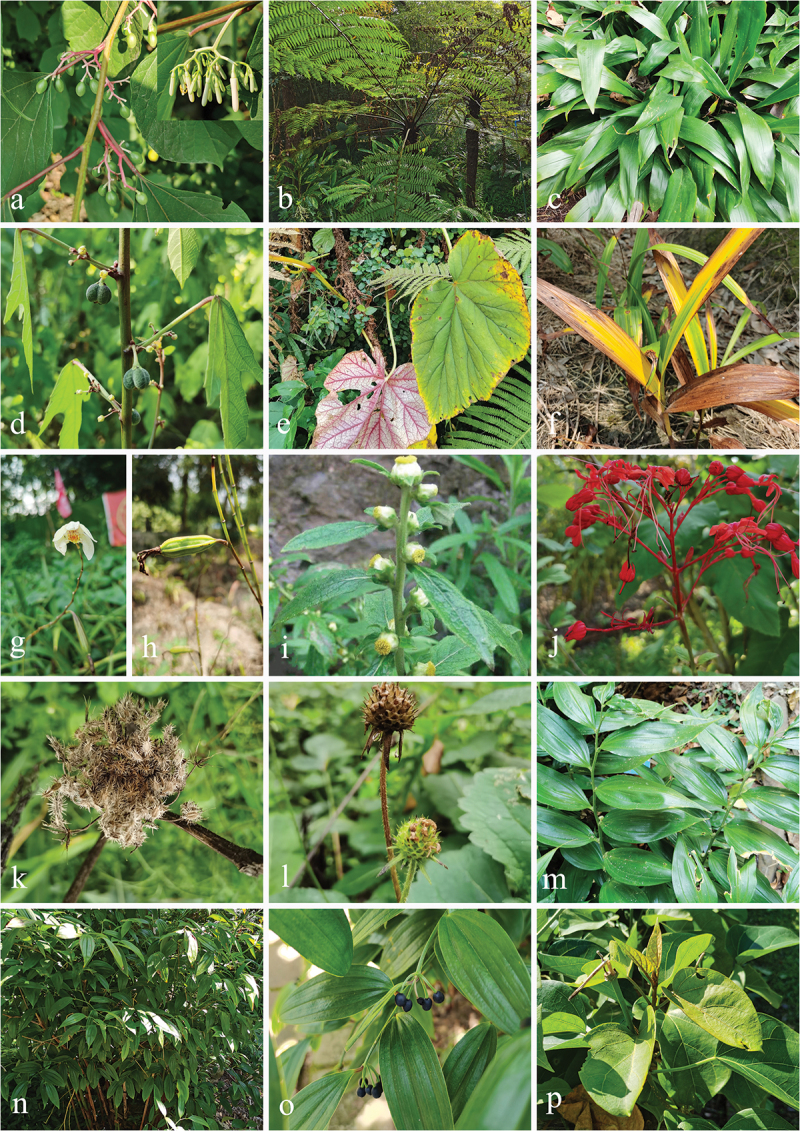
The photos of medicinal plant hosts (I). (a) *Alangium chinense* (Lour.) harms (Cornaceae). (b) *Alsophila spinulosa* (Wall. ex Hook.) R. M. Tryon (Cyatheaceae). (c) *Aspidistra elatior* Blume (Asparagaceae). (d) *Baliospermum solanifolium* (Burm.) Suresh (Euphorbiaceae). (e) *Begonia grandis* Dryand. (Begoniaceae). (f–h) *Bletilla striata* (Thunb. ex A. Murray) Rchb. f. (Orchidaceae). (i) *Carpesium abrotanoides* L. (Asteraceae). (j) *Clerodendrum japonicum* (Thunb.) Sweet (Lamiaceae). (k) *Cnidium monnieri* (L.) Spreng. (Apiaceae). (l) *Dipsacus asper* Wall. (Dipsacaceae). (m) *Disporopsis longifolia* Craib (Asparagaceae). (n, o) *Disporum Longistylum* (H. Lév. & Vaniot) H. Hara (Liliaceae). (p) *Dregea volubilis* (L. f.) Benth. ex Hook. f. (Apocynaceae).

**Figure 2.**
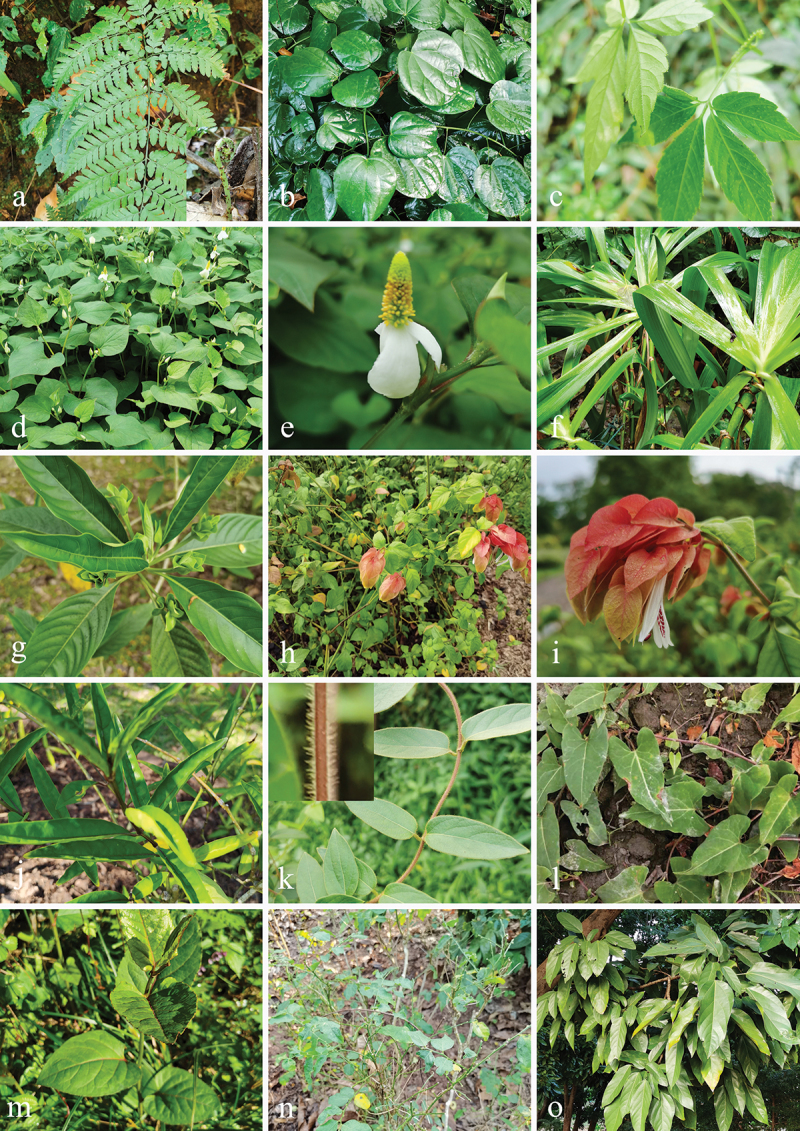
The photos of medicinal plant hosts (II). (a) *Dryopteris* sp. (Dryopteridaceae). (b) *Fibraurea recisa* Pierre (Menispermaceae). (c) *Gynostemma pentaphyllum* (Thunb.) Makino (Cucurbitaceae). (d, e) *Houttuynia cordata* Thunb. (Saururaceae). (f) *Iris wattii* Baker (Iridaceae). (g) *Justicia adhatoda* L. (Acanthaceae). (h, i) *Justicia brandegeeana* Wassh. & L. B. Sm. (Acanthaceae). (j) *Justicia gendarussa* Burm. f. (Acanthaceae). (k) *Lonicera guillonii* var. *macranthoides* (Hand.-Mazz.) Z.H. Chen & X.F. Jin (Caprifoliaceae). (l) *Polygonum multiflorum* Thunb. (Polygonaceae). (m) *Reynoutria japonica* Houtt. (Polygonaceae). (n) *Sauropus androgynus* auct. non (L.) Merr. (Euphorbiaceae). (o) *Scaphium wallichii* (Wall. ex G. Don) Schott & Endl. (Malvaceae).

Specimens were collected from 21 families of medicinal plants (Acanthaceae Juss., Apiaceae Lindl., Apocynaceae Juss., Asparagaceae Juss., Asteraceae Bercht. & J. Presl, Begoniaceae C. Agardh, Caprifoliaceae Juss., Cornaceae Bercht. & J. Presl, Cucurbitaceae Juss., Cyatheaceae Kaulf., Dipsacaceae Deyuan Hong, Liming Ma & Fred R. Barrie, Dryopteridaceae Hene, Euphorbiaceae Juss., Iridaceae Juss., Lamiaceae Martinov, Liliaceae Juss., Malvaceae Juss., Menispermaceae Juss., Orchidaceae Juss., Polygonaceae Juss., and Saururaceae Rich. ex T. Lestib.) in Southwestern China from December 2020 to November 2022, viz., Guizhou Province (106°38′13′′−106°41′48′′E, 26°22′14′′–26°32′18′′N, elevation 1,127–1,140 m); Sichuan Province (103°28′36′′–104°7′19′′E, 29°21′60′′–30°56′24′′N, elevation 504–1,189 m); Yunnan Province (100°47′17′′–102°44′24′′E, 21°55′34′′–25°8′27′′N, elevation 502–1,922 m), and the sampling information (date, host, place, GPS, et al.) was recorded. The plant species were identified based on morphological characteristics and verified using the Kew Gardens Database (https://mpns.science.kew.org/) for taxonomic validation (Figures 1 and 2).

Samples, including dead leaves, petioles, stems, twigs, and vines from medicinal plants, were collected and packaged in envelopes, and brought to the laboratory following the method described by Senanayake et al. (2020). Morphological examination of fungal structures on natural substrates was conducted using a Motic SMZ 168 Series stereoscopic zoom microscope (Motic, Xiamen, China). Fruiting bodies were collected with a syringe needle and transferred to a drop of tap water on a clean slide for observation. Features were observed and photographed using a Nikon ECLIPSE Ni-U compound microscope equipped with a Nikon DS-Ri2 digital camera. Measurement principles were carried out by the Tarosoft Image Frame Work v. 0.9.7 software following the procedures outlined by Liu et al. (2010). Photo plate images were processed in Adobe Photoshop CC 2018 (Adobe Systems, San Jose, CA, USA). Single spore isolations were made on potato dextrose agar (PDA, Oxoid) or water agar (WA, Oxoid) and transferred to fresh PDA plates, following the method described in Senanayake et al. (2020). The plates were incubated in an incubator under dark conditions at 25 °C, and pure cultures were obtained.

Herbarium specimens were deposited in the Herbarium of Cryptogams, Kunming Institute of Botany Academia Sinica (HKAS), Kunming, China, and Herbarium, University of Electronic Science and Technology (HUEST), Chengdu, China. The pure cultures were deposited in the China General Microbiological Culture Collection Center (CGMCC) in Beijing, China, and the University of Electronic Science and Technology Culture Collection (UESTCC), Chengdu, China. The new taxa were registered in MycoBank (https://www.mycobank.org/; accessed on 1 June 2024).

### 2.2. DNA extraction, PCR amplification and sequencing

Isolates grew in PDA medium in an incubator under dark conditions for three weeks to one month at 25 °C. Fungal mycelia were scraped off and transferred to 1.5 mL microcentrifuge tubes using a sterilised lancet for genomic DNA extraction. Fungal DNA was extracted from mycelia (about 50–100 mg) using the Trelief TM Plant Genomic DNA Kit (TsingKe Co., Beijing, China) following the manufacturer’s instructions. Six different gene regions were amplified by Polymerase Chain Reaction (PCR). The internal transcribed spacer region of ribosomal DNA (ITS), nuclear large subunit ribosomal DNA (LSU), RNA polymerase second-largest subunit (*rpb2*), calmodulin (*cmdA*), β-tubulin (*tub2*), and the translation elongation factor 1-alpha (*tef1-α*) loci were amplified via polymerase chain reaction (PCR) using the following primers, The amplifications were performed in a 25 μL reaction volume containing 9.5 μL ddH_2_O, 12.5 μL 2× Taq PCR Master Mix with blue dye (Sangon Biotech, Shanghai, China), 1 μL of DNA template and 1 μL of each primer. The PCR product purification was performed using gel extraction technique, followed by Sanger sequencing at Beijing Tsingke Biotechnology (Chengdu) Co., Ltd. in China. The loci, primers, and amplification conditions are listed in Table 1.

**Table 1.** Loci, primers, and amplification conditions in this study.

Locus	Primers	Optimised PCR protocols	References
ITS	ITS5	94 °C 3 min; 35 cycles of 94 °C 45 s, 55 °C 55 s, 72 °C 1 min; 10 min at 72 °C; 4 °C on hold	White et al. (1990)
	ITS4		
LSU	LR0R	94 °C 3 min; 36 cycles of 94 °C 45 s, 55 °C 55 s, 72 °C 1 min; 10 min at 72 °C; 4 °C on hold	Vilgalys and Hester (1990)
	LR5		
*rpb2*	fRPB2-5F	95 °C 5 min; 40 cycles of 95 °C 15 s, 56 °C 50 s, 72 °C 2 min; 10 min at 72 °C; 4 °C on hold	O’Donnell et al. (2007)
	fRPB2-7cR		
*cmdA*	CAL-228F	95 °C 5 min; 35 cycles of 95 °C 15 s, 55 °C 20 s, 72 °C 40 s; 5 min at 72 °C; 4 °C on hold	Carbone and Kohn (1999)
	CAL2Rd		Groenewald et al. (2013)
*tub2*	Bt2a	94 °C 3 min; 35 cycles of 94 °C 45 s, 55 °C 55 s, 72 °C 1 min; 10 min at 72 °C; 4 °C on hold	Glass and Donaldson (1995)
	Bt2b		
*tef1-α*	TEF1-983F	94 °C 3 min; 38 cycles of 94 °C 45 s, 56 °C 55 s, 72 °C 1 min; 10 min at 72 °C; 4 °C on hold	Rehner and Buckley (2005)
	TEF1-2218R		
*tef1-α*	EF1-728F	94 °C 3 min; 35 cycles of 94 °C 45 s, 55 °C 55 s, 72 °C 1 min; 10 min at 72 °C; 4 °C on hold	Carbone and Kohn (1999)
	EF2		O’Donnell et al. (1998)

To better reveal the phylogenetic relationships and taxonomic distinctions of new fungal collections, multi-locus phylogenetic analyses were conducted based on the commonly used loci across different groups (Stielow et al. 2015). The loci and primers used for *Memnoniella*, *Sirastachys*, *Stachybotrys*, *Striatibotrys*, and *Virgatospora* are *cmdA* (CAL-228F/CAL2Rd), ITS (ITS5/ITS4), *rpb2* (fRPB2-5F/fRPB2-7cR), *tub2* (Bt2a/Bt2b), and *tef1-α* (EF1-728F/EF2). *Dendryphiella* and *Jalapriya* are ITS (ITS5/ITS4), LSU (LR0R/LR5), and *tef1-α* (TEF1-983F/TEF1-2218R). For *Camposporium*, ITS (ITS5/ITS4), *tef1-α* (TEF1-983F/TEF1-2218R), and *rpb2* (fRPB2-5F/fRPB2-7cR) were used.

### 2.3. Phylogenetic analyses

In this study, the taxa included in the phylogenetic analyses were selected and obtained from GenBank. Alignments for each locus were made in MAFFT v. 7 (http://mafft.cbrc.jp/alignment/server/) (Katoh and Standley 2013) and checked visually using AliView (Larsson 2014). The alignments were trimmed using trimAl v. 1.2 (Capella-Gutierrez et al. 2009) with minimal coverage (-cons) = 0.8 and gap threshold (-gt) = 0.6. Single locus alignments were combined using Sequence Matrix 1.7.8 (Vaidya et al. 2011). Maximum likelihood (ML) and Bayesian inference (BI) analyses were employed to assess phylogenetic relationships as detailed in Dissanayake et al. (2020).

ML analyses were performed with RAxML-RAxMLHPC2 v. 8 on XSEDE (8.2.12) (Stamatakis 2006; Stamatakis et al. 2008) through the CIPRES Science Gateway v. 3.3 (https://www.phylo.org/portal2/login!input.action) (Miller et al. 2010). The tree search included 1,000 non-parametric bootstrap replicates; the best scoring tree was selected among suboptimal trees from each run by comparing likelihood scores under the GTRGAMMA substitution model. The resulting replicates were plotted onto the best scoring tree obtained previously. ML bootstrap values equal to or greater than 75% were marked near each node.

BI was performed in MrBayes 3.2.6 (Ronquist et al. 2012). The program MrModeltest 2 v. 2.3 (Nylander 2008) was used to determine the best nucleotide substitution model for each data partition. Posterior probabilities (PP) (Rannala and Yang 1996) were determined by Markov chain Monte Carlo sampling (MCMC). Six simultaneous Markov chains were run for 10 million generations, and trees were sampled every 1,000th generation, and ending the run automatically when the standard deviation of split frequencies dropped below 0.01. The first 25% of saved trees, representing the burn-in phase of the analysis, were discarded. The remaining trees were used for calculating posterior probabilities in the majority rule consensus tree (Larget and Simon 1999). PP values equal to or greater than 0.95 were marked near each node.

Phylogenetic trees were printed with Fig Tree v. 1.4.4 (http://tree.bio.ed.ac.uk/software/figtree/) and the layout was created in Adobe Illustrator CS6 software (Adobe Systems, USA). The new sequences generated in this study were deposited in GenBank (Table 2).

**Table 2.** The newly generated sequences in this study and the corresponding GenBank accession number.

Species	Isolates number	GenBank accession number					
		*cmdA*	ITS	LSU	*rpb2*	*tef1-α*	*tub2*
*Camposporium alangii*	UESTCC 23.0509^T^	N/A	PQ415533	N/A	PQ432226	PQ432234	N/A
*Camposporium alangii*	UESTCC 24.0208	N/A	PQ415537	N/A	PQ432230	PQ432238	N/A
*Camposporium polygoni*	UESTCC 23.0506^T^	N/A	PQ415532	N/A	PQ432225	PQ432233	N/A
*Camposporium polygoni*	UESTCC 24.0207	N/A	PQ415536	N/A	PQ432229	PQ432237	N/A
*Dendryphiella verrucosispora*	UESTCC 23.0504^T^	N/A	PQ415535	PQ415541	PQ432228	PQ432236	N/A
*Dendryphiella verrucosispora*	UESTCC 24.0205	N/A	PQ415539	PQ415543	PQ432232	PQ432240	N/A
*Jalapriya cheirospora*	UESTCC 23.0505^T^	N/A	PQ415534	PQ415540	PQ432227	PQ432235	N/A
*Jalapriya cheirospora*	UESTCC 24.0206	N/A	PQ415538	PQ415542	PQ432231	PQ432239	N/A
*Memnoniella alishanensis*	UESTCC 23.0159	N/A	PP831946	N/A	PP855580	PP838852	PP838882
*Memnoniella alishanensis*	CGMCC 3.25611	N/A	PP831947	N/A	PP855581	PP838853	PP838883
*Memnoniella alishanensis*	CGMCC 3.25612	N/A	PP831948	N/A	PP855582	PP838854	PP838884
*Memnoniella alishanensis*	CGMCC 3.25613	N/A	PP831949	N/A	PP855583	PP838855	PP838885
*Memnoniella alishanensis*	UESTCC 23.0163	N/A	PP831950	N/A	PP855584	PP838856	PP838886
*Memnoniella cnidiicola*	CGMCC 3.25686^T^	PP838827	PP831951	N/A	PP855585	PP838857	PP838887
*Memnoniella cnidiicola*	UESTCC 23.0167	PP838828	PP831952	N/A	PP855586	PP838858	PP838888
*Memnoniella ellipsoidea*	CGMCC 3.25685	N/A	PP831953	N/A	N/A	N/A	PP838889
*Memnoniella guttulatispora*	UESTCC 23.0164^T^	PP838829	PP831954	N/A	PP855587	PP838859	PP838890
*Memnoniella pseudonilagirica*	UESTCC 23.0171	PP838830	PP831955	N/A	PP855588	PP838860	PP838891
*Memnoniella pseudonilagirica*	UESTCC 23.0172	PP838831	PP831956	N/A	PP855589	PP838861	PP838892
*Memnoniella pseudonilagirica*	CGMCC 3.25616	PP838832	PP831957	N/A	PP855590	PP838862	PP838893
*Memnoniella pseudonilagirica*	CGMCC 3.25617	PP838833	PP831958	N/A	PP855591	PP838863	PP838894
*Memnoniella reniformis*	UESTCC 23.0170^T^	PP838834	PP831959	N/A	PP855592	PP838864	PP838895
*Memnoniella reynoutriae*	CGMCC 3.25615^T^	PP838835	PP831960	N/A	PP855593	PP838865	PP838896
*Memnoniella reynoutriae*	UESTCC 23.0169	PP838836	PP831961	N/A	PP855594	PP838866	PP838897
*Memnoniella verrucosispora*	CGMCC 3.25614^T^	PP838837	PP831962	N/A	PP855595	PP838867	PP838898
*Memnoniella verrucosispora*	UESTCC 23.0168	PP838838	PP831963	N/A	PP855596	PP838868	PP838899
*Sirastachys aspidistrae*	CGMCC 3.25687^T^	PP838842	PP831967	N/A	PP855600	PP838872	PP838903
*Sirastachys aspidistrae*	UESTCC 23.0182	PP838843	PP831968	N/A	PP855601	PP838873	PP838904
*Sirastachys castanedae*	UESTCC 23.0184	PP838839	PP831964	N/A	PP855597	PP838869	PP838900
*Sirastachys ellipsoidispora*	CGMCC 3.25621^T^	PP838840	PP831965	N/A	PP855598	PP838870	PP838901
*Sirastachys ellipsoidispora*	UESTCC 23.0183	PP838841	PP831966	N/A	PP855599	PP838871	PP838902
*Stachybotrys chartarum*	UESTCC 23.0181	PP838844	PP831969	N/A	PP855602	PP838874	PP838905
*Striatibotrys biguttulatispora*	UESTCC 23.0175	PP838845	PP831970	N/A	PP855603	PP838875	PP838906
*Striatibotrys biguttulatispora*	UESTCC 23.0176	PP838846	PP831971	N/A	PP855604	PP838876	PP838907
*Striatibotrys biguttulatispora*	UESTCC 23.0177	PP838847	PP831972	N/A	PP855605	PP838877	PP838908
*Striatibotrys biguttulatispora*	CGMCC 3.25618	PP838848	PP831973	N/A	PP855606	PP838878	PP838909
*Striatibotrys biguttulatispora*	CGMCC 3.25619^T^	PP838849	PP831974	N/A	PP855607	PP838879	PP838910
*Striatibotrys rhabdospora*	CGMCC 3.25620	PP838850	PP831975	N/A	PP855608	PP838880	PP838911
*Virgatospora echinofibrosa*	UESTCC 23.0185	PP838851	PP831976	N/A	PP855609	PP838881	PP838912

^T^denotes ex-type isolates. “N/A” denotes sequence is unavailable.

## 3. Results

### 3.1. Phylogenetic results

In this study, three phylogenetic analyses were conducted to resolve the phylogenetic affinities of 12 novel species, three new host records, four new hosts and geographical records. The analyses focused on the following groups: Analysis 1 examined the genera *Dendryphiella* and *Jalapriya* (Dictyosporiaceae/Pleosporales/Dothideomycetes); Analysis 2 examined the genus *Camposporium* (Melanommataceae/Pleosporales/Dothideomycetes); and Analysis 3 examined the genera *Memnoniella*, *Sirastachys*, *Stachybotrys*, *Striatibotrys*, and *Virgatospora* (Stachybotryaceae/Hypocreales/Sordariomycetes).

**Figure 3.**
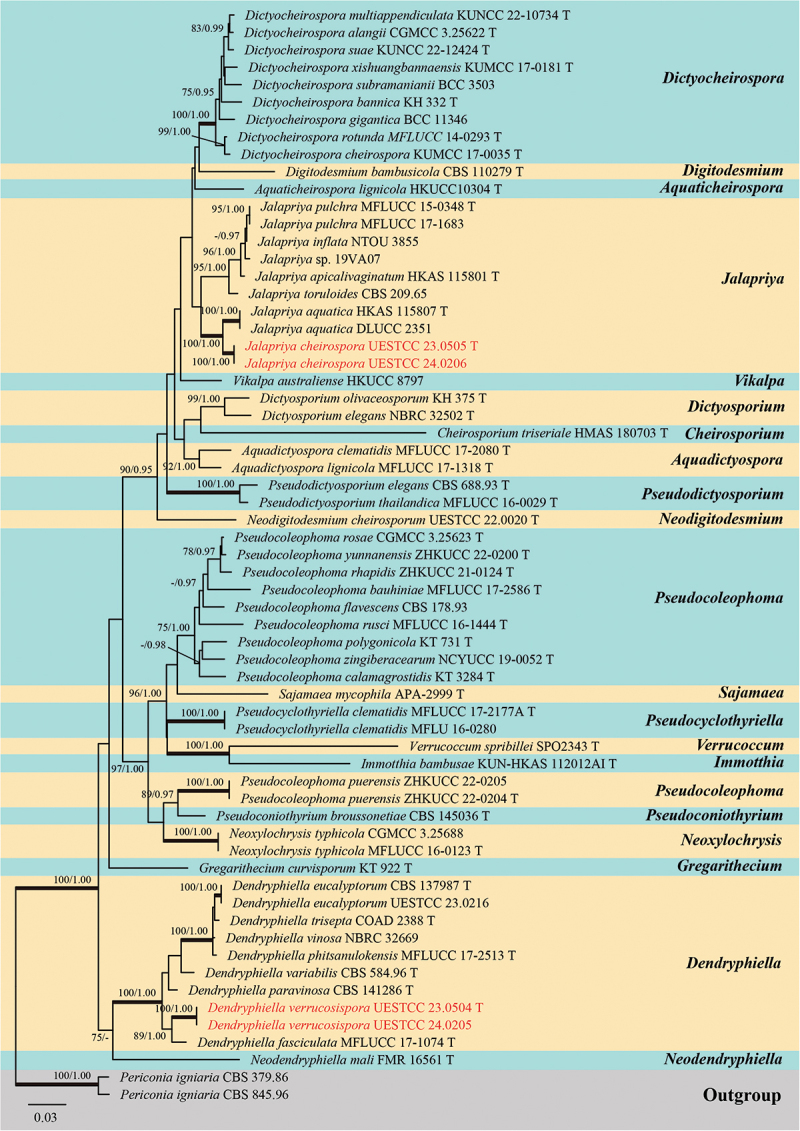
Phylogenetic tree constructed from maximum likelihood (RAxML) analyses of combined ITS, LSU, SSU, and *tef1-α* sequence data for selected genera within the family Dictyosporiaceae (Pleosporales). Branches support for maximum likelihood (MLBS) equal to or greater than 75% and Bayesian inference posterior probabilities (BIPP) equal to or greater than 0.95 are marked above or below branches as MLBS/BIPP. The abbreviation T indicates the ex-type isolates. Species’ names and culture collections in red are newly collected taxa. The tree was rooted with *Periconia igniaria* E.W. Mason & M.B. Ellis (CBS 379.86 and CBS 845.96).

Analysis 1: Four loci (ITS, LSU, SSU, and *tef1-α*) were used to determine the phylogenetic placements of the new species. The concatenated matrix is composed of 63 isolates with a total of 3,302 bp characters (ITS: 1–521 bp; LSU: 522–1,351 bp; SSU: 1,352–2,368 bp; *tef1-α*: 2,369–3,302 bp) after alignments, including gaps. Single locus analyses were carried out to compare the topologies and clade stabilities. The results showed that the different loci were similar in topology without significant conflicts. The best RAxML tree, with a final likelihood value of −19,258.437021, is presented in Figure 3. For the BI analysis, the final average standard deviation of split frequencies at the end of total MCMC generations was 0.009941, and the evolutionary model of GTR+I+G substitution model was selected for ITS, LSU, and *tef1-α* loci, and HKY+I+G substitution model was selected for SSU locus. The multi-locus phylogenetic trees showed similar topologies between the ML and BI analyses.

Multi-locus phylogenetic tree showed that our new species, *Dendryphiella verrucosispora* (UESTCC 23.0504 and UESTCC 24.0205), and *Jalapriya cheirospora* (UESTCC 23.0505 and UESTCC 24.0206), formed two distinct lineages and clustered within the genera *Dendryphiella* and *Jalapriya*, respectively (Figure 3).

**Figure 4.**
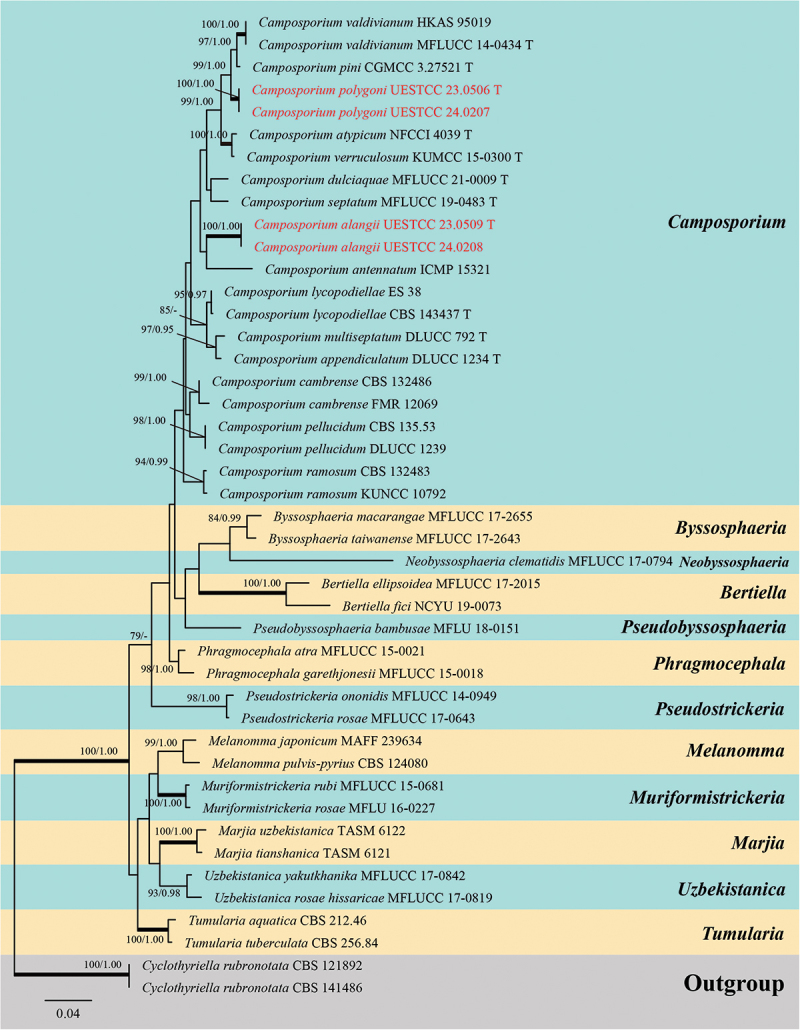
Phylogenetic tree constructed from maximum likelihood (RAxML) analyses of combined ITS, LSU, SSU, *tef1-α*, and *rpb2* sequence data. Branches support for maximum likelihood (MLBS) equal to or greater than 75% and Bayesian inference posterior probabilities (BIPP) equal to or greater than 0.95 are marked above or below branches as MLBS/BIPP. The abbreviation T indicates the ex-type isolates. Species’ names and culture collections in red are newly collected taxa. The tree was rooted with *Cyclothyriella rubronotata* (Berk. & Broome) Jaklitsch & Voglmayr (CBS 121892 and CBS 141486).

Analysis 2: Five loci (ITS, LSU, SSU, *tef1-α*, and *rpb2*) were used to determine the phylogenetic placements of the new species. The concatenated matrix comprised 44 isolates with a total of 4,354 bp characters (ITS: 1–517 bp; LSU: 518–1,359 bp; SSU: 1,360–2,376 bp; *tef1-α*: 2,377–3,310 bp; *rpb2*: 3,311–4,354 bp) after alignments, including gaps. Single locus analyses were carried out to compare the topologies and clade stabilities. The results showed that the different loci were similar in topology without significant conflicts. The best RAxML tree, with a final likelihood value of −18,250.937223, is presented in Figure 4. For the BI analysis, the final average standard deviation of split frequencies at the end of total MCMC generations was 0.009963, and the evolutionary model GTR+I+G substitution model was selected for LSU and *tef1-α* loci, and SYM+I+G, GTR+G, and SYM+G substitution model was selected for ITS, SSU, and *rpb2* loci, respectively. The multi-locus phylogenetic trees showed similar topologies between the ML and BI analyses.

Phylogenetic analyses showed that our new species clustered within the genus *Camposporium* (Figure 4). *Camposporium polygoni* (UESTCC 23.0506 and UESTCC 24.0207) formed an independent subclade and grouped with *C. valdivianum* (Speg.) G. Delgado & Koukol (MFLUCC 14-0434 and HKAS 95019) and *C. pini* W.H. Tian, K.D. Hyde & Maharachch. (CGMCC 3.27521) with high support (99% MLBS/1.00 BIPP). *Camposporium alangii* (UESTCC 23.0509 and UESTCC 24.0208) was phylogenetically independent species (100% MLBS/1.00 BIPP), and formed a sister clade to *C. antennatum* Harkn. (ICMP 15321) with low statistical support.

**Figure 5.**
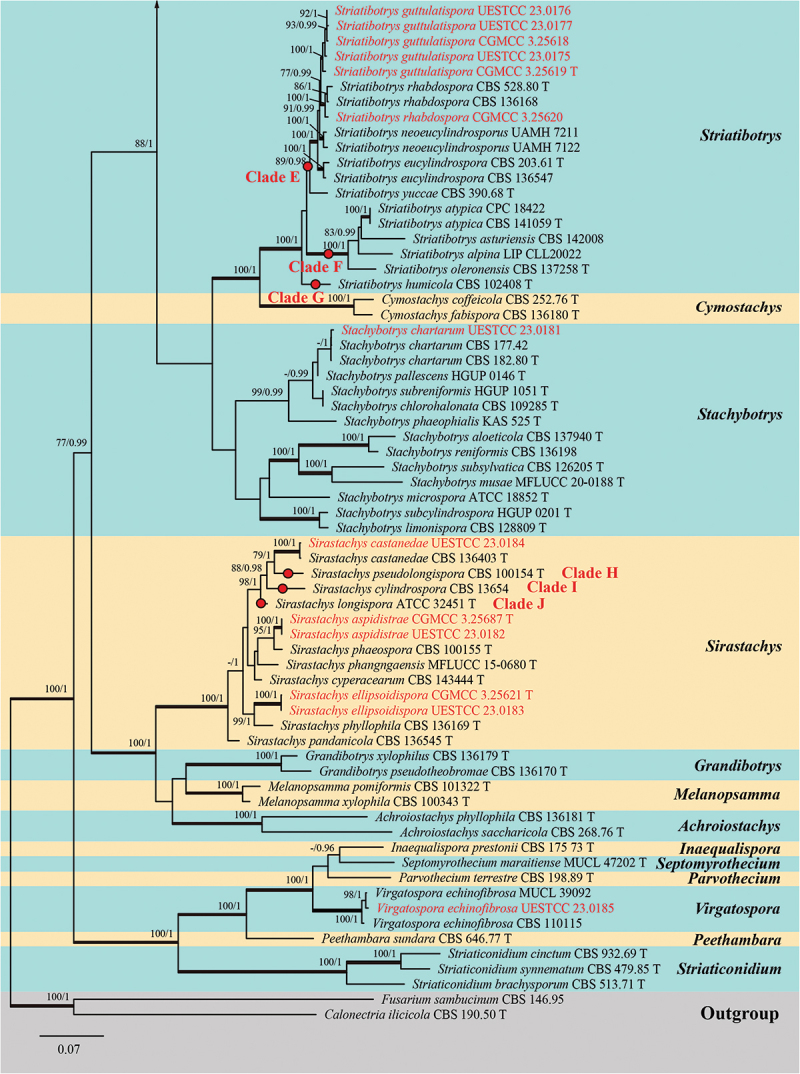
(Continued).

Analysis 3: Five loci, namely *cmdA*, ITS, *rpb2*, *tef1-α*, and *tub2*, were used to determine the phylogenetic placements of the new species. The concatenated matrix comprised 116 isolates with a total of 2,729 characters (*cmdA*: 1−644 bp, ITS: 645−1,212 bp, *rpb2*: 1,213−1,933 bp, *tef1-α*: 1,934−2,388 bp, *tub2*: 2,389−2,729 bp) after alignments, including gaps. Single locus analyses were carried out to compare the topologies and clade stabilities. The results showed that the different loci were similar in topology without significant conflicts. The best RAxML tree, with a final likelihood value of −46,959.888305, is presented in Figure 5. For the BI analysis, the final average standard deviation of split frequencies at the end of total MCMC generations was 0.009997 (the critical value for the topological convergence diagnostic is below 0.01), and the evolutionary model of GTR+I+G substitution model was selected for ITS, *rpb2*, and *tub2* loci, and HKY+I+G substitution model was selected for *cmdA* and *tef1-α* genes. The multi-locus phylogenetic trees showed that the ML and BI analyses were similar in topology without significant conflicts, and these results are consistent with previous studies (Lin et al. 2016; Lombard et al. 2016; Samarakoon et al. 2021; Tennakoon et al. 2021). As a result, thirty-one isolates obtained in this study were grouped into fifteen clades and classified into five genera, namely *Memnoniella* (eighteen isolates), *Striatibotrys* (six isolates), *Sirastachys* (five isolates), *Stachybotrys* (one isolate), and *Virgatospora* (one isolate) (Figure 5).**Figure 5.**
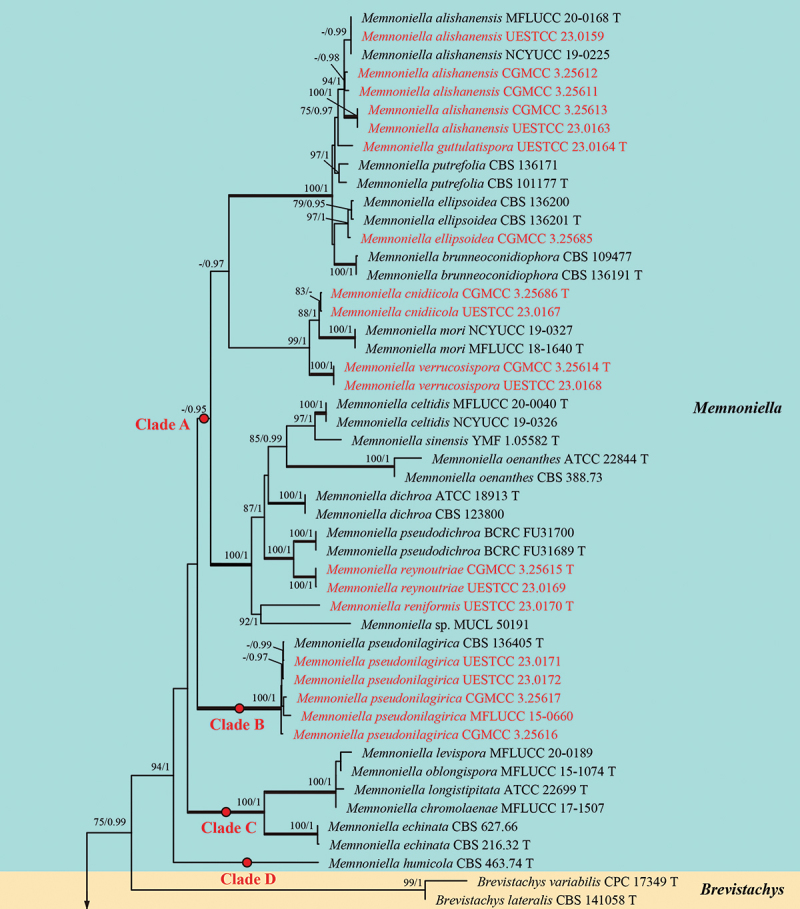
Phylogenetic tree constructed from maximum likelihood (RAxML) analyses of combined *cmdA*, ITS, *rpb2*, *tef1-α*, and *tub2* sequence data for selected genera within the family Stachybotryaceae (Hypocreales). Branches support for maximum likelihood (MLBS) equal to or greater than 75% and Bayesian inference posterior probabilities (BIPP) equal to or greater than 0.95 are marked above or below branches as MLBS/BIPP. The abbreviation T indicates the ex-type isolates. Species’ names and culture collections in red are newly collected taxa and synonymised isolates. The tree was rooted with *Fusarium sambucinum* Fuckel (CBS 146.95) and *Calonectria ilicicola* Boedijn & Reitsma (CBS 190.50).

The new species of *Memnoniella cnidiicola* (CGMCC 3.25686 and UESTCC 23.0167), *M. guttulatispora* (UESTCC 23.0164), *M. reniformis* (UESTCC 23.0170), *M. reynoutriae* (CGMCC 3.25615 and UESTCC 23.0169), and *M. verrucosispora* (CGMCC 3.25614 and UESTCC 23.0168), formed five distinct lineages within the genus *Memnoniella*, respectively. The isolate CGMCC 3.25685 was clustered together with *M. ellipsoidea* L. Lombard & Crous (CBS 136201, ex-type isolate). Five isolates (CGMCC 3.25611, CGMCC 3.25612, CGMCC 3.25613, UESTCC 23.0159, and UESTCC 23.0163) were nested with *M. alishanensis* Tennakoon, C.H. Kuo & K.D. Hyde (ex-type isolate MFLUCC 20-0168 and NCYUCC 19-0225) with good support (94% MLBS/1.00 BIPP). *Memnoniella nilagirica* (Subram.) C.G. Lin, Yong Wang bis & K.D. Hyde (MFLUCC 15-0660) was synonymised under *M. pseudonilagirica* L. Lombard & Crous. This taxon and the present four isolates (CGMCC 3.25616, CGMCC 3.25617, UESTCC 23.0171, and UESTCC 23.0172) grouped with ex-type isolate of *M. pseudonilagirica* (CBS 136405) with maximum support (100% MLBS/1.00 BIPP).

*Sirastachys ellipsoidispora* (CGMCC 3.25621 and UESTCC 23.0183) was sister to *Sir. phyllophila* L. Lombard & Crous (CBS 136169, ex-type isolate), and *Sir. aspidistrae* (CGMCC 3.25687 and UESTCC 23.0182) was sister to *Sir. phaeospora* L. Lombard & Crous (CBS 100155, ex-type isolate). They formed two distinct clades with 99% MLBS/1.00 BIPP and 95% MLBS/1.00 BIPP, respectively. Additionally, one isolate (UESTCC 23.0184) grouped with *Sir. castanedae* L. Lombard & Crous (CBS 136403, ex-type isolate) with strong support (100% MLBS/1.00 BIPP).

In *Striatibotrys*, five isolates (CGMCC 3.25619, CGMCC 3.25618, UESTCC 23.0175, UESTCC 23.0176, and UESTCC 23.0177) grouped together to form a strongly supported monophyletic clade (100% MLBS/1.00 BIPP), and one isolate (UESTCC 23.0180) clustered with *Str. rhabdospora* L. Lombard & Crous (CBS 528.80, ex-type isolate) with 100% MLBS/1.00 BIPP. In *Stachybotrys* and *Virgatospora*, two isolates UESTCC 23.0181 and UESTCC 23.0185 clustered together with *Sta. chartarum* (CBS 182.80, ex-type isolate) and *V. echinofibrosa* Finley (MUCL 39092 and CBS 110115), respectively.

### 3.2. Taxonomy

**Dothideomycetes** O.E. Erikss. & Winka, Myconet 1: 5 (1997)

**Pleosporales** Luttr. ex M.E. Barr, Prodromus to Class Loculoascomycetes (Amherst): 67 (1987)

**Dictyosporiaceae** Boonmee & K.D. Hyde, Fungal Diversity 80: 462 (2016)

***Dendryphiella*** Bubák & Ranoj., Annales Mycologici 12(4): 417 (1914)

*Notes*: *Dendryphiella* was first introduced by Ranojevic (1914), with *D. interseminata* (Berk. & Ravenel) Bubák & Ranoj. designated as the type species. This genus is characterised by macronematous, septate conidiophores featuring polytretic, integrated conidiogenous cells that are enlarged at the apex. The conidia are solitary or catenate, septate, and vary in colour from pale brown to brown, exhibiting a verrucose surface texture (Crous et al. 2014, 2016, 2019; Liu et al. 2017; Hyde et al. 2018). As of the latest records, *Dendryphiella* comprises 18 recognised species in the Species Fungorum (https://www.speciesfungorum.org/Names/Names.asp?strGenus=*Dendryphiella*; accessed on 9 December 2024).

**Figure 6.**
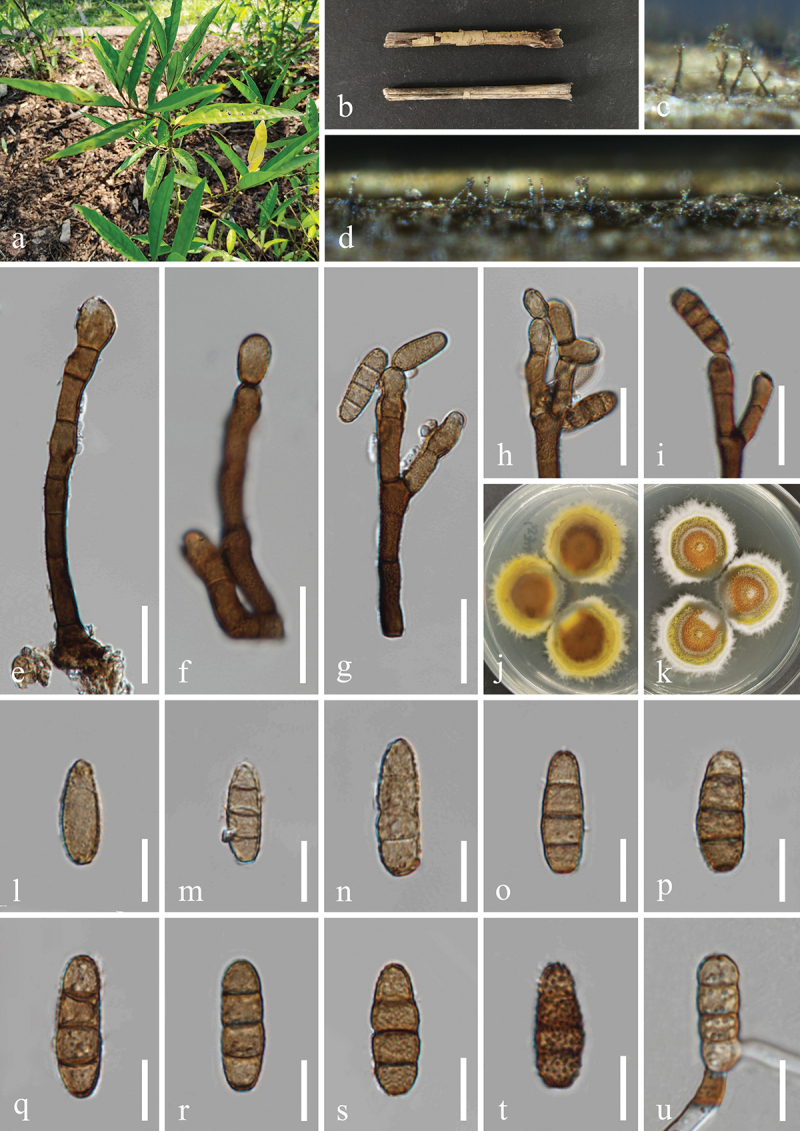
*Dendryphiella verrucosispora* (HKAS 132496, holotype). (a) Host *Justicia gendarussa*. (b) Twigs of *Justicia gendarussa*. (c, d) Sporulation on the host substrate. (e−i) Conidiophores, conidiogenous cells, and conidia. (j, k) Colonies on PDA, above (j) and below (k). (l−t) Conidia. (u) Germinated conidium. Scale bars: e−i = 20 µm, l−u =10 µm.

***Dendryphiella verrucosispora*** H.Z. Du & Jian K. Liu, sp. nov. Figure 6

MycoBank number: MB 855851.

*Etymology*: The epithet “*verrucosispora*” refers to the verrucose conidia.

**Description**: *Saprobic* on dead twigs of *Justicia gendarussa* (Acanthaceae). **Sexual morph**: Undetermined. **Asexual morph**: Hyphomycetous. *Colonies* on natural substrate superficial, effuse, dark brown, velvety. *Mycelium* partly immersed, partly superficial, composed of smooth, septate, branched, brown hyphae. *Conidiophores* macronematous, mononematous, simple or branched, erect, occasionally fasciculate, straight to flexuous, septate, brown to dark brown, paler towards the apex, wider at the sub-section, thick-walled, 45–80 μm ($\overset{ˉ}{x}$ = 72 μm, *n* = 30) long, 5–7 μm ($\overset{ˉ}{x}$ = 6 μm, *n* = 30) wide. *Conidiogenous cells* polytretic, terminal, integrated, brown, enlarged at the vertex, 7–13 μm long, 5–8 μm wide ($\overset{ˉ}{x}$ = 10 × 7 μm, *n* = 30). *Conidia* oblong, apex obtuse, base bluntly rounded, pale brown, aseptate when young, brown or dark brown, 3(4)-septate when mature, slightly constricted at septa, verrucose, 11−25 × 6−10 μm ($\overset{ˉ}{x}$ = 20 × 7 μm, *n* = 50).

**Culture characteristics**: Conidia germinated on PDA within 24 h, and germ tubes produced from basal cells. Colonies growing on PDA reached 32−35 mm in diameter after three weeks at 25 °C in the dark. Colonies from above, circular, medium dense, yellowish brown at the centre, with slightly yellow ring in the middle, white outer ring with filamentous and irregular margin. In reverse, brown to dark brown in the centre, with light yellow ring at the margin.

**Material examined**: CHINA, Yunnan Province, Xishuangbanna Dai Autonomous Prefecture, Jinghong City, Xishuangbanna South Medicinal Plant Garden, 100°47′17′′E, 22°0′24′′N, elevation 520 m, on dead twigs of medicinal plant *Justicia gendarussa* (Acanthaceae), 8 November 2022, H.Z. Du, S534 (HKAS 132496, holotype; HUEST 24.0222, isotype), ex-type living culture UESTCC 23.0504, ex-isotype living culture UESTCC 24.0205.

*Notes*: Multi-locus phylogenetic analyses indicated that *Dendryphiella verrucosispora* (UESTCC 23.0504 and UESTCC 24.0205) clustered with *D. fasciculata* N.G. Liu, Z.Y. Liu & K.D. Hyde (MFLUCC 17-1074, ex-type isolate), forming a distinct clade within the genus *Dendryphiella*, supported by strong statistical values (89% MLBS/1.00 BIPP) (Figure 3). Morphologically, *D. verrucosispora* and *D. fasciculata* are similar, both having macronematous conidiophores and 3-septate conidia. Notably, *D. fasciculata* is characterised by fasciculate conidiophores (Liu et al. 2017), while *D. verrucosispora* predominantly has solitary conidiophores. Additionally, *D. fasciculata* can be distinguished from *D. verrucosispora* by its larger conidiophores (170–250 μm vs. 45–80 μm) and conidiogenous cells (17–33 μm vs. 7–13 μm) (Liu et al. 2017). Comparative analyses of the LSU sequence nucleotides between *D. verrucosispora* (UESTCC 23.0504, ex-type isolate) and *D. fasciculata* (MFLUCC 17-1074) demonstrated a high similarity of 99.6% (801/804 bp), while the ITS locus exhibited 92.0% (481/523 bp) similarity. It is noteworthy that SSU and *tef1-α* sequences for *D. fasciculata* are currently unavailable. Following the criteria established by Jeewon and Hyde (2016), we introduce *D. verrucosispora* as a new species associated with *Justicia gendarussa.*

***Jalapriya*** D’souza, H.Y. Su, Z. Luo & K.D. Hyde, Fungal Diversity. 80: 476 (2016)

*Notes*: *Jalapriya* was described by Boonmee et al. (2016), with *J. pulchra* D’souza, H.Y. Su, Z. Luo & K.D. Hyde designated as the type species. This genus is characterised by the presence of dark brown to black colonies, acrogenous conidiophores, and solitary, cheiroid conidia (Boonmee et al. 2016). Currently, five species are recognised within *Jalapriya* in the Species Fungorum (https://www.speciesfungorum.org/Names/Names.asp?strGenus=*Jalapriya*; accessed on 9 December 2024), namely *J. apicalivaginata* D.F. Bao, X. Fu, H.Y. Su & Z.L. Luo, *J. aquatica* D.F. Bao, X. Fu, H.Y. Su & Z.L. Luo, *J. inflata* (Matsush.) D’souza, H.Y. Su, Z. Luo & K.D. Hyde, *J. pulchra*, and *J. toruloides* (Corda) D’souza, H.Y. Su, Z. Luo & K.D. Hyde. Species of *Jalapriya* have been found associated with unidentified submerged wood, as well as on *Laurus nobilis* and *Populus nigra*. Their distribution includes regions in Canada, China, Pakistan, and Spain (Matsushima 1983; Ahmad et al. 1997; Boonmee et al. 2021; Fu et al. 2021).

**Figure 7.**
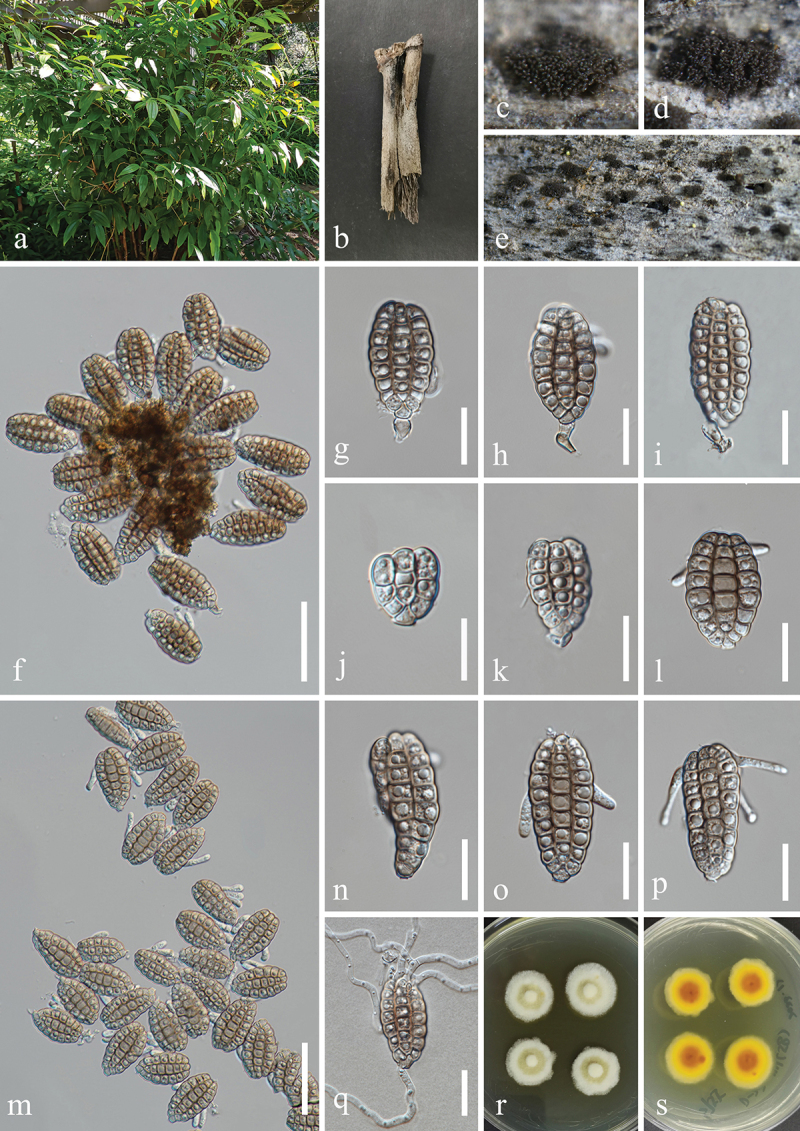
*Jalapriya cheirospora* (HKAS 132497, holotype). (a) Host *Disporum longistylum*. (b) Stem of *Disporum longistylum*. (c−e) Sporulation on the host substrate. (f) Squash mount of a sporodochium. (g−i) Conidiogenous cells and conidia. (j−p) Conidia. (q) Germinated conidium. (r, s) Colonies on PDA, above (r) and below (s). Scale bars: f, m = 50 µm, g−i, k, l, n−q = 20 µm, j = 15 µm.

***Jalapriya cheirospora*** H.Z. Du & Jian K. Liu, sp. nov. Figure 7

MycoBank number: MB 855852.

Etymology: The epithet “*cheirospora*” refers to the cheiroid conidia.

**Description**: *Saprobic* on dead stems of *Disporum longistylum* (Liliaceae). **Sexual morph**: Undetermined. **Asexual morph**: Hyphomycetous. *Colonies* on natural substrate punctiform, sporodochial, scattered, dark brown to black. *Mycelium* partly superficial, composed of brown to dark brown hyphae. *Conidiophores* micronematous, mononematous, septate, subglobose to cylindrical, unbranched, hyaline to pale brown. *Conidiogenous cells* holoblastic, integrated, terminal, hyaline to pale brown, 4.5–10 μm long, 4–6.5 μm wide ($\overset{ˉ}{x}$ = 7 × 5 μm, *n* = 30). *Conidia* acrogenous, solitary, cheiroid, pale to medium reddish brown, with 3 rows of cells, rows converging at apex, smooth-walled. Two outer rows arising from a basal cell, rows not separating, each row consisting of 4–10 cells, with guttulate, the size of outer rows 37–51 × 6–9 μm ($\overset{ˉ}{x}$ = 47 × 7 μm, *n* = 30), with 1–3 hyaline, tubular, elongated appendages, which are 10–30 × 4–7 μm ($\overset{ˉ}{x}$ = 17 × 5 μm, *n* = 30), attached to two outer rows, the size of inner rows 30–45 × 6–8 μm ($\overset{ˉ}{x}$ = 39 × 7 μm, *n* = 30). The size of conidia 37–56 × 20–26 μm ($\overset{ˉ}{x}$ = 46 × 24 μm, *n* = 30).

**Culture characteristics**: Conidia germinated on PDA within 24 h, and germ tubes produced from basal cells. Colonies growing on PDA reached 20−22 mm in diameter after three weeks at 25 °C in the dark. Colonies from above, circular with entire edge, medium dense, slightly elevated in the centre, white mycelium on the surface. In reverse, brown to yellowish-brown in the centre and bright yellow at the margin.

**Material examined**: CHINA, Yunnan Province, Kunming City, Panlong District, Kunming Botanical Garden, 102°44′24′′E, 25°8′27′′N, elevation 1,921 m, on dead stems of medicinal plant *Disporum longistylum* (H. Léveillé & Vaniot) H. Hara (Liliaceae), 11 November 2022, H.Z. Du, S755 (HKAS 132497, holotype; HUEST 24.0223, isotype); ex-type living culture UESTCC 23.0505; ex-isotype living culture UESTCC 24.0206.

*Notes*: Multi-locus phylogenetic analyses, incorporating available sequences from various *Jalapriya* species, indicate that *J. cheirospora* is most closely related to *J. aquatica* (Figure 3). Morphologically, *J. cheirospora* is characterised by tubular and elongated appendages that are attached to two outer cell rows, in contrast to the cap-like or spherical appendages of *J. aquatica*, which are affixed to the apical cells of the conidia (Fu et al. 2021). Sequence comparisons between the ex-type isolates of *J. cheirospora* (UESTCC 23.0505) and *J. aquatica* (CGMCC 3.20613), revealed similarities of 97.1% (464/478 bp) in the ITS region, 99.9% (774/775 bp) in the LSU region, and 96.83% (886/915 bp) in the *tef1-α* region. Therefore, *J. cheirospora* is introduced as a new species based on both morphological characteristics and phylogenetic evidence, with an association to the medicinal plant *Disporum longistylum* in China.

**Melanommataceae** G. Winter, Rabenhorst’s Kryptogamen-Flora von Deutschland, Österreich und der Schweiz, Edn 2 1(2): 220 (1885)

***Camposporium*** Harkn., Bulletin of the California Academy of Sciences 1: 37 (1884)

*Notes*: *Camposporium* was established by Harkness (1884) with a single species, *C. antennatum*, while *Fusiconidium* Jin F. Li, Phookamsak & K.D. Hyde was introduced by Li et al. (2017) with *F. mackenziei* Jin F. Li, Phookamsak, Camporesi & K.D. Hyde as the type species. *Camposporium* was placed in Melanommataceae by Hyde et al. (2020a), who differentiated *Camposporium* and *Fusiconidium* as distinct genera based on variations in conidial shape and the presence of apical appendages. However, Koukol and Delgado (2021) subsequently regarded *Camposporium* and *Fusiconidium* as congeneric, synonymising *Fusiconidium* under *Camposporium*.

**Figure 8.**
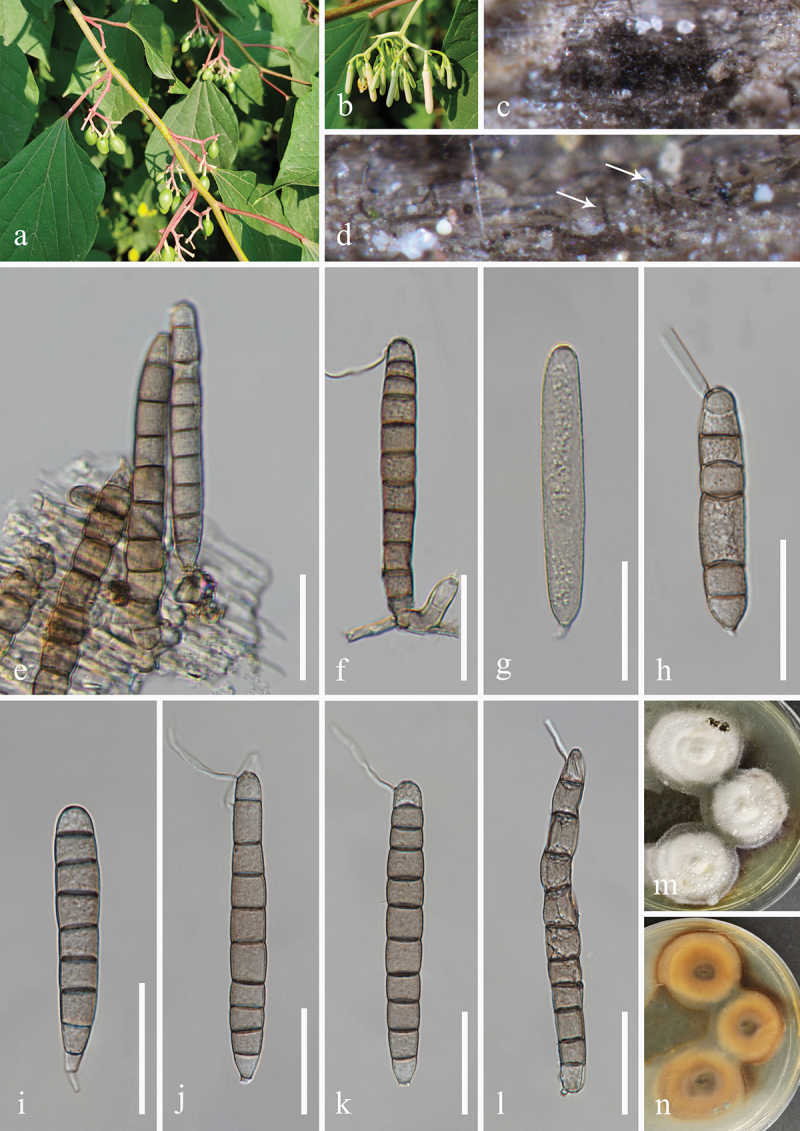
*Camposporium alangii* (HKAS 132500, holotype). (a, b) Host *Alangium chinense*. (c, d) Sporulation on the host substrate. (e, f) Conidiophores and conidia. (g−l) Conidia. (m, n) Colonies on PDA, above (m) and below (n). Scale bars: e−l = 30 µm.

***Camposporium alangii*** H.Z. Du & Jian K. Liu, sp. nov. Figure 8

MycoBank number: MB 855854.

Etymology: The epithet “*alangii*” refers to the host genus *Alangium* from which the fungus was originally isolated.

**Description**: *Saprobic* on dead branches of *Alangium chinense* (Cornaceae). **Sexual morph**: Undetermined. **Asexual morph**: Hyphomycetous. *Colonies* on natural substrate effuse, dark brown to black, hairy. *Mycelium* mostly immersed, composed of brown, septate, branched, smooth hyphae. *Conidiophores* micronematous to semi-macronematous, mononematous, solitary, short, unbranched, subhyaline to pale brown, thick-walled, straight or flexuous, irregularly cylindrical, 1–2 septate, slightly constricted at septa, often procumbent on substrate, 8–12 μm ($\overset{ˉ}{x}$ = 10 μm, *n* = 20) long, 4–6 μm ($\overset{ˉ}{x}$ = 5 μm, *n* = 20) wide. *Conidiogenous cells* monoblastic, terminal, integrated, subcylindrical, pale brown, 3.5–4.5 μm long, 1.5–3 μm wide ($\overset{ˉ}{x}$ = 4 × 2 μm, *n* = 30). *Conidia* solitary, cylindrical, with rounded apex and subtruncate base, brown, with polar cells pale brown to subhyaline, 6–10-septate, slightly constricted at the septa, smooth on the conidia surface, 52–90 × 9–12 μm ($\overset{ˉ}{x}$ = 77 × 10 μm, *n* = 30), with a single long, filiform, hyaline, aseptate, smooth appendage at the apex, tapering from the base to the tip, 25–40 × 1.8–3.3 μm ($\overset{ˉ}{x}$ = 31 × 2.5 μm, *n* = 20).

**Culture characteristics**: Conidia germinated on PDA within 24 h, and germ tubes produced from basal cells. Colonies growing on PDA reached 33−35 mm in diameter after three weeks at 25 °C in the dark. Colonies from above, circular, medium dense, white mycelium on the surface with regular margin. In reverse, dark brown in the centre and brown at the margin.

**Material examined**: CHINA, Guizhou Province, Guiyang City, Huaxi District, 106°41′48′′E, 26°32′18′′N, elevation 1,120 m, on dead branches of medicinal plant *Alangium chinense* (Cornaceae), 22 February 2021, H.Z. Du, S81-1 (HKAS 132500, holotype; HUEST 24.0225, isotype); ex-type living culture UESTCC 23.0509; ex-isotype living culture UESTCC 24.0208.

*Notes*: Multi-locus phylogenetic analyses indicate that *Camposporium alangii* (UESTCC 23.0509 and UESTCC 24.0208) is closely related to *C. antennatum* (ICMP 15321), supporting its recognition as a distinct phylogenetic species (Figure 4). Morphologically, *C. alangii* shares similarities with *C. antennatum*, characterised by short, simple conidiophores, monoblastic terminal conidiogenous cells, and cylindrical, septate conidia with apical appendages. Notably, *C. alangii* possesses a single appendage at the apex, in contrast to the two appendages observed in *C. antennatum*. Furthermore, comparative analysis of the ITS sequences between the ex-type isolates reveals a nucleotide similarity of 90.6% (451/498 bp) between *C. alangii* (UESTCC 23.0509) and *C. antennatum* (ICMP 15321). Currently, twenty-five valid species of *Camposporium* is listed in the Species Fungorum (https://www.speciesfungorum.org/Names/Names.asp?strGenus=*Camposporium*; accessed on 9 December 2024), of which thirteen species have corresponding DNA sequence data available in GenBank (https://www.ncbi.nlm.nih.gov/nuccore/?term=*Camposporium*+; accessed on 9 December 2024). Based on the morphological comparisons detailed in Table 3, *C. alangii* is distinguished from other *Camposporium* species lacking DNA sequence data. Consequently, the new species *C. alangii* is introduced based on both morphological characteristics and phylogenetic evidence.

**Table 3.** Comparisons of morphological characteristics between *Camposporium alangii* and other *Camposporium* species that lack DNA sequence data.

Species	Conidiophores		Conidia			References
	Size (μm)	Septation	Size (μm)	Septation	Appendages	
*C. alangii*	8–12 × 4–6	1–2	52–90 × 9–12	6–10	1 (simple)	In this study
*C. chinense*	220–400 × 7–9.5	8–16	110–160 × 12.5–16	9–12	1 (simple)	Xu et al. (2021)
*C. fusisporum*	100–145 × 6.5–10	10–15	86–115 × 13.5–19	8–11	2–3 (simple)	Whitton et al. (2002)
*C. himalayanum*	74–131 × 6–8	7–12	75.4–85.7 × 7–9	7–10	0–1 (simple)	Adamčík et al. (2015)
*C. hyderabadense*	25.2–39.6 × 3.6–5.4	1–3	32.4–54 × 3.6–7.2	5–9	1–4 (simple)	Rao and Rao (1964)
*C. indicum*	28.5–50.4 × 3.6–7.2	2–5	21.6–72 × 3.6–7.2	3–14	0	Rao and Rao (1964)
*C. japonicum*	37.5–77.5 × 5–6.5	5	42.5–70 × 5–7.5	7–10	0–1 (2–4 branched)	Ichinoe (1971)
*C. laundonii*	Up to 40 × 5–8	0–2	50–150 × 13–17	4–9	1–2 (simple)	Ellis (1976)
*C. microsporum*	Up to 72 × 3.6–7.2	1–5	25.8–36 × 7.2–9	2–6	1–2 (simple)	Rao and Rao (1964)
*C. ontariense*	45–200 × 5–7	6–8	20–53 × 6.5–12	3–9	0	Matsushima (1983)
*C. paubrasiliae*	Very short	0	77.5–117.5 × 10–15	5–7	0	Alves and Gusmão (2024)
*C. quercicola*	15–60 × 3.5–4	1–3	28–45 × 3.5–4.5	5–9	0–3 (simple)	Mercado-Sierra et al. (1995)
*C. scolecosporium*	10–20 × 2.5–4.5	0–3	48–108 × 3–4	6–12	0	Kobayasi (1971)

**Figure 9.**
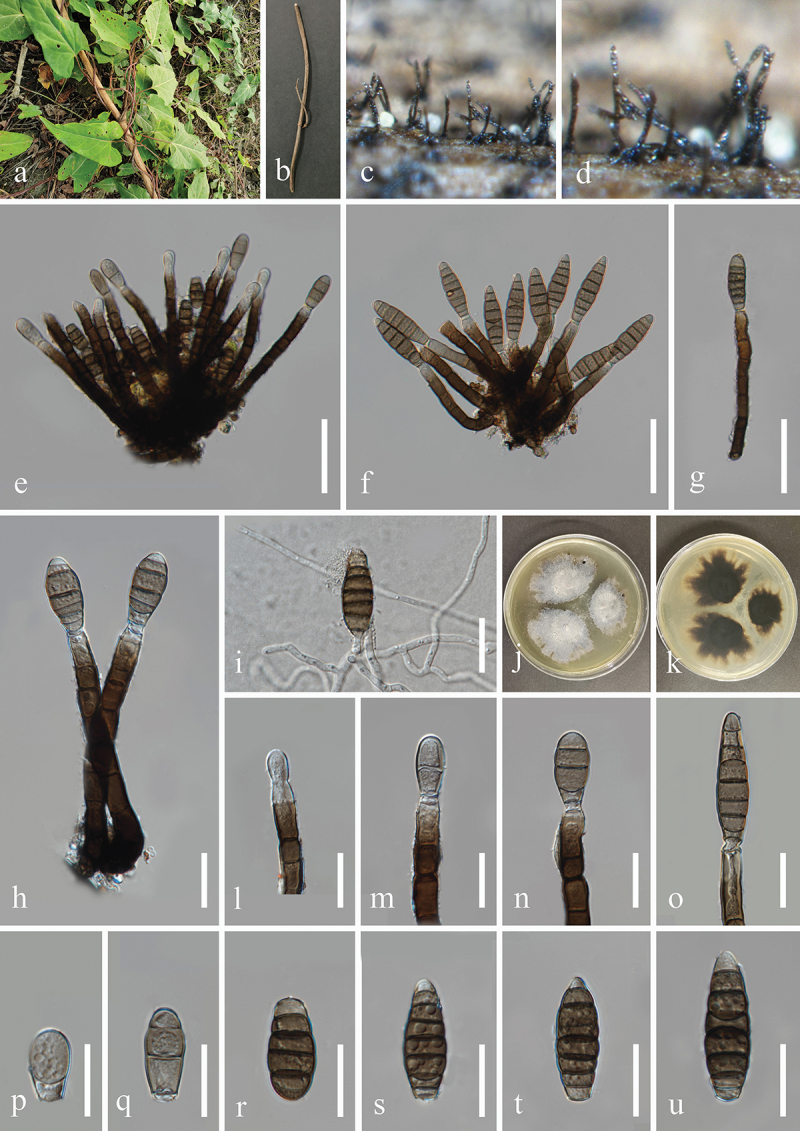
*Camposporium polygoni* (HKAS 132498, holotype). (a) Host *Polygonum multiflorum*. (b) Vine of *Polygonum multiflorum*. (c, d) Sporulation on the host substrate. (e−h) Conidiophores, conidiogenous cells, and conidia. (i) Germinated conidium. (j, k) Colonies on PDA, above (j) and below (k). (l−o) Conidiogenous cells and conidia. (p−u) Conidia. Scale bars: e−g = 50 µm, h, i, l−u = 20 µm.

***Camposporium polygoni*** H.Z. Du & Jian K. Liu, sp. nov. Figure 9

MycoBank number: MB 855853.

Etymology: The epithet “*polygoni*” refers to the host genus *Polygonum* from which the fungus was originally isolated.

**Description**: *Saprobic* on dead vines of *Polygonum multiflorum* (Polygonaceae). **Sexual morph**: Undetermined. **Asexual morph**: Hyphomycetous. *Colonies* on natural substrate superficial, effuse, brown to dark brown, velvety. *Mycelium* immersed, composed of brown, septate, branched, smooth hyphae. *Conidiophores* macronematous, mononematous, brown to dark brown, paler towards the apex, caespitose, unbranched, erect, straight or flexuous, thick-walled, cylindrical, with 5–10 transverse septa, 50–90 μm ($\overset{ˉ}{x}$ = 70 μm, *n* = 30) long, 7–9 μm ($\overset{ˉ}{x}$ = 8 μm, *n* = 30) wide. *Conidiogenous cells* monoblastic, terminal, integrated, hyaline to pale brown, 15–20 μm long, 7–10 μm wide ($\overset{ˉ}{x}$ = 17 × 8 μm, *n* = 30). *Conidia* acrogenous, solitary, straight or slightly curved, fusiform to ellipsoidal, with rounded apex and truncate base, initially subhyaline to pale brown, aseptate, brown to dark brown, paler at apical cell, 4−7-septate at maturity, slightly constricted at the septa, smooth on the conidia surface, without sheaths or appendages, 23−35 × 8−14 μm ($\overset{ˉ}{x}$ = 30 × 11 μm, *n* = 50).

**Culture characteristics**: Conidia germinated on PDA within 24 h, and germ tubes produced from basal cells. Colonies growing on PDA reached 37−40 mm in diameter after three weeks at 25 °C in the dark. Colonies from above, circular with lobate edge, flat, slightly raised, medium dense, greyish mycelium on the surface. In reverse, dark brown to black in the centre and pale brown at the margin.

**Material examined**: CHINA, Guizhou Province, Guiyang City, Nanming District, Guiyang Medicinal Botanical Garden, 106°41′48′′E, 26°32′18′′N, elevation 1,127 m, on dead vines of medicinal plant *Polygonum multiflorum* (Polygonaceae), 8 May 2021, H.Z. Du, S255 (HKAS 132498, holotype; HUEST 24.0224, isotype); ex-type living culture UESTCC 23.0506, ex-isotype living culture UESTCC 24.0207.

*Notes*: Multi-locus phylogenetic analyses revealed that *Camposporium polygoni* (UESTCC 23.0506 and UESTCC 24.0207) forms a distinct lineage, clustering with *C. valdivianum* (MFLUCC 14-0434 and HKAS 95019) and *C. pini* (CGMCC 3.27521) with strong support (99% MLBS/1.00 BIPP) (Figure 4). Morphologically, *C. polygoni* is similar to *C. valdivianum* and *C. pini* in having brown, septate, unbranched, cylindrical conidiophores and septate, fusiform to ellipsoidal conidia (Li et al. 2017; Tian et al. 2024). However, *C. polygoni* has smaller conidiophores (50–90 × 7–9 μm) and conidia (23−35 × 8−14 μm) compared to *C. valdivianum* (conidiophores: 91–114 × 8.5–11 µm, conidia: 38–40 × 12–15 µm) and *C. pini* (conidiophores: 95–180 × 7–11 μm, conidia: 27–50 × 10–14 μm) (Li et al. 2017; Tian et al. 2024). Sequence comparison of the ex-type isolates reveals 97.5% similarity (459/471 bp) in the ITS region, 99.0% (910/919 bp) in the *tef1-α* gene, and 97.7% (769/787 bp) in the *rpb2* gene between *C. polygoni* (UESTCC 23.0506) and *C. pini* (CGMCC 3.27521). Furthermore, *C. polygoni* shows 99.1% similarity (831/839 bp) in the *tef1-α* sequence and 95.9% similarity (1,016/1,059 bp) in the *rpb2* sequence when compared to *C. valdivianum* (MFLUCC 14-0434, ex-type isolate). Therefore, *C. polygoni*, associated with the medicinal plant *Polygonum multiflorum*, is introduced as a new species in China.

**Sordariomycetes** O.E. Erikss. & Winka, Myconet 1: 10 (1997)

**Hypocreales** Lindau, Die Natürlichen Pflanzenfamilien nebst ihren Gattungen und wichtigeren Arten 1(1): 343 (1897)

**Stachybotryaceae** L. Lombard & Crous, Persoonia 32: 283 (2014)

***Memnoniella*** Höhn., Zentralblatt für Bakteriologie, Parasitenkunde, Infektionskrankheiten und Hygiene, 2. Abt. 60(1/6): 16 (1923)

*Notes*: *Memnoniella* was established by von Höhnel (1924), with *M. echinata* (Rivolta) Galloway as the type species. Morphologically, *Memnoniella* is characterised by macronematous, mononematous conidiophores, phialidic, determinate, terminal conidiogenous cells bearing unicellular, smooth to verrucose conidia in dry chains or slimy masses (Ellis 1971; Jong and Davis 1976; Photita et al. 2003; Pinruan et al. 2004; Manoharachary et al. 2006; Seifert and Gams 2011; Wang et al. 2015; Lombard et al. 2016; Zheng et al. 2019; Samarakoon et al. 2021). Despite previous suggestions of synonymy between *Memnoniella* and *Stachybotrys* (Smith 1962; Wang et al. 2015), Lombard et al. (2016) resurrected *Memnoniella* as a distinct genus in Stachybotryaceae, supported by subsequent studies (Lin et al. 2016; Doilom et al. 2017; Hyde et al. 2020b; Mapook et al. 2020). Currently, seventeen valid species have been accepted in the Species Fungorum (https://www.speciesfungorum.org/Names/Names.asp?strGenus=*Memnoniella*; accessed on 9 December 2024).

***Memnoniella alishanensis*** Tennakoon, C.H. Kuo & K.D. Hyde, Fungal Diversity 108: 168 (2021) Figure 10

**Figure 10.**
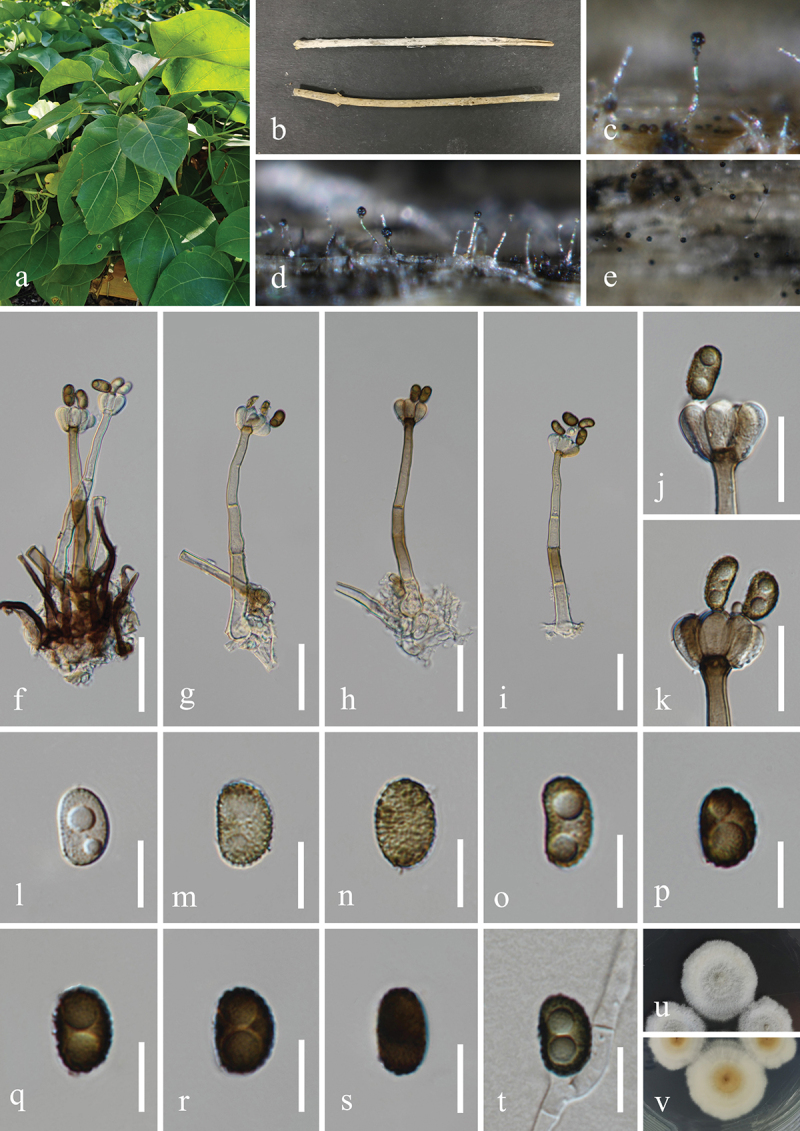
*Memnoniella alishanensis* (HUEST 23.0159, new host record). (a) Host *Dregea volubilis*. (b) Twigs of *Dregea volubilis*. (c−e) Sporulation on the host substrate. (f−i) Conidiophores and conidia. (j, k) Conidiogenous cells and conidia. (l−s) Conidia. (t) Germinated conidium. (u, v) Colonies on PDA, above (u) and below (v). Scale bars: f−i = 40 µm, j, k = 20 µm, l−t =10 µm.

**Description**: *Saprobic* on dead twigs of *Dregea volubilis* (Apocynaceae). **Sexual morph**: Undetermined. **Asexual morph**: Hyphomycetous. *Colonies* on natural substrate effuse, glistening, black, single or clustered, globose to subglobose at the apex. *Mycelium* immersed, partly superficial. *Conidiophores* macronematous, mononematous, simple, erect, straight to flexuous, unbranched, smooth, thick-walled, 2–4-septate, subhyaline to light brown at the base, olive grey to or brown at the apex, 100–150 µm ($\overset{ˉ}{x}$ = 125 µm, *n* = 30) long, 8–14 µm ($\overset{ˉ}{x}$ = 11 μm, *n* = 30) wide at the base, tapering to 4–6 µm ($\overset{ˉ}{x}$ = 5 μm, *n* = 30) wide at the narrowest point near the apex, bearing 4–6 conidiogenous cells at its apex. *Conidiogenous cells* ellipsoidal, obovate, clavate or reniform, monophialidic, discrete, determinate, terminal, smooth, clustered at the apex of conidiophores, subhyaline to light brown, 9–15 µm ($\overset{ˉ}{x}$ = 13 µm, *n* = 50) long, 6–9 µm ($\overset{ˉ}{x}$ = 7 μm, *n* = 50) wide at the widest part. *Conidia* aggregated in black and glistening heads, ellipsoidal, reniform or comma shaped, acrogenous, aseptate, verrucose, 1–2 guttules in each cell, thick-walled, some with mesh texture, rounded at both ends, olivaceous to brown when young, deep brown to black when mature, 10–13 × 6–9 µm ($\overset{ˉ}{x}$ = 12 × 7 µm, *n* = 50).

**Culture characteristics**: Conidia germinated on PDA within 48 h, and germ tubes produced from basal cells. Colonies growing on PDA reached 30−32 mm in diameter after one month at 25 °C in the dark. Colonies from above, white in the whole colony, circular with entire edge, medium dense, surface smooth, fluffy to velvety with smooth aspects, slightly elevated at the central point, light brown to white from centre to the edge in reverse.

**Material examined**: CHINA, Yunnan Province, Xishuangbanna Dai Autonomous Prefecture, Xishuangbanna Tropical Botanical Garden Chinese Academy of Sciences, 101°15′6′′E, 21°55′51′′N, elevation 505 m, H.Z. Du, 9 November 2022, on dead twigs of medicinal plant *Dregea volubilis* (Apocynaceae), S574 (HUEST 23.0159), living culture UESTCC 23.0159; *ibid*., on dead twigs of medicinal plant *Baliospermum solanifolium* (Euphorbiaceae), S634 (HKAS 131296), living culture CGMCC 3.25612; *ibid*., 10 November 2022, on dead twigs of medicinal plant *Disporopsis longifolia* (Asparagaceae), S683 (HUEST 23.0163), living culture UESTCC 23.0163; *ibid*., on dead twigs of medicinal plant *Justicia brandegeeana* (Acanthaceae), S682 (HKAS 131297), living culture CGMCC 3.25613; *ibid*., Sichuan Province, Leshan City, Muchuan County, Huangdan Town, 103°41′37′′E, 29°12′58′′N, elevation 555 m, H.Z. Du & N. Wu, 31 October 2021, on dead twigs of medicinal plant *Alangium chinense* (Cornaceae), S465 (HKAS 131295), living culture CGMCC 3.25611.

*Notes*: *Memnoniella alishanensis* was introduced by Tennakoon et al. (2021) and isolated from dead leaves of *Macaranga tanarius* (L.) Muell. Arg. (Euphorbiaceae). In this study, our five specimens (HUEST 23.0159, HKAS 131296, HUEST 23.0163, HKAS 131297, and HKAS 131295) are morphologically similar to the holotype (MFLU 18-0088) of *M. alishanensis*. Multi-locus phylogeny showed that our isolates (CGMCC 3.25611, CGMCC 3.25612, CGMCC 3.25613, UESTCC 23.0159, and UESTCC 23.0163) clustered together with the ex-type isolate (MFLUCC 20-0168) of *M. alishanensis* and formed strongly supported clade (94% MLBS/1.00 BIPP) (Figure 5). Therefore, we identify these five isolates as *M. alishanensis* based on morphology and phylogeny, and this is the first report of *M. alishanensis* from medicinal plants of *Alangium chinense*, *Baliospermum solanifolium*, *Disporopsis longifolia*, *Dregea volubilis*, and *Justicia brandegeeana* in China.

**Figure 11.**
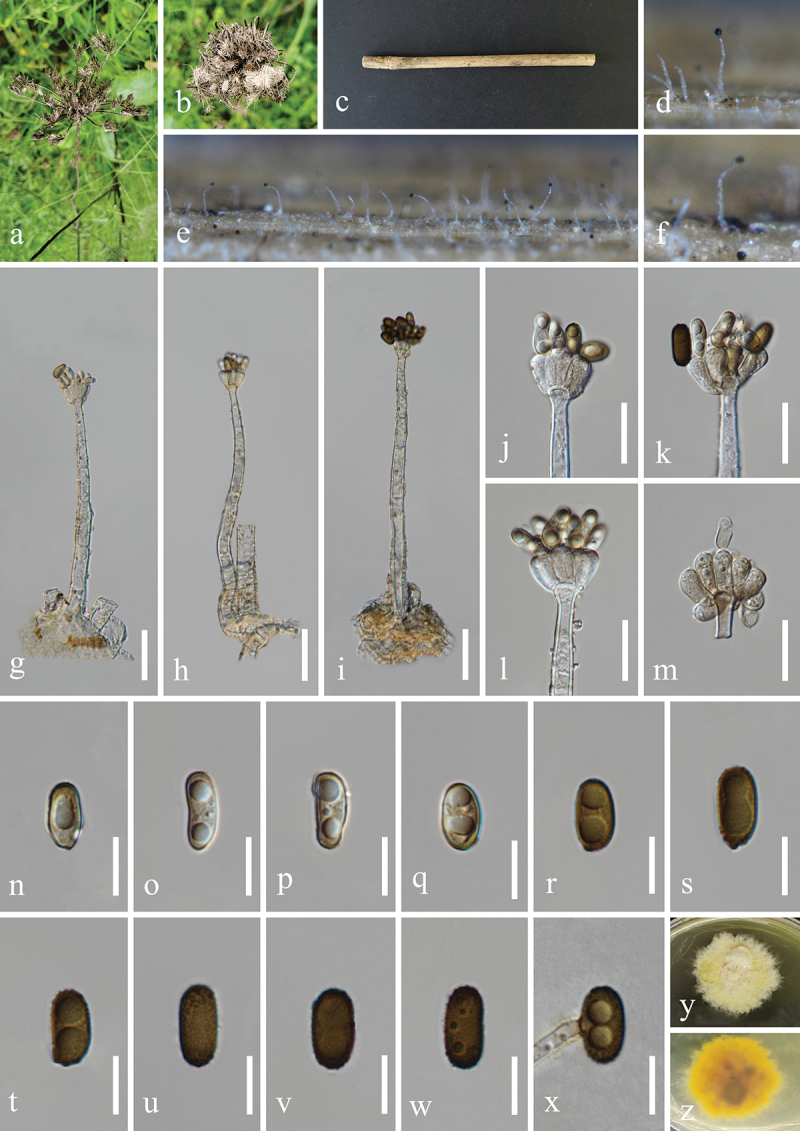
*Memnoniella cnidiicola* (HKAS 131300, holotype). (a) Host *Cnidium monnieri*. (b) Fruits of *Cnidium monnieri*. (c) Stem of *Cnidium monnieri*. (d−f) Sporulation on the host substrate. (g−i) Conidiophores and conidia. (j−m) Conidiogenous cells and conidia. (n−w) Conidia. (x) Germinated conidium. (y, z) Colony on PDA, above (y) and below (z). Scale bars: g−i = 40 µm, j−m = 20 µm, n−x =10 µm.

***Memnoniella cnidiicola*** H.Z. Du & Jian K. Liu, sp. nov. Figure 11

MycoBank number: MB 854195.

Etymology: The epithet “*cnidiicola*” refers to the host genus *Cnidium* from which the fungus was originally isolated.

**Description**: *Saprobic* on dead stems of *Cnidium monnieri* (Apiaceae). **Sexual morph**: Undetermined. **Asexual morph**: Hyphomycetous. *Colonies* on natural substrate effuse, single or clustered, black at the apex. *Mycelium* immersed, partly superficial. *Conidiophores* macronematous, mononematous, simple, erect, straight to slightly flexuous, slightly verrucose, thick-walled, unbranched, 2–4-septate, hyaline to subhyaline, 180–236 µm ($\overset{ˉ}{x}$ = 207 µm, *n* = 30) long, 11–16 µm ($\overset{ˉ}{x}$ = 14 µm, *n* = 30) wide at the base, tapering to 5–8 µm ($\overset{ˉ}{x}$ = 6 µm, *n* = 30) wide at the narrowest point near the apex, bearing 4–6 conidiogenous cells at its apex. *Conidiogenous cells* clavate, obovate, monophialidic, discrete, determinate, terminal, smooth, subhyaline to olivaceous brown, 11–18 µm ($\overset{ˉ}{x}$ = 14 µm, *n* = 50) long, 5.5–9 µm ($\overset{ˉ}{x}$ = 7 μm, *n* = 50) wide at the widest part. *Conidia* aggregated in black heads, ellipsoidal, crogenous, aseptate, mostly with 2 large guttules, occasionally a single large ellipsoid guttule, thick-walled, rounded at both ends or slightly narrowing to a central or oblique base, olive grey to olivaceous brown when young, olivaceous brown to dark brown when mature, 9–14 × 5–8 µm ($\overset{ˉ}{x}$ = 12.5 × 7 μm, *n* = 50).

**Culture characteristics**: Conidia germinated on PDA within 24 h, and germ tubes produced from basal cells. Colonies growing on PDA reached 18−20 mm in diameter after one month at 25 °C in the dark. Colonies from above, circular with filamentous edge, yellowish white in the whole colony with mucinoid substances, bright yellow in the whole colony with some brown patches in reverse, light yellow pigmentation on PDA.

**Material examined**: CHINA, Sichuan Province, Chengdu City, Dujiangyan City, Qingcheng Mountain scenic spot, 103°28′36′′E, 30°55′9′′N, elevation 1,178 m, 27 March 2021, H.Z. Du & N. Wu, on dead stems of medicinal plant *Cnidium monnieri* (Apiaceae), S168 (HKAS 131300, holotype; HUEST 23.0166, isotype), ex-type living culture CGMCC 3.25686; ex-isotype living culture UESTCC 23.0166; *ibid*., on dead stems of medicinal plant *Gynostemma pentaphyllum* (Cucurbitaceae), S167 (HUEST 23.0167, paratype), ex-paratype living culture UESTCC 23.0167.

*Notes*: *Memnoniella cnidiicola* and *M. mori* Tennakoon, C.H. Kuo & K.D. Hyde are phylogenetically close in our multi-locus analyses (Figure 5). Both species share similar morphology by having macronematous and mononematous conidiophores, obovate and monophialidic conidiogenous cells and oblong conidia with guttles (Tennakoon et al. 2021). However, *M. cnidiicola* has larger conidiophores (180 − 236 × 11–16 µm vs. 70 − 110 × 4–6.5 µm) and conidiogenous cells (11–18 × 5.5–9 µm vs. 9–12 × 4–6 µm) than those of *M. mori*. Additionally, the comparison of nucleotide base pair between *M. cnidiicola* (CGMCC 3.25686, ex-type isolate) and *M. mori* (MFLUCC 18-1640, ex-type isolate) revealed 98.0% (534/545 bp) of ITS and 96.6% (282/292 bp) of *tub2* similarity. Therefore, the establishment of the new species *M. cnidiicola* is justified by phylogenetic and morphologic evidence, which is associated with medicinal plants *Cnidium monnieri* and *Gynostemma pentaphyllum* in China.

***Memnoniella ellipsoidea*** L. Lombard & Crous, Persoonia 36: 197 (2016) Figure 12

**Figure 12.**
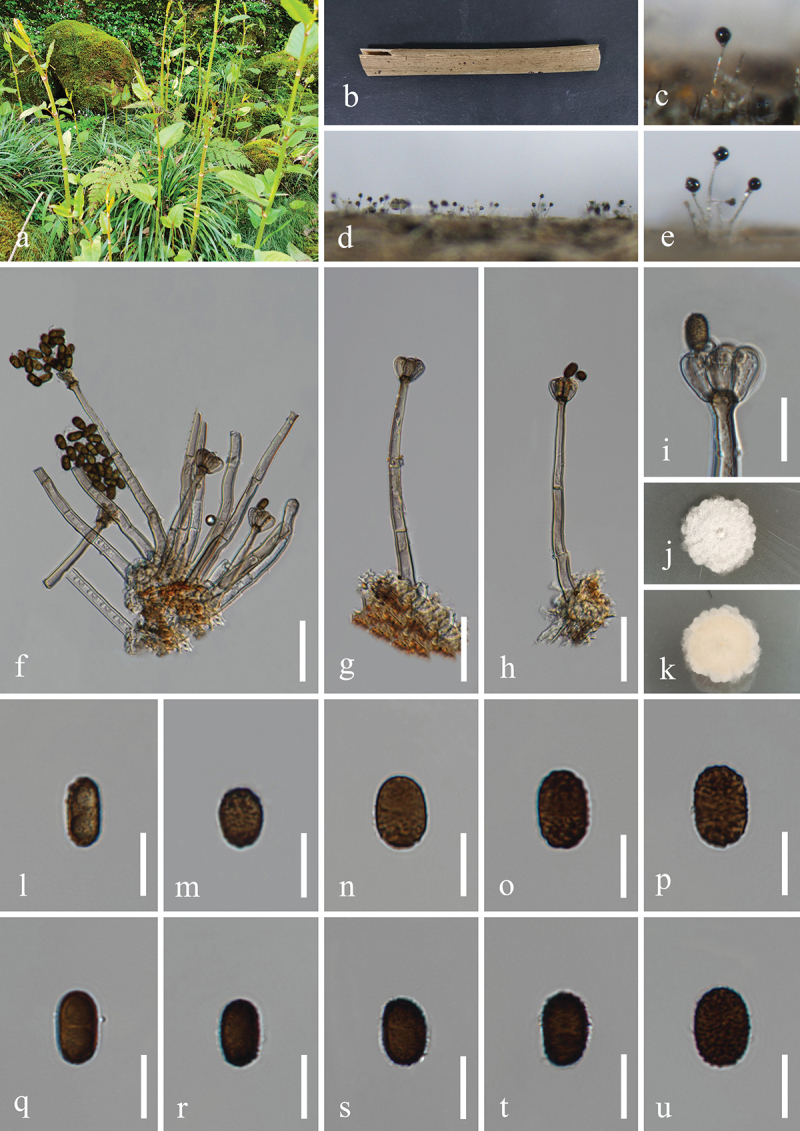
*Memnoniella ellipsoidea* (HKAS 131299, new host and geographical records). (a) Host *Reynoutria japonica*. (b) Stem of *Reynoutria japonica*. (c−e) Sporulation on the host substrate. (f−h) Conidiophores and conidia. (i) Conidiogenous cells and conidia. (j, k) Colony on PDA, above (j) and below (k). (l−u) Conidia. Scale bars: f−h = 40 µm, i = 15 µm, l−u =10 µm.

**Description**: *Saprobic* on dead stems of *Reynoutria japonica* (Polygonaceae). **Sexual morph**: Undetermined. **Asexual morph**: Hyphomycetous. *Colonies* on natural substrate effuse, glistening, black, single or clustered, globose at the apex. *Mycelium* immersed, partly superficial. *Conidiophores* macronematous, mononematous, simple, erect, straight to slightly flexuous, smooth, thick-walled, unbranched, 2–4-septate, hyaline to subhyaline, becoming olivaceous brown towards the apex, 115–145 µm ($\overset{ˉ}{x}$ = 132 µm, *n* = 30) long, 8–11 µm ($\overset{ˉ}{x}$ = 10 µm, *n* = 30) wide at the base, tapering to 4–6.5 µm ($\overset{ˉ}{x}$ = 5 µm, *n* = 30) wide at the narrowest point near the apex, bearing 4–6 conidiogenous cells at its apex. *Conidiogenous cells* clavate, obovate, monophialidic, discrete, determinate, terminal, smooth, subhyaline to olivaceous brown, 10–14 µm ($\overset{ˉ}{x}$ = 12 µm, *n* = 50) long, 4–8 µm ($\overset{ˉ}{x}$ = 6 μm, *n* = 50) wide at the widest part. *Conidia* aggregated in black and glistening heads, ellipsoidal, acrogenous, aseptate, with 2 globose guttules, verrucose, thick-walled, rounded at both ends, olive grey to olivaceous brown when young, dark brown to black when mature, 9–13 × 5–9 µm ($\overset{ˉ}{x}$ = 11 × 7 µm, *n* = 50).

**Culture characteristics**: Conidia germinated on PDA within 48 h, and germ tubes produced from basal cells. Colonies growing on PDA reached 18−19 mm in diameter after one month at 25 °C in the dark. Colonies from above, circular with curled edge, white in the whole colony and not smooth. In reverse, slightly brown in the whole colony.

**Material examined**: CHINA, Sichuan Province, Chengdu City, High-tech West District, Yaobo Park, 103°56′21′′E, 30°43′57′′N, elevation 504 m, on dead stems of medicinal plant *Reynoutria japonica* (Polygonaceae), 11 August 2021, H.Z. Du, S349 (HKAS 131299), living culture CGMCC 3.25685.

*Notes*: *Memnoniella ellipsoidea* was introduced by Lombard et al. (2016) and isolated from dead twigs of *Bromelia* sp. in Nepal. One collection (HKAS 131299) is morphologically similar to the holotype (CBS H-22448) of *M. ellipsoidea* (Lombard et al. 2016). Multi-locus phylogeny showed that one isolate (CGMCC 3.25685) grouped with *M. ellipsoidea* (CBS 136201, ex-type isolate) by 97% MLBS/1.00 BIPP (Figure 5). Therefore, we identify our isolate as *M. ellipsoidea* and this is a new host and geographical record from medicinal plant *Reynoutria japonica* in China.

**Figure 13.**
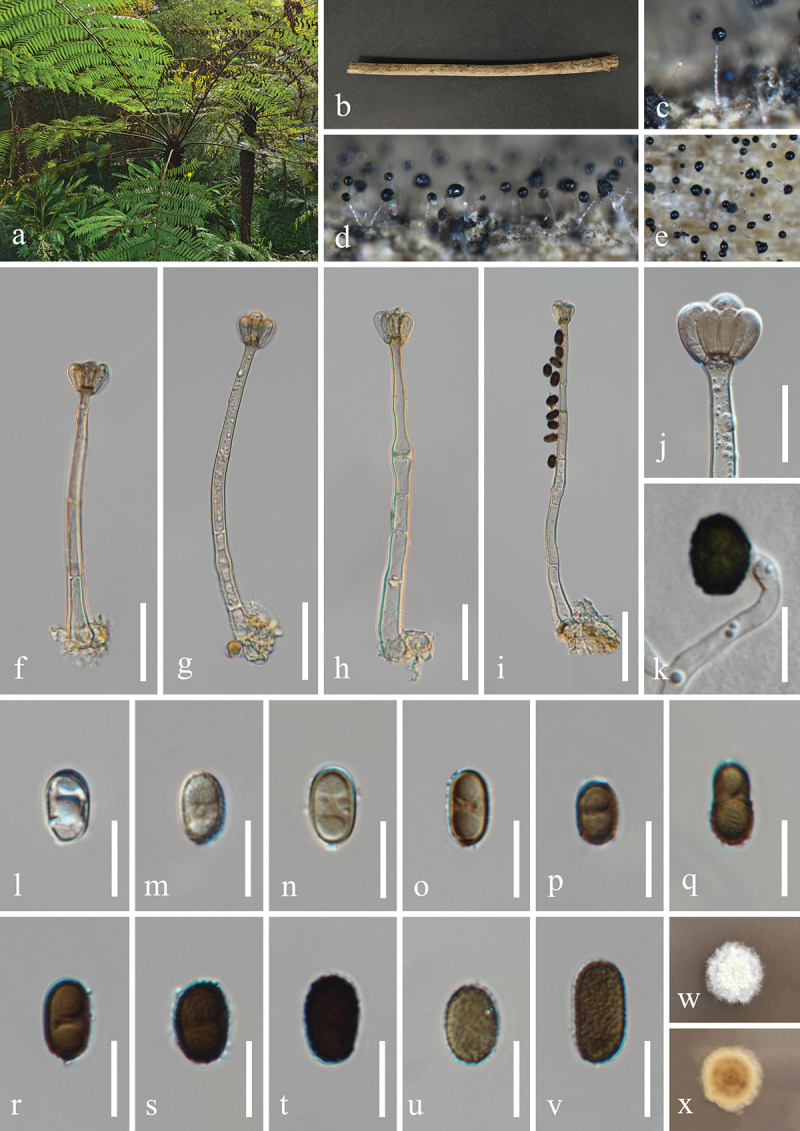
*Memnoniella guttulatispora* (HKAS 131298, holotype). (a) Host *Alsophila spinulosa*. (b) Petiole of *Alsophila spinulosa*. (c–e) Sporulation on the host substrate. (f−i) Conidiophores and conidia. (j) Conidiogenous cells. (k) Germinated conidium. (l−v) Conidia. (w, x) Colony on PDA, above (w) and below (x). Scale bars: f−h = 40 µm, i = 50 µm, j = 20 µm, k−v = 10 µm.

***Memnoniella guttulatispora*** H.Z. Du & Jian K. Liu, sp. nov. Figure 13

MycoBank number: MB 854187.

Etymology: The epithet “*guttulatispora*” refers to the guttulate conidia.

**Description**: *Saprobic* on dead petioles of *Alsophila spinulosa* (Cyatheaceae). **Sexual morph**: Undetermined. **Asexual morph**: Hyphomycetous. *Colonies* on natural substrate effuse, glistening, black, single or clustered, globose to subglobose at the apex. *Mycelium* immersed, partly superficial. *Conidiophores* macronematous, mononematous, erect, straight to slightly flexuous, unbranched, smooth, thick-walled, 2–4-septate, hyaline to subhyaline, 115–240 µm ($\overset{ˉ}{x}$ = 161 µm, *n* = 30) long, 10–14 µm ($\overset{ˉ}{x}$ = 12 µm, *n* = 30) wide at the base, tapering to 4–6.5 µm ($\overset{ˉ}{x}$ = 5 µm, *n* = 30) wide at the narrowest point near the apex, bearing 4–6 conidiogenous cells at its apex. *Conidiogenous cells* ellipsoidal, obovate, clavate, monophialidic, discrete, determinate, terminal, smooth, clustered at the apex of conidiophores, hyaline to subhyaline, 11–15 µm ($\overset{ˉ}{x}$ = 13 µm, *n* = 50) long, 6–8 µm ($\overset{ˉ}{x}$ = 7 μm, *n* = 50) wide at the widest part. *Conidia* aggregated in black and glistening heads, ellipsoidal, acrogenous, aseptate, verrucose, with 1–2 large globose guttules in each cell, thick-walled, rounded at both ends, olive grey or olivaceous to brown when young, dark brown to black when mature, 9–13 × 5–7 µm ($\overset{ˉ}{x}$ = 11 × 6 µm, *n* = 50).

**Culture characteristics**: Conidia germinated on PDA within 24 h, and germ tubes produced from basal cells. Colonies growing on PDA reached 16−18 mm in diameter after one month at 25 °C in the dark. Colonies from above, white in the whole colony, flat, circular with filamentous edge. In reverse, brown in the centre, brownish white ring at the margin, light brown pigmentation on PDA.

**Material examined**: CHINA, Sichuan Province, Chengdu City, Jinniu District, Chengdu Botanical Garden, 104°7′19′′E, 30°45′59′′N, elevation 545 m, on dead petioles of medicinal plant *Alsophila spinulosa* (Cyatheaceae), 28 March 2021, H.Z. Du, S189 (HKAS 131298, holotype), ex-type living culture UESTCC 23.0164.

*Notes*: Multi-locus phylogeny showed that *Memnoniella guttulatispora* formed a distinct lineage and was sister to *M. alishanensis* (Figure 5). Morphologically, *M. guttulatispora* is distinguished from *M. alishanensis* by larger conidiophores (115−240 × 10–14 µm vs. 100−150 × 3–5 µm) and ellipsoidal conidia, whereas *M. alishanensis* has reniform or comma-shaped conidia (Tennakoon et al. 2021). Nucleotides comparison of *tub2* sequences showed 96.6% (287/297 bp) similarity between *M. guttulatispora* (UESTCC 23.0164, ex-type isolate) and *M*. *alishanensis* (MFLUCC 20-0168, ex-type isolate). Based on the sequence data recommended by Jeewon and Hyde (2016), *M. guttulatispora* associated with *Alsophila spinulosa*, is introduced as a new species based on morphology and phylogeny from China.

***Memnoniella pseudonilagirica*** L. Lombard & Crous, Persoonia 36: 199 (2016) Figure 14

**Figure 14.**
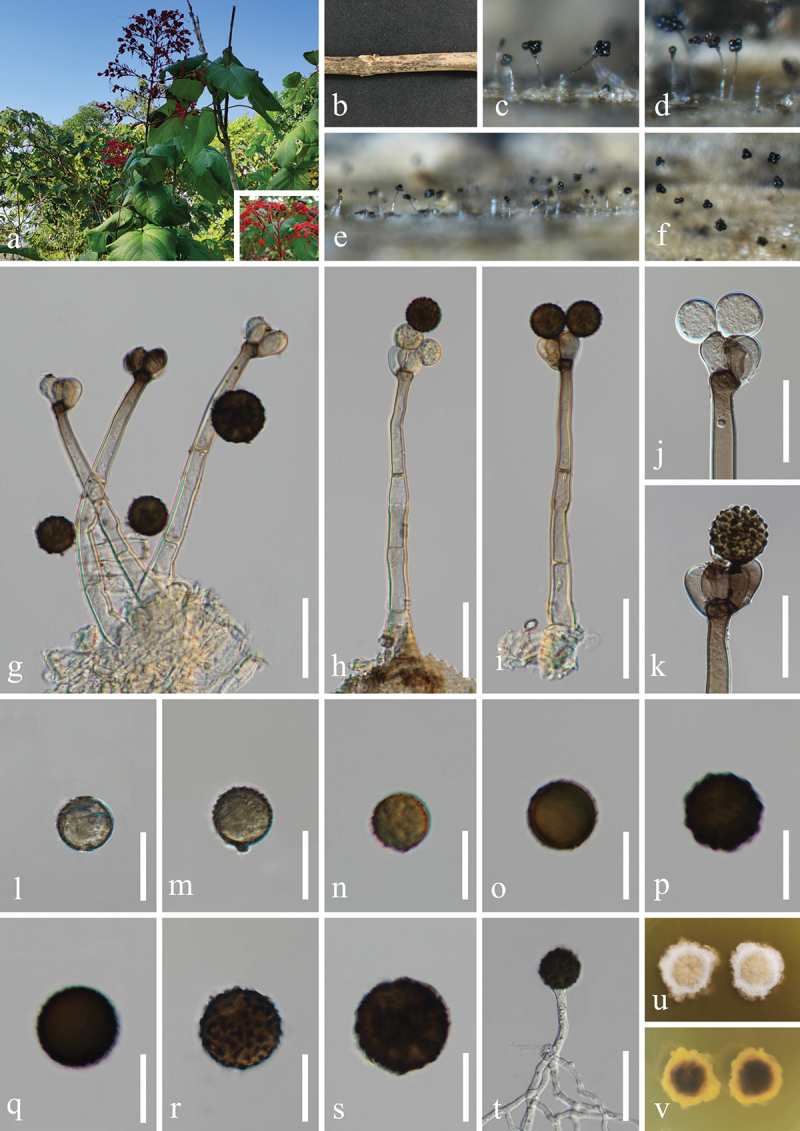
*Memnoniella pseudonilagirica* (HUEST 23.0171, new host and geographical records). (a) Host *Clerodendrum japonicum*. (b) Twig of *Clerodendrum japonicum*. (c−f) Sporulation on the host substrate. (g−i) Conidiophores and conidia. (j, k) Conidiogenous cells and conidia. (l−s) Conidia. (t) Germinated conidium. (u, v) Colonies on PDA, above (u) and below (v). Scale bars: g−i, t = 40 µm, j, k = 30 µm, l−s =20 µm.

= *Memnoniella nilagirica* (Subram.) C.G. Lin, Yong Wang bis & K.D. Hyde, Mycosphere 7(9): 1281 (2016).

**Description**: *Saprobic* on dead twigs of *Clerodendrum japonicum* (Lamiaceae). **Sexual morph**: Undetermined. **Asexual morph**: Hyphomycetous. *Colonies* on the substrate effuse, glistening, black, gregarious, scattered, single or clustered, superficial, bouquet-like at the apex. *Conidiophores* macronematous, mononematous, single, erect, straight to slightly flexuous, thick-walled, unbranched, smooth, 2–4-septate, bearing 3–6 conidiogenous cells at its apex, hyaline to subhyaline, becoming pale olivaceous brown at the apex, 113–162 µm ($\overset{ˉ}{x}$ = 140 µm, *n* = 30) long, 10–17.5 µm ($\overset{ˉ}{x}$ = 12 µm, *n* = 30) wide at the base, tapering to 5–7 µm ($\overset{ˉ}{x}$ = 6 µm, *n* = 30) wide at the narrowest point near the apex. *Conidiogenous cells* clavate, obovate, monophialidic, discrete, with conspicuous collarettes, terminal, clustered at the apex of conidiophores, smooth, hyaline to olivaceous brown, 11–17 µm ($\overset{ˉ}{x}$ = 14 µm, *n* = 50) long, 9–12 µm ($\overset{ˉ}{x}$ = 10 μm, *n* = 50) wide at the widest part. *Conidia* aggregated in black and glistening heads, acrogenous, aseptate, globose, verrucose, thick-walled, olivaceous to olivaceous brown when young, brown to dark brown when mature, 16–27 × 17–27 µm ($\overset{ˉ}{x}$ = 21 × 20 μm, *n* = 50).

**Culture characteristics**: Conidia germinated on PDA within 24 h, and germ tubes produced from basal cells. Colonies growing on PDA reached 15–17 mm in diameter after one month at 25 °C in the dark. Colonies from above, circular, flat, light brown in the middle, white outer ring with irregular edge, filamentous. In reverse, dark brown in the centre, light brown at the margin, bright yellow pigmentation on PDA.

**Material examined**: CHINA, Yunnan Province, Xishuangbanna Dai Autonomous Prefecture, Xishuangbanna Tropical Botanical Garden Chinese Academy of Sciences, 101°15′16′′E, 21°55′51′′N, elevation 505 m, H.Z. Du, 9 November 2022, on dead twigs of medicinal plant *Clerodendrum japonicum* (Lamiaceae), S618 (HUEST 23.0171), living culture UESTCC 23.0171; *ibid*., 10 November 2022, on dead twigs of medicinal plant *Justicia adhatoda* (Acanthaceae), S653 (HUEST 23.0172), living culture UESTCC 23.0172; *ibid*., 8 November 2022, on dead twigs of medicinal plant *Sauropus androgynus* (Euphorbiaceae), S594 (HUEST 23.0173), living culture CGMCC 3.25616; *ibid*., Xishuangbanna Tropical Rain Forest South Medicine Garden, 100°47′17′′E, 22°0′24′′N, elevation 519 m, H.Z. Du, 8 November 2022, on dead twigs of medicinal plant *Scaphium wallichii* (Malvaceae), S553 (HKAS 131304), living culture CGMCC 3.25617.

*Notes*: *Memnoniella pseudonilagirica* was introduced by Lombard et al. (2016) and isolated from dead leaves of *Ceiba pentandra* (L.) Gaertn. in Nepal (Lombard et al. 2016). Subsequently, Lin et al. (2016) transferred *Stachybotrys nilagirica* Subram. to *Memnoniella* as *M. nilagirica* and referred it can be distinguished from *M. pseudonilagirica* by its larger conidiophores and smaller conidia. However, the measurement differences are not significant with 185–350 × 9–22 μm vs. 150–300 × 9–15 μm for conidiophores, and 18–23 × 18–23 μm vs. 18–22 × 17–21 μm for conidia (Lin et al. 2016; Lombard et al. 2016). Comparisons of the ex-type isolates between *M. nilagirica* (MFLUCC 15-0660) and *M. pseudonilagirica* (CBS 136405) showed 99.6% (563/565 bp) in ITS, 99.6% (718/721 bp) in *rpb2*, and 98.8% (635/643 bp) in *cmdA* sequence similarity (Lin et al. 2016; Lombard et al. 2016). In this study, we reported four additional isolates of *M. pseudonilagirica* from China, these isolates and *M. nilagirica* (MFLUCC 15-0660) grouped within *M. pseudonilagirica* subclade in phylogenetic analyses (Figure 5). Therefore, we synonymised *M. nilagirica* under *M. pseudonilagirica*, and our new isolates are regarded as the new host and geographical records of *M. pseudonilagirica*, isolated from medicinal plants of *Clerodendrum japonicum*, *Justicia adhatoda*, *Sauropus androgynus*, and *Scaphium wallichii* in China.

**Figure 15.**
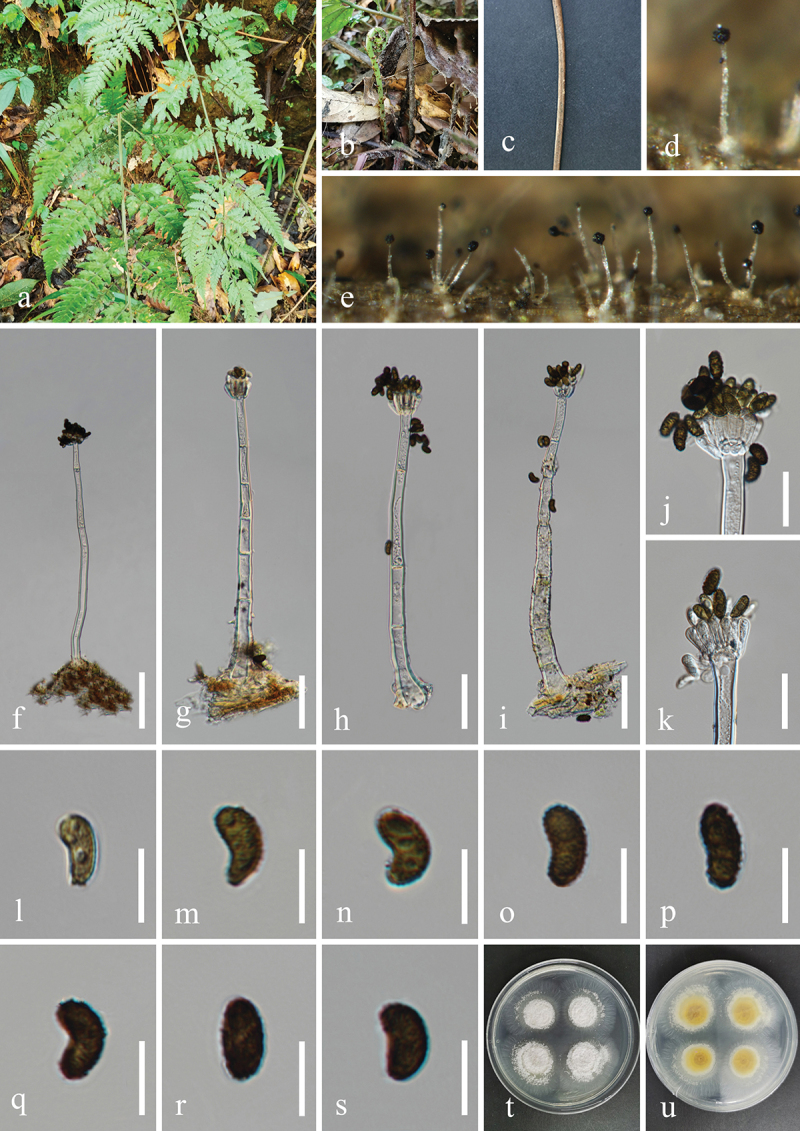
*Memnoniella reniformis* (HKAS 131303, holotype). (a) Host *Dryopteris* sp. (b, c) Petioles of *Dryopteris* sp., raw (b) and dead (c). (d, e) Sporulation on the host substrate. (f−i) Conidiophores and conidia. (j, k) Conidiogenous cells and conidia. (l−s) Conidia. (t, u) Colonies on PDA, above (t) and below (u). Scale bars: f−i = 40 µm, j, k = 20 µm, l−s = 10 µm.

***Memnoniella reniformis*** H.Z. Du & Jian K. Liu, sp. nov. Figure 15

MycoBank number: MB 854198.

Etymology: The epithet “*reniformis*” refers to the reniform shape of conidia.

**Description**: *Saprobic* on dead petioles of *Dryopteris* sp. (Dryopteridaceae). **Sexual morph**: Undetermined. **Asexual morph**: Hyphomycetous. *Colonies* on natural substrate effuse, glistening, black, single or clustered, globose at the apex. *Mycelium* immersed, partly superficial. *Conidiophores* macronematous, mononematous, single, erect, straight to slightly flexuous, unbranched, smooth or slightly verrucose, thick-walled, 2–6-septate, hyaline to subhyaline,164–255 µm ($\overset{ˉ}{x}$ = 217 µm, *n* = 30) long, 7–16 µm ($\overset{ˉ}{x}$ = 13 µm, *n* = 30) wide at the base, tapering to 4–9 µm ($\overset{ˉ}{x}$ = 6 µm, *n* = 30) wide at the narrowest point near the apex, bearing 4–8 conidiogenous cells at its apex. *Conidiogenous cells* clavate, obovate, monophialidic, discrete indistinctively, terminal, clustered at the apex of conidiophores, unsmooth, hyaline to subhyaline, 10–14 µm ($\overset{ˉ}{x}$ = 11.5 µm, *n* = 50) long, 3–5 µm ($\overset{ˉ}{x}$ = 4 μm, *n* = 50) wide at the widest part. *Conidia* aggregated in black and glistening heads, ovoid, reniform, or comma shaped, some are curved into bean shape, acrogenous, aseptate, verrucose, mostly 2 guttules, rarely 3–4 unequal guttules, thick-walled, rounded at both ends, olivaceous brown to dark brown, 9.5–12 × 5–6 µm ($\overset{ˉ}{x}$ = 11 × 5.5 μm, *n* = 50).

**Culture characteristics**: Conidia germinated on PDA within 12 h, and germ tubes produced from basal cells. Colonies growing on PDA reached 15–18 mm in diameter after three weeks at 25 °C in the dark. Colonies from above, white in the whole colony, flat, surface smooth, sparse hyaline mycelium on the outer ring with filamentous edge. In reverse, bright yellow in the centre, hyaline to white at the margin.

**Material examined**: CHINA, Sichuan Province, Chengdu City, Dujiangyan City, Qingcheng Mountain scenic spot, 103°28′36′′E, 30°55′9′′N, elevation 1,178 m, on dead petioles of medicinal plant *Dryopteris* sp. (Dryopteridaceae), 27 March 2021, H.Z. Du, S179 (HKAS 131303, holotype), ex-type living culture UESTCC 23.0170.

*Notes*: The sterile isolate of *Memnoniella* sp. (MUCL 50191), represented a single lineage, was reported by Lombard et al. (2016), and its novelty was not confirmed due to the lack of morphological descriptions. In this study, *M. reniformis* (UESTCC 23.0170, ex-type isolate) was sister to *Memnoniella* sp. (MUCL 50191) and can be recognised as a distinct phylogenetic species (Figure 5). Additionally, the nucleotide base pair comparison between UESTCC 23.0170 and MUCL 50191 revealed the sequence similarities of 86.1% (460/534 bp) in *cmdA*, 97.2% (524/539 bp) in ITS, 90.3% (649/719 bp) in *rpb2*, 85.9% (329/383 bp) in *tef1-α*, and 86.3% (258/299 bp) in *tub2*. Therefore, we identified our isolate as a new species, *M. reniformis*.

**Figure 16.**
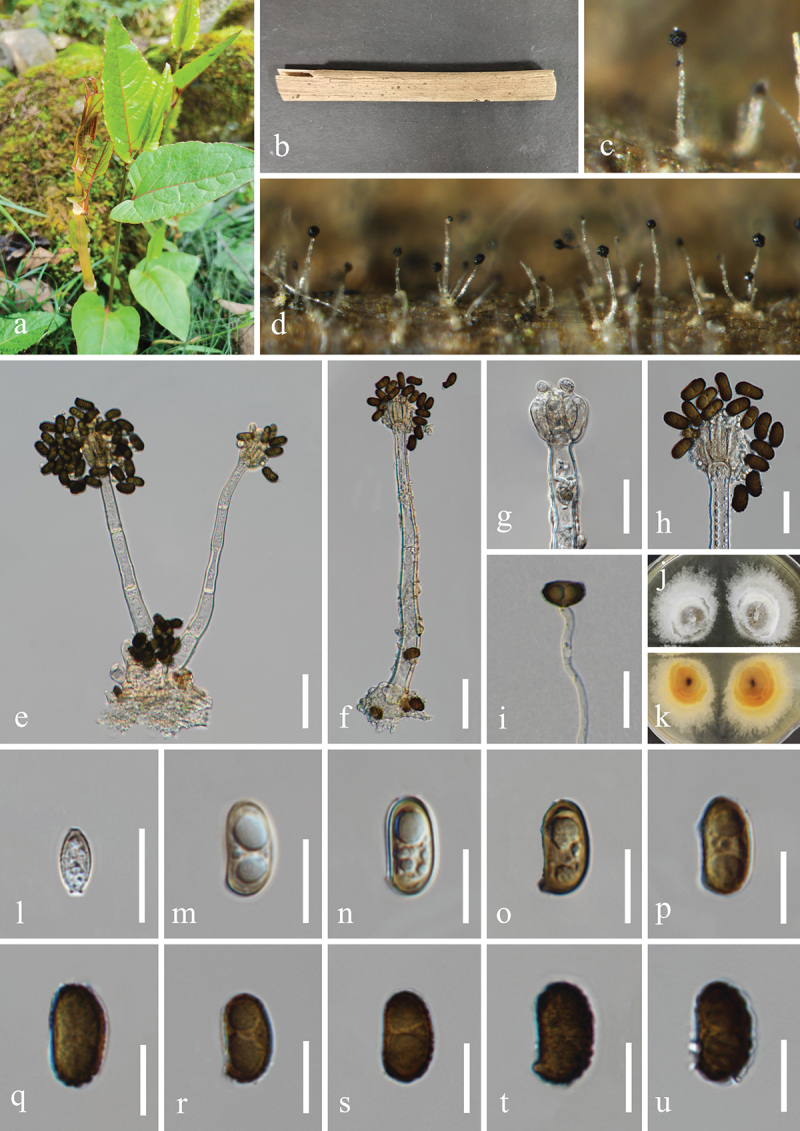
*Memnoniella reynoutriae* (HKAS 131302, holotype). (a) Host *Reynoutria japonica*. (b) Stem of *Reynoutria japonica*. (c, d) Sporulation on the host substrate. (e, f) Conidiophores and conidia. (g, h) Conidiogenous cells and conidia. (i) Germinated conidium. (j, k) Colonies on PDA, above (j) and below (k). (l−u) Conidia. Scale bars: e, f = 40 µm, g−i = 20 µm, l = 15 µm, m−u = 10 µm.

***Memnoniella reynoutriae*** H.Z. Du & Jian K. Liu, sp. nov. Figure 16

MycoBank number: MB 854197.

Etymology: The epithet “*reynoutriae*” refers to the host genus *Reynoutria* from which the fungus was originally isolated.

**Description**: *Saprobic* on dead stems of *Reynoutria japonica* (Polygonaceae). **Sexual morph**: Undetermined. **Asexual morph**: Hyphomycetous. *Colonies* on natural substrate effuse, glistening, black, single or clustered, globose at the apex. *Mycelium* immersed, partly superficial. *Conidiophores* macronematous, mononematous, simple, erect, straight or flexuous, smooth or with sparse and scattered ornament, thick-walled, unbranched, hyaline to subhyaline, 2–5-septate, 136–246 µm ($\overset{ˉ}{x}$ = 189 µm, *n* = 30) long, 12–20 µm ($\overset{ˉ}{x}$ = 16 µm, *n* = 30) wide at the base, tapering to 6–9 µm ($\overset{ˉ}{x}$ = 8 µm, *n* = 30) wide at the narrowest point near the apex, bearing 3–6 conidiogenous cells at its apex. *Conidiogenous cells* clavate, obovate, monophialidic, discrete indistinctively, terminal, unsmooth with irregular tiny verrucae, subhyaline to olivaceous, 15–17 µm ($\overset{ˉ}{x}$ = 15.5 µm, *n* = 50) long, 5–6 µm ($\overset{ˉ}{x}$ = 6 μm, *n* = 50) wide at the widest part. *Conidia* aggregated in black and glistening heads, ellipsoidal, acrogenous, aseptate, verrucose, with 2 large globose guttules, rarely 1 guttule, thick-walled, rounded at both ends or slightly narrowing to a central or oblique base, subhyaline to olivaceous when young, brown to dark brown when mature, 12–16 × 6.5–9 µm ($\overset{ˉ}{x}$ = 14 × 8 µm, *n* = 50).

**Culture characteristics**: Conidia germinated on PDA within 12 h, and germ tubes produced from basal cells. Colonies growing on PDA reached 42–43 mm in diameter after three weeks at 25 °C in the dark. Colonies from above, white in the whole colony, flat, surface smooth, sparse white mycelium on the outer ring with filamentous edge. In reverse, dark brown at the central point, brown in the centre, light brown at the margin, light brown pigmentation on PDA.

**Material examined**: CHINA, Sichuan Province, Chengdu City, Dujiangyan City, Qingcheng Mountain scenic spot, 103°28′36′′E, 30°55′9′′N, elevation 1,178 m, on dead stems of medicinal plant *Reynoutria japonica* (Polygonaceae), 27 March 2021, H.Z. Du, S160 (HKAS 131302, holotype; HUEST 23.0169, isotype), ex-type living culture CGMCC 3.25615; ex-isotype living culture UESTCC 23.0169.

*Notes*: *Memnoniella reynoutriae* shares similar morphology with *M. pseudodichroa* (Yeh et al. 2023) in having macronematous and mononematous conidiophores with ellipsoidal to cylindrical conidiogenous cells at apex, and acrogenous, ellipsoidal and verrucose conidia with two guttules. However, the conidiogenous cells of *M. reynoutriae* are unsmooth with irregular tiny verrucae, while *M. pseudodichroa* is smooth, rarely sparsely rough. The phylogenetic result (Figure 5) showed that *M. reynoutriae* (CGMCC 3.25615 and UESTCC 23.0169) can be recognised as a distinct phylogenetic species with high bootstrap support (100% MLBS/1.00 BIPP). In addition, comparisons the base pair of ex-type isolates between *M. reynoutriae* R. Kirschner (CGMCC 3.25615) and *M. pseudodichroa* (BCRC-FU 31689) revealed 98.1% (529/539 bp) in ITS, 97.0% (781/805 bp) in *rpb2*, 93.9% (400/426 bp) in *tef1-α*, and 96.0% (241/251 bp) in *tub2* similarity. Therefore, *M. reynoutriae* associated with *Reynoutria japonica* is introduced as a new species in China.

**Figure 17.**
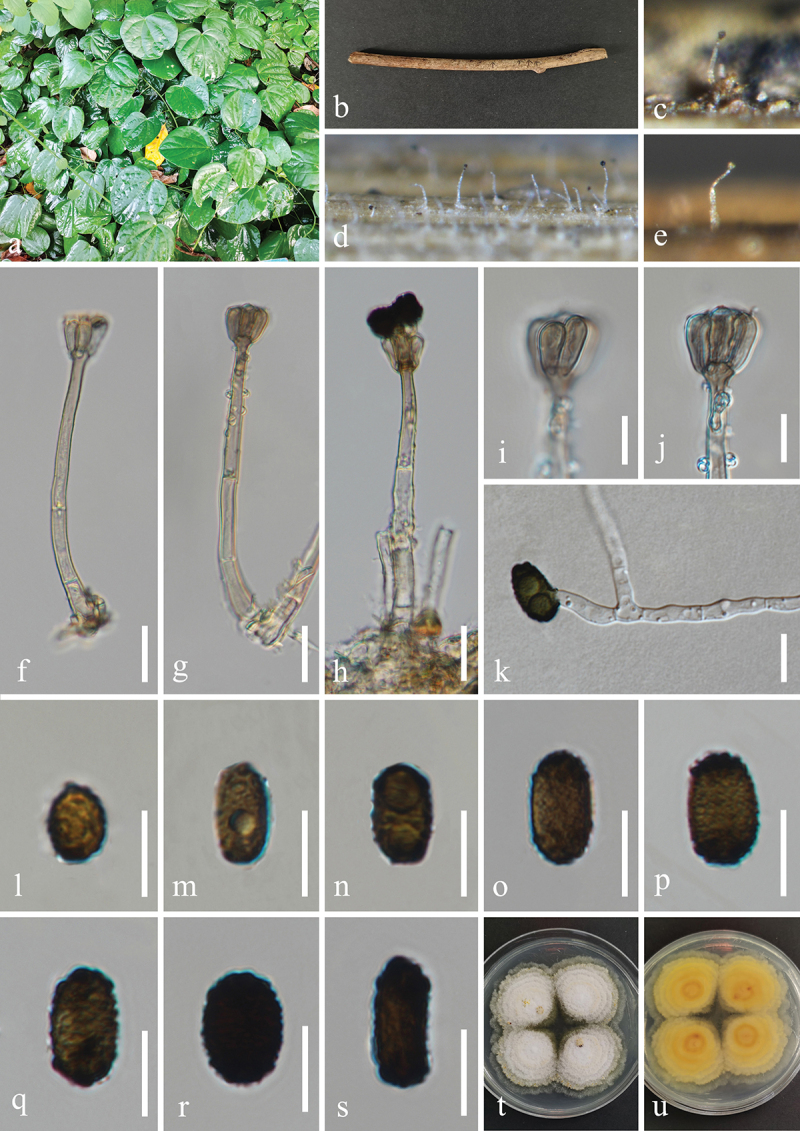
*Memnoniella verrucosispora* (HKAS 131301, holotype). (a) Host *Fibraurea recisa*. (b) Twig of *Fibraurea recisa*. (c−e) Sporulation on the host substrate. (f−h) Conidiophores and conidia. (i, j) Conidiogenous cells. (k) Germinated conidium. (l−s) Conidia. (t, u) Colonies on PDA, above (t) and below (u). Scale bars: f−h = 20 µm, i−s = 10 µm.

***Memnoniella verrucosispora*** H.Z. Du & Jian K. Liu, sp. nov. Figure 17

MycoBank number: MB 854196.

Etymology: The epithet “*verrucosispora*” refers to the verrucose surface of conidia.

**Description**: *Saprobic* on dead twigs of *Fibraurea recisa* (Menispermaceae). **Sexual morph**: Undetermined. **Asexual morph**: Hyphomycetous. *Colonies* on natural substrate effuse, single or clustered, hyaline or black at the apex. *Mycelium* immersed, partly superficial. *Conidiophores* macronematous, mononematous, simple, erect, straight to flexuous, smooth or slightly verrucose, thick-walled, unbranched, 2–3-septate, hyaline or subhyaline to light brown towards the apex, 104–150 µm ($\overset{ˉ}{x}$ = 123 µm, *n* = 30) long, 7–12 µm ($\overset{ˉ}{x}$ = 10 µm, *n* = 30) wide at the base, tapering to 4–8 µm ($\overset{ˉ}{x}$ = 5.5 µm, *n* = 30) wide at the narrowest point near the apex, bearing 4–6 conidiogenous cells at its apex. *Conidiogenous cells* clavate, obovate, monophialidic, discrete, determinate, terminal, smooth, subhyaline to olivaceous or olivaceous brown, 11–14 µm ($\overset{ˉ}{x}$ = 12 µm, *n* = 50) long, 5–7 µm ($\overset{ˉ}{x}$ = 6 μm, *n* = 50) wide at the widest part. *Conidia* aggregated in black heads, ellipsoidal, acrogenous, aseptate, rarely oblong to short-cylindrical, straight or slightly curved by one side, unsmooth, distinctly verrucose, with 1–2 guttules, thick-walled, olivaceous brown to brown when young, dark brown to black when mature, (8.5–)11–15 × 6–9 µm ($\overset{ˉ}{x}$ = 12 × 7.5 μm, *n* = 50).

**Culture characteristics**: Conidia germinated on PDA within 24 h, and germ tubes produced from basal cells. Colonies growing on PDA reached 33–35 mm in diameter after three weeks at 25 °C in the dark. Colonies from above, white in the whole colony, filamentous edge, irregular, flat, surface smooth, formed multiple concentric rings from centre to edge. In reverse, brown ring in the middle, and brown to light brown rings from centre to edge, light yellow pigmentation on PDA.

**Material examined**: CHINA, Yunnan Province, Xishuangbanna Dai Autonomous Prefecture, Mengla County, Xishuangbanna Tropical Botanical Garden Chinese Academy of Sciences. 101°15′6′′E, 21°55′51′′N, elevation 502 m, on dead twigs of medicinal plant *Fibraurea recisa* Pierre (Menispermaceae), 9 November 2022, H.Z. Du, S604 (HKAS 131301, holotype; HUEST 23.0168, isotype), ex-type living culture CGMCC 3.25614; ex-isotype living culture UESTCC 23.0168.

*Notes*: *Memnoniella verrucosispora* (CGMCC 3.25614 and UESTCC 23.0168) formed a basal lineage to *M. mori* and *M. cnidiicola* with significant statistical support (99% MLBS/1.00 BIPP) (Figure 5). *Memnoniella verrucosispora* (CGMCC 3.25614, ex-type isolate) can be distinguished from *M. mori* (MFLUCC 18-1640, ex-type isolate) by 462/524 bp (88.2%) in ITS and 278/293 bp (94.9%) in *tub2*, and differs from *M. cnidiicola* (CGMCC 3.25686, ex-type isolate) by 97.9% (645/659 bp) of *cmdA*, 88.9% (457/514 bp) of ITS, 97.6% (403/413 bp) of *tef1-α*, and 96.6% (282/292 bp) of *tub2* similarity. Morphologically, the conidiophores of *M. verrucosispora* (104–150 × 7–12 µm) are smaller than *M. cnidiicola* (180−236 × 11–16 µm) and larger than *M. mori* (70−110 × 4–6.5 µm) (Tennakoon et al. 2021). Therefore, *M. verrucosispora*, isolated from the medicinal plant of *Fibraurea recisa* in China, is introduced as a new species based on morphological and phylogenetic evidence.

***Sirastachys*** L. Lombard & Crous, Persoonia 36: 215 (2016)

*Notes*: The asexual morpho of *Sirastachys* was introduced by Lombard et al. (2016) based on multi-locus phylogenetic analyses (ITS, LSU, *rpb2*, *cmdA*, *tef1-α*, and *tub2*), with *Sir. phaeospora* as the type species. This genus is characterised by synnemata formation in culture, lateral conidiophores, phialidic conidiogenous cells, and ellipsoidal to cylindrical conidia (Lombard et al. 2016). Presently, nine species of *Sirastachys* were recorded in the Species Fungorum (https://www.speciesfungorum.org/Names/Names.asp?strGenus=*Sirastachys*; accessed on 9 December 2024).

**Figure 18.**
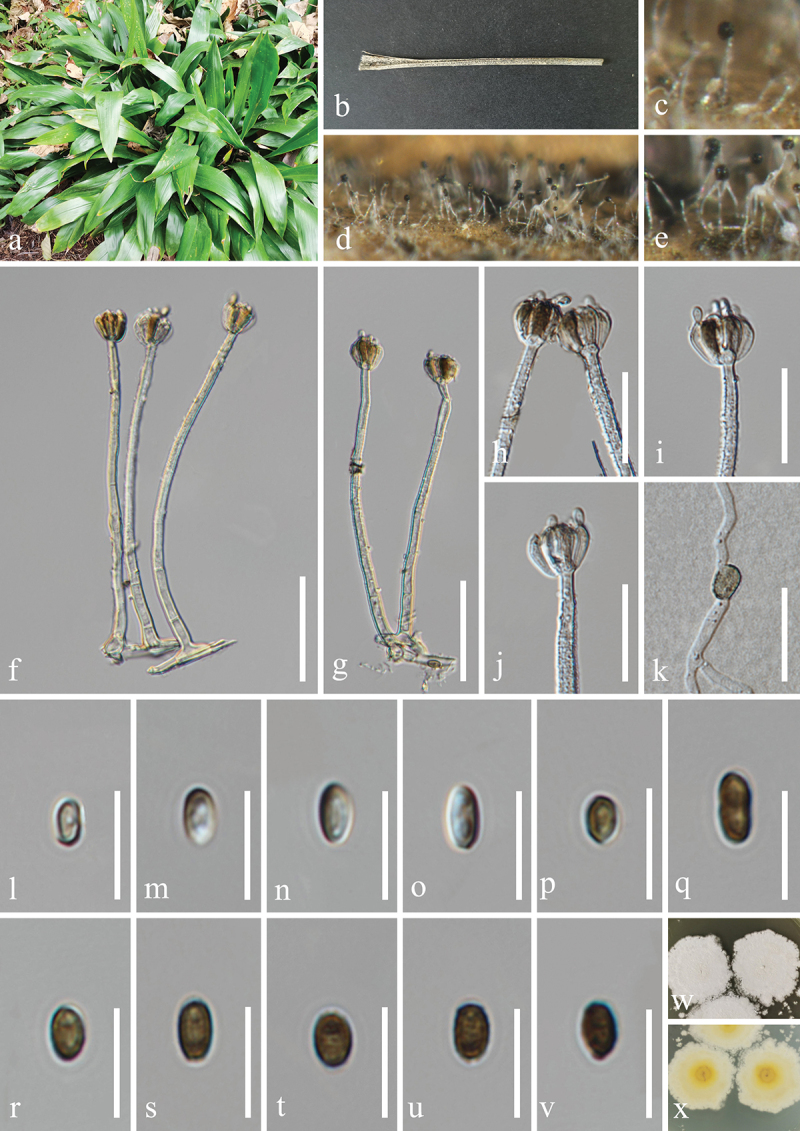
*Sirastachys aspidistrae* (HKAS 131308, holotype). (a) Host *Aspidistra elatior*. (b) Petiole of *Aspidistra elatior*. (c−e) Sporulation on the host substrate. (f, g) Conidiophores and conidia. (h−j) Conidiogenous cells and conidia. (k) Germinated conidium. (l−v) Conidia. (w, x) Colonies on PDA, above (w) and below (x). Scale bars: f, g = 40 µm, h−k = 20 µm, l−v = 10 µm.

***Sirastachys*** ***aspidistrae*** H.Z. Du & Jian K. Liu, sp. nov. Figure 18

MycoBank number: MB 854200.

Etymology: The epithet “*aspidistrae*” refers to the host genus *Aspidistra* from which the fungus was originally isolated.

**Description**: *Saprobic* on dead petioles of *Aspidistra elatior* (Asparagaceae). **Sexual morph**: Undetermined. **Asexual morph**: Hyphomycetous. *Colonies* on natural substrate effuse, glistening, black, clustered a globose nucleus at the apex. *Mycelium* immersed, partly superficial. *Conidiophores* macronematous, mononematous, single or in groups, erect, straight to flexuous, unbranched or multibranched, 2–4(−6)-septate, thin-walled, unsmooth with verrucose, hyaline to subhyaline, 57–193 µm ($\overset{ˉ}{x}$ = 110 µm, *n* = 20) long, 5–7 µm ($\overset{ˉ}{x}$ = 6 µm, *n* = 20) wide at the base, tapering to 2.5–4 µm ($\overset{ˉ}{x}$ = 3 µm, *n* = 20) wide at the narrowest point near the apex, bearing a whorl of 4–8 conidiogenous cells. *Conidiogenous cells* phialidic, terminal, elongate doliiform to clavate, smooth to slightly verrucose, longitudinally striate, with conspicuous collarettes and periclinal thickenings, not obviously discrete, clustered at the apex of conidiophores, subhyaline to light brown, 7–10 µm ($\overset{ˉ}{x}$ = 9 µm, *n* = 50) long, 3–5 µm ($\overset{ˉ}{x}$ = 4 μm, *n* = 50) wide at the widest part. *Conidia* acrogenous, aseptate, obovoid to ellipsoidal, mostly 2 guttules, thick-walled, rounded at both ends. Olivaceous when young, brown to dark brown when mature, 4–6 × 3–4 µm ($\overset{ˉ}{x}$ = 5 × 3 μm, *n* = 50).

**Culture characteristics**: Conidia germinated on PDA within 24 h, and germ tubes produced from basal cells. Colonies growing on PDA reached 25–27 mm in diameter after two weeks at 25 °C in the dark. Colonies from above, white in the whole colony, surface smooth, erose edge, irregular. In reverse, white to light yellow at the margin, light yellow to yellow in the central rings, the colour gradually lightens from centre to edge, light yellow pigmentation on PDA.

**Material examined**: CHINA, Sichuan Province, Chengdu City, High-tech West District, Yaobo Park, 103°56′21′′E, 30°43′57′′N, elevation 504 m, on dead petioles of medicinal plant *Aspidistra elatior* (Asparagaceae), 11 August 2021, H.Z. Du, S336 (HKAS 131308, holotype; HUEST 23.0182, isotype); ex-type living culture CGMCC 3.25687; ex-isotype living culture UESTCC 23.0182.

*Notes*: *Sirastachys aspidistrae* morphologically resembles *Sir. phaeospora* (Lombard et al. 2016) by having macronematous, mononematous conidiophores bearing 4–8 conidiogenous cells at the apex and acrogenous, obovoid to ellipsoidal conidia. However, *Sir. aspidistrae* differs from *Sir. phaeospora* in having larger conidiophores (57–193 × 5–7 µm vs. 40−65 × 3–5 µm). Phylogenetically, the two isolates (CGMCC 3.25687 and UESTCC 23.0182) of *Sir. aspidistrae* clustered together with strong support (100% MLBS/1.00 BIPP) and sister to *Sir. phaeospora* (CBS 100155, ex-type isolate) with 95% MLBS, 1.00 BIPP statistical support (Figure 5). Comparisons of the *cmdA*, ITS, *tef1-α*, and *tub2* loci of ex-type isolates between *Sir. aspidistrae* (CGMCC 3.25687) and *Sir. phaeospora* (CBS 100155) revealed 97.1% (636/655 bp), 98.4% (546/555 bp), 95.5% (382/400 bp), and 97.9% (285/291 bp) similarity, respectively. Therefore, *Sir. aspidistrae*, associated with *Aspidistra elatior*, is described as a new species in China.

***Sirastachys castanedae*** L. Lombard & Crous, Persoonia 36: 216 (2016) Figure 19

**Figure 19.**
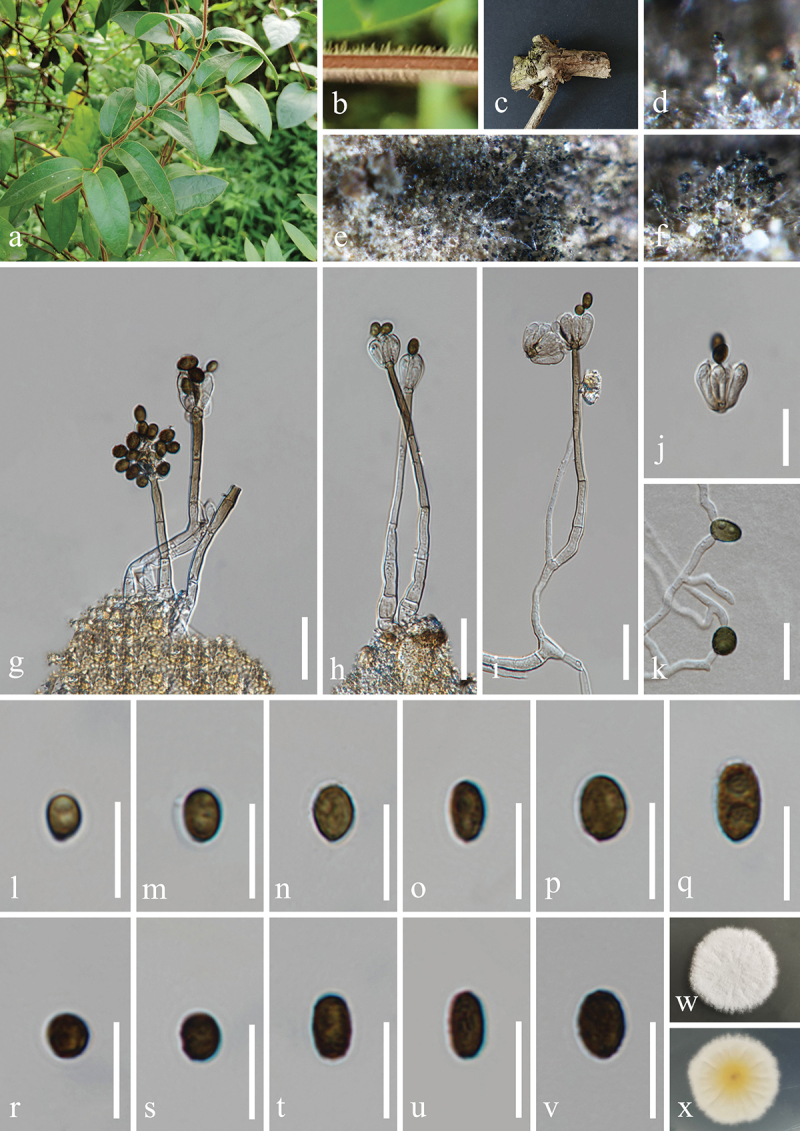
*Sirastachys castanedae* (HUEST 23.0184, new host record). (a) Host *Lonicera guillonii* var. *macranthoides*. (b, c) Twigs of *Lonicera guillonii* var. *macranthoides*, raw (b) and dead (c). (d−f) Sporulation on the host substrate. (g−i) Conidiophores and conidia. (j) Conidiogenous cells and conidia. (k) Germinated conidium. (l−v) Conidia. (w, x) Colony on PDA, above (w) and below (x). Scale bars: g−i = 20 µm, j, k = 15 µm, l−v = 10 µm.

**Description**: *Saprobic* on dead twigs of *Lonicera guillonii* var. *macranthoides* (Caprifoliaceae). **Sexual morph**: Undetermined. **Asexual morph**: Hyphomycetous. *Colonies* on the substrate surface are black and hairy. *Mycelium* immersed, partly superficial. *Conidiophores* macronematous, mononematous, single or in groups, erect, straight to flexuous, unbranched or branched once to twice, smooth, 2–4(−5)-septate, thin-walled, hyaline to subhyaline at base, hyaline to olivaceous brown towards the apex, 66–127 µm ($\overset{ˉ}{x}$ = 91 µm, *n* = 30) long, 3–6 µm ($\overset{ˉ}{x}$ = 5 µm, *n* = 30) wide at the base, tapering to 3–4 µm ($\overset{ˉ}{x}$ = 3.5 µm, *n* = 30) wide at the narrowest point near the apex, bearing a whorl of 4–8 conidiogenous cells. *Conidiogenous cells* phialidic, terminal, elongate doliiform to clavate, clustered at the apex of conidiophores, smooth to slightly verrucose, hyaline to subhyaline, 8.5–13.5 µm ($\overset{ˉ}{x}$ = 11 µm, *n* = 50) long, 3–5 µm ($\overset{ˉ}{x}$ = 4 μm, *n* = 50) wide at the widest part. *Conidia* aggregated in black heads, obovoid to ellipsoidal, acrogenous, aseptate, verrucose, rarely with 1–2 guttules, darkly olivaceous to dark brown, rounded at both ends, 5–8 × 4–5.5 µm ($\overset{ˉ}{x}$ = 6 × 4.5 μm, *n* = 50).

**Culture characteristics**: Conidia germinated on PDA within 48 h, and germ tubes produced from basal cells. Colonies growing on PDA reached 25–27 mm in diameter after one month at 25 °C in the dark. Colonies from above, white in the whole colony, circular with filamentous edge, surface smooth. In reverse, yellow to light yellow in the centre, white at the margin, the colour gradually lightens from centre to edge.

**Material examined**: CHINA, Guizhou Province, Guiyang City, Huaxi District, Guizhou University of Traditional Chinese Medicine, 106°38′13′′E, 26°22′14′′N, elevation 1,140 m, on dead twigs of medicinal plant *Lonicera guillonii* var. *macranthoides* (Caprifoliaceae), 2 June 2021, H.Z. Du, S445 (HUEST 23.0184), living culture UESTCC 23.0184.

*Notes*: *Sirastachys castanedae* was introduced by Lombard et al. (2016). Multi-locus phylogeny revealed that our isolate (UESTCC 23.0184) has a close relationship with the ex-type isolate (CBS 136403) of *Sir. castanedae* with strong support (100% MLBS/1.00 BIPP) (Figure 5), and our specimen (HUEST 23.0184) is morphologically similar to the holotype (CBS H-22457) of *Sir. castanedae* (Lombard et al. 2016). Therefore, we identify the new collection as *Sir. castanedae* based on morphology and phylogeny. This is the first report of *Sir. castanedae* from medicinal plant *Lonicera guillonii* var. *macranthoides* in China.

**Figure 20.**
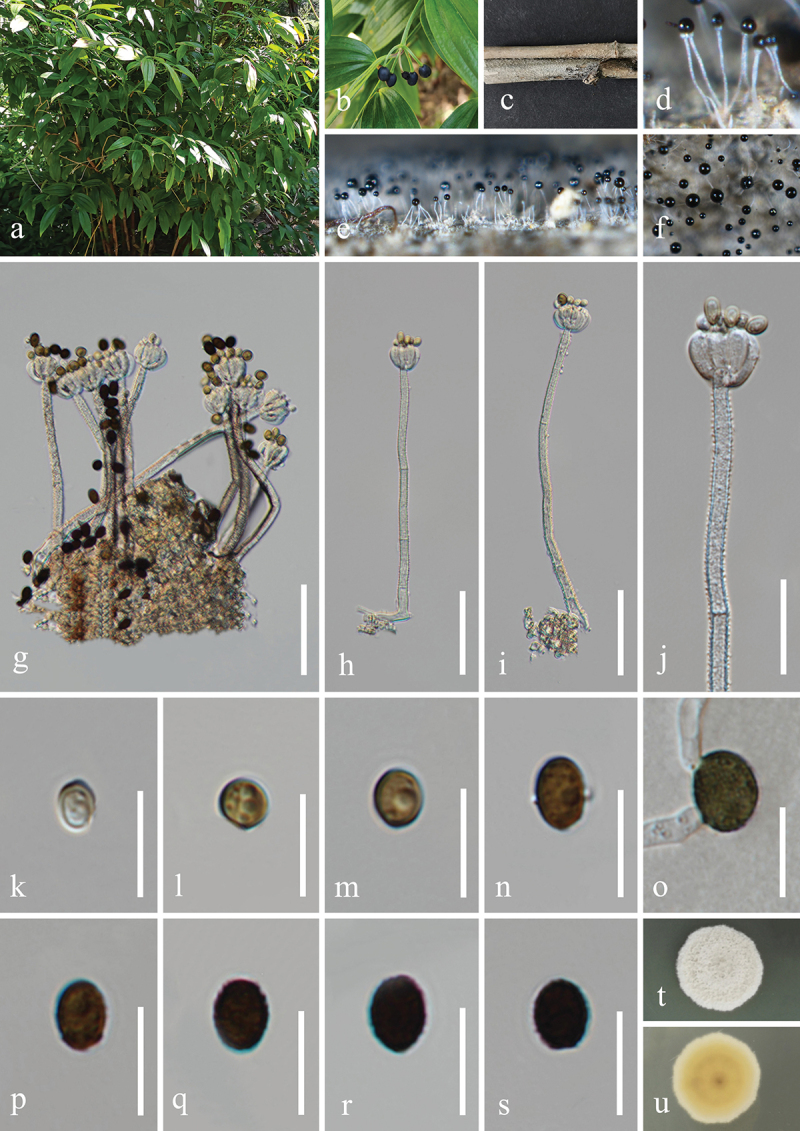
*Sirastachys ellipsoidispora* (HKAS 131309, holotype). (a) Host *Disporum longistylum*. (b) Fruits of *Disporum longistylum*. (c) Twig of *Disporum longistylum*. (d−f) Sporulation on the host substrate. (g−i) Conidiophores and conidia. (j) Conidiogenous cells and conidia. (k−n, p−s) Conidia. (o) Germinated conidium. (t, u) Colony on PDA, above (t) and below (u). Scale bars: g−i = 40 µm, j = 20 µm, k−s = 10 µm.

***Sirastachys*** ***ellipsoidispora*** H.Z. Du & Jian K. Liu, sp. nov. Figure 20

MycoBank number: MB 854201.

**Etymology**: The epithet “*ellipsoidispora*” reflects the ellipsoidal conidia produced by this fungus.

**Description**: *Saprobic* on dead twigs of *Disporum longistylum* (Liliaceae). **Sexual morph**: Undetermined. **Asexual morph**: Hyphomycetous. *Colonies* on natural substrate effuse, glistening, black, single, or clustered a globose nucleus at the apex. *Mycelium* immersed, partly superficial. *Conidiophores* macronematous, mononematous, single or in groups, erect, straight to flexuous, unbranched or multibranched, 2–4(−6)-septate, thin-walled, spinulose, hyaline to subhyaline, 124–183 µm ($\overset{ˉ}{x}$ = 150 µm, *n* = 30) long, 5–6.5 µm ($\overset{ˉ}{x}$ = 5.5 µm, *n* = 30) wide at the base, tapering to 3–5.5 µm ($\overset{ˉ}{x}$ = 4 µm, *n* = 30) wide at the narrowest point near the apex, bearing a whorl of 4–8 conidiogenous cells. *Conidiogenous cells* phialidic, terminal, elongate doliiform to clavate, with conspicuous collarettes and periclinal thickenings, not obviously discrete, smooth to slightly verrucose, clustered at the apex of conidiophores, hyaline to subhyaline, 7.5–12 µm ($\overset{ˉ}{x}$ = 10 µm, *n* = 50) long, 4–5 µm ($\overset{ˉ}{x}$ = 4 μm, *n* = 50) wide at the widest part. *Conidia* aggregated in black and glistening heads, obovoid to ellipsoidal, acrogenous, aseptate, verrucose, mostly 1 guttule, rarely 3 guttules, rounded at both ends, olivaceous to brown when young, dark brown to black when mature, 5–8 × 3–6 µm ($\overset{ˉ}{x}$ = 7 × 5 μm, *n* = 50).

**Culture characteristics**: Conidia germinated on PDA within 24 h, and germ tubes produced from basal cells. Colonies growing on PDA reached 25–27 mm in diameter after two weeks at 25 °C in the dark. Colonies from above, white in the whole colony, circular with entire edge, surface smooth. In reverse, light brown in the centre, white to light brown at the margin, the colour gradually lightens from centre to edge.

**Material examined**: CHINA, Yunnan Province, Kunming City, Panlong District, Kunming Botanical Garden, 102°44′24′′E, 25°8′27′′N, elevation 1,921 m, on dead twigs of medicinal plant *Disporum longistylum* (Liliaceae), 11 November 2022, H.Z. Du, S752 (HKAS 131309, holotype; HUEST 23.0183, isotype); ex-type living culture CGMCC 3.25621; ex-isotype living culture UESTCC 23.0183.

*Notes*: *Sirastachys ellipsoidispora* (ex-type isolate CGMCC 3.25621 and UESTCC 23.0183) has close phylogenetic relationships with *Sir. phyllophila* (CBS 136169, ex-type isolate) but formed a distinct lineage with significant statistical support (99% MLBS/1.00 BIPP) (Figure 3). Morphologically, *Sir. ellipsoidispora* differs from *Sir. phyllophila* in having spinulose and larger conidiophores (124–183 × 5–6.5 µm vs. 80−150 × 3–5 µm), and circular to ellipsoidal conidia (Lombard et al. 2016). In addition, *Sir. ellipsoidispora* can be distinguished from *Sir. phyllophila* based on *cmdA*, ITS, *rpb2*, *tef1-α*, and *tub2* nucleotide base pair similarity of ex-type isolates; *cmdA* = 91.0% (595/654 bp), ITS = 97.6% (534/547 bp), *rpb2* = 97.0% (893/921 bp), *tef1-α* = 92.4% (437/473 bp), and *tub2* = 97.0% (292/301 bp). Therefore, we established *Sir. ellipsoidispora* as a new species associated with medicinal plant *Disporum longistylum* in China.

***Stachybotrys*** Corda, Icones Fungorum 1: 21 (1837)

*Notes*: *Stachybotrys* was established by Corda (1837) to accommodate *Sta. atrus* Corda (= *Sta. chartarum*) as the type species. *Stachybotrys* is a long-standing genus, shares morphological similarities with *Memnoniella* (Ellis 1971; Jong and Davis 1976; Photita et al. 2003; Pinruan et al. 2004; Manoharachary et al. 2006; Seifert and Gams 2011; Wang et al. 2015; Lombard et al. 2016; Zheng et al. 2019; Samarakoon et al. 2021). Most species of *Stachybotrys* are cellulolytic saprobes (Whitton et al. 2001; Li and Jiang 2011; Samarakoon et al. 2021; Tennakoon et al. 2021), plant pathogens (Pathak and Chauhan 1976; Zhao et al. 2010), and endophytes (Cao et al. 2009; Raghavendra and Newcombe 2012; Busby et al. 2016; Zhang et al. 2019; Yang et al. 2020). They can be found on various hosts in diverse habitats, including dead wood, damp paper, cotton, linen, cellulose-based building materials, water-damaged indoor buildings, and air ducts (Ellis 1971; Tang et al. 2003; Santa Izabel et al. 2010; Wang et al. 2015; Lin et al. 2016; Lombard et al. 2016; Mapook et al. 2020). Currently, 90 species of *Stachybotrys* were accepted in the Species Fungorum (https://www.speciesfungorum.org/Names/Names.asp?strGenus=*Stachybotrys*; accessed on 9 December 2024), but only 39 taxa of this genus have DNA sequence data in GenBank (https://www.ncbi.nlm.nih.gov/nuccore/?term=*Stachybotrys*+; accessed on 9 December 2024).

***Stachybotrys chartarum*** (Ehrenb.) S. Hughes, Canadian Journal of Botany 36: 812 (1958) Figure 21

**Figure 21.**
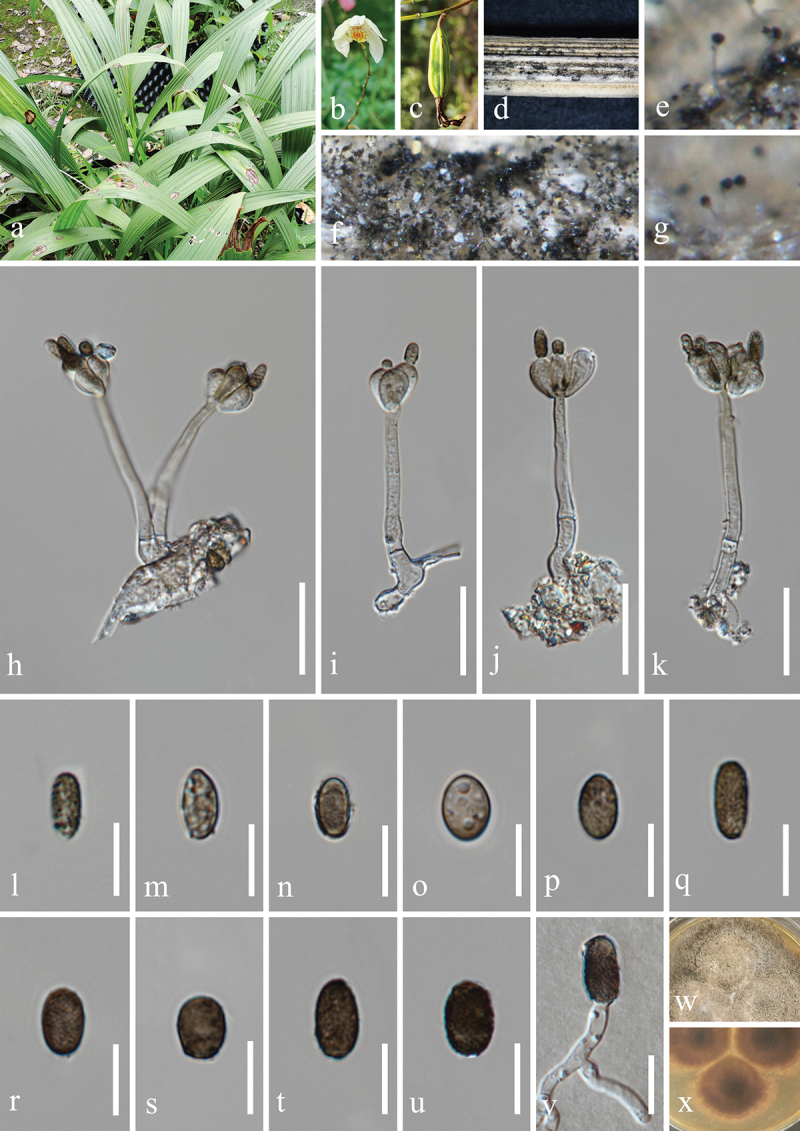
*Stachybotrys chartarum* (HUEST 23.0181, new host record). (a−c) Host *Bletilla striata* (a), flower (b) and fruit (c). (d) Stem of *Bletilla striata*. (e−g) Sporulation on the host substrate. (h−k) Conidiophores, conidiogenous cells, and conidia. (l−u) Conidia. (v) Germinated conidium. (w, x) Colonies on PDA, above (w) and below (x). Scale bars: h−k = 20 µm, l−v = 10 µm.

= *Stachybotrys atra* Corda, Icones Fungorum 1: 21 (1837).

= *Synsporium biguttatum* Preuss, Herbarium Vivum Mycologicum No. 1285 (1849).

= *Memnonium sphaerospermum* Fuckel, *Symbolae Mycologicae*: 358 (1870).

More synonyms see Wang et al. (2015) and Lombard et al. (2016).

**Description**: *Saprobic* on dead stems and petioles of *Bletilla striata* (Orchidaceae). **Sexual morph**: Undetermined. **Asexual morph**: Hyphomycetous. *Colonies* on the substrate surface are black and hairy. *Mycelium* immersed, partly superficial. *Conidiophores* macronematous, mononematous, single or in groups, erect, straight to flexuous, smooth, thin-walled, unbranched or branched, 1–2(−3)-septate, hyaline at the base becoming subhyaline to pale olivaceous brown at the apex, 42–77 µm ($\overset{ˉ}{x}$ = 56 µm, *n* = 30) long, 4–5 µm ($\overset{ˉ}{x}$ = 4 µm, *n* = 30) wide at the base, tapering to 2–3 µm ($\overset{ˉ}{x}$ = 3 µm, *n* = 30) wide at the narrowest point near the apex, bearing 3–6 conidiogenous cells at its apex. *Conidiogenous cells* phialidic, clavate to subclavate, with conspicuous collarettes, discrete or in discrete, terminal, smooth, clustered at the apex of conidiophores, subhyaline to olivaceous brown, 7–11 µm ($\overset{ˉ}{x}$ = 9 µm, *n* = 50) long, 4–7 µm ($\overset{ˉ}{x}$ = 5 μm, *n* = 50) wide at the widest part. *Conidia* aggregated in black heads, acrogenous, ellipsoidal or long ellipsoidal to subcylindrical, unsmooth, reticulate texture and rarely guttules, aseptate, rounded at both ends, olivaceous to light brown when young, brown to dark brown when mature, 7–11 × 4–7 µm ($\overset{ˉ}{x}$ = 9 × 5.5 μm, *n* = 50).

**Culture characteristics**: Conidia germinated on PDA within 24 h, and germ tubes produced from basal cells. Colonies growing on PDA reached 25–27 mm in diameter after two weeks at 25 °C in the dark. Colonies from above, buff to pale luteous, with conidiophores forming on the surface of the medium, carrying slimy mouse grey to black conidial masses. In reverse, becoming pale luteous to light salmon at the margin, brown to dark brown in the middle, the colour gradually lightens from centre to edge, brown pigmentation on PDA.

**Material examined**: CHINA, Guizhou Province, Guiyang City, Huaxi District, Guizhou University of Traditional Chinese Medicine, 106°38′13′′E, 26°22′14′′N, elevation 1,140 m, on dead stems and petioles of medicinal plant *Bletilla striata* (Orchidaceae), 2 June 2021, H.Z. Du, S269 (HUEST 23.0181), living culture UESTCC 23.0181.

*Notes*: *Stachybotrys chartarum* was introduced by Hughes (1958) and reported in different habitats with widespread distribution (Lombard et al. 2016). Morphologically, our collection (HUEST 23.0181) is similar to *Sta. chartarum* (Corda 1837; Wang et al. 2015; Lombard et al. 2016; Maharachchikumbura et al. 2016). The phylogenetic results (Figure 5) showed that our isolate (UESTCC 23.0181) clustered with *Sta. chartarum* (CBS 182.80, ex-type isolate), and multi-locus comparison between UESTCC 23.0181 and CBS 182.80 showed a high similarity by 99.5% in *cmdA*, 99.5% in *rpb2*, 99.7% in *tef1-α*, 99.3% in *tub2*, and 100% in ITS sequence. Therefore, we identify our collection as *Sta. chartarum* and this is a new host record from medicinal plant *Bletilla striata* in China.

***Striatibotrys*** L. Lombard & Crous, Persoonia 36: 224 (2016)

*Notes*: Lombard et al. (2016) introduced the genus *Striatibotrys* (sexual and asexual) within Stachybotryaceae, with *Str. eucylindrospora* (D.W. Li) L. Lombard & Crous as the type species. Asexual morph of *Striatibotrys* is significantly distinguished from *Sirastachys* by the mononematous conidiophores (Lombard et al. 2016), while its sexual morph of *Striatibotrys* has solitary to gregarious, scattered ascomata with protruding papilla, unitunicate asci with 8-biseriate ascospores and ellipsoidal to fusiform, 1-septate ascospores (Crous et al. 2013; Lechat et al. 2017, 2021). Currently, nine species of *Striatibotrys* were accepted in the Species Fungorum (https://www.speciesfungorum.org/Names/Names.asp?strGenus=*Striatibotrys*; accessed on 9 December 2024).

**Figure 22.**
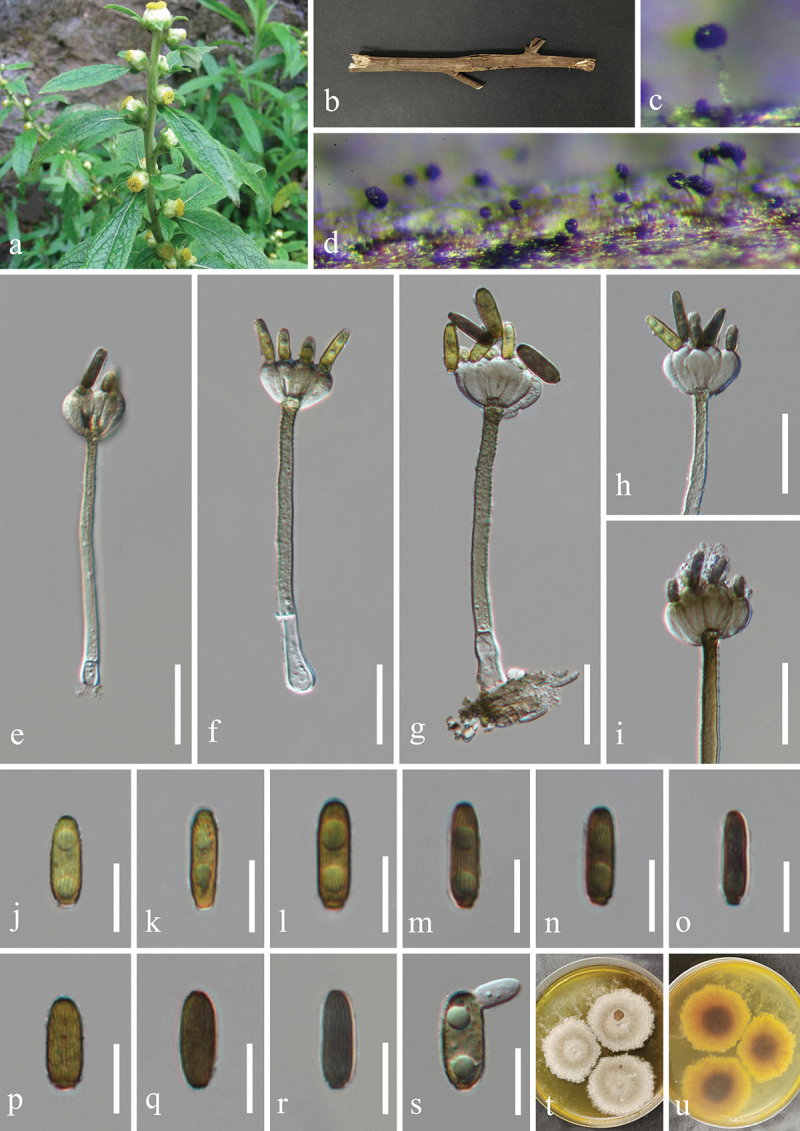
*Striatibotrys biguttulatispora* (HKAS 131306, holotype). (a) Host *Carpesium abrotanoides*. (b) Stem of *Carpesium abrotanoides*. (c, d) Sporulation on the host substrate. (e−g) Conidiophores and conidia. (h, i) Conidiogenous cells and conidia. (j–r) Conidia. (s) Germinated conidium. (t, u) Colonies on PDA, above (t) and below (u). Scale bars: e−i = 20 µm, j−s = 10 µm.

***Striatibotrys biguttulatispora*** H.Z. Du & Jian K. Liu, sp. nov. Figure 22

MycoBank number: MB 854199.

Etymology: The epithet “*biguttulatispora*” refers to the distinctly biguttulate of conidia.

**Description**: *Saprobic* on dead stems of *Carpesium abrotanoides* (Asteraceae). **Sexual morph**: Undetermined. **Asexual morph**: Hyphomycetous. *Colonies* on natural substrate effuse, glistening, black, single or clustered, globose to subglobose nucleus at the apex. *Mycelium* immersed, partly superficial. *Conidiophores* macronematous, mononematous, single or in groups, erect, straight to slightly flexuous, thin-walled, unbranched, hyaline to subhyaline at the base, smooth, becoming subhyaline to pale olivaceous brown at the apex, verrucose, 1–2-septate, 66–106 µm ($\overset{ˉ}{x}$ = 83 µm, *n* = 30) long, 4–6 µm ($\overset{ˉ}{x}$ = 5 µm, *n* = 30) wide at the base, tapering to 3–5 µm ($\overset{ˉ}{x}$ = 4 µm, *n* = 30) wide at the narrowest point near the apex, bearing 4–6 conidiogenous cells. *Conidiogenous cells* phialidic, clavate, with conspicuous collarettes, discrete, terminal, smooth, clustered at the apex of conidiophores, subhyaline to olivaceous brown, 9–14 µm ($\overset{ˉ}{x}$ = 12 µm, *n* = 50) long, 5–7 µm ($\overset{ˉ}{x}$ = 6 μm, *n* = 50) wide at the widest part. *Conidia* aggregated in black and glistening heads, subcylindrical to cylindrical, acrogenous, aseptate, smooth, longitudinally striate, biguttulate, with rounded apical hilum and slightly parallel base, bright yellow to olivaceous brown when young, brown to dark brown when mature, 12–14 × 3.5–6 µm ($\overset{ˉ}{x}$ = 13 × 4.5 μm, *n* = 50).

**Culture characteristics**: Conidia germinated on PDA within 24 h, and germ tubes produced from basal cells. Colonies growing on PDA reached 37–42 mm in diameter after one month at 25 °C in the dark, consisting of mostly immersed mycelium producing luteous exudates diffusing into the medium and the entire PDA is yellowish-brown. Colonies from above, white in the whole colony, circular with filamentous edge, irregular, flat, light brown ring in the middle. In reverse, brown to yellowish brown in the centre, becoming pale luteous to light brown at the margin, bright yellow pigmentation on PDA.

**Material examined**: CHINA, Sichuan Province, Chengdu City, Dujiangyan City, Qingcheng Mountain scenic spot, 103°28′54′′E, 30°56′24′′N, elevation 1,189 m, on dead stems of medicinal plant *Carpesium abrotanoides* (Asteraceae). 27 March 2021, H.Z. Du & N. Wu, S183 (HKAS 131306, holotype), ex-type living culture CGMCC 3.25619; *ibid*., Guizhou Province, Guiyang City, Nanming District, Guiyang Medicinal Botanical Garden, 106°41′48′′E, 26°32′18′′N, elevation 1,127 m, on dead twigs of medicinal plant *Houttuynia cordata* (Saururaceae), 8 May 2021, H.Z. Du, S253 (HKAS 131305, paratype), ex-paratype living culture UESTCC 23.0177; *ibid*., 3 December 2020, H.Z. Du, S34 (HUEST 23.0175, paratype), ex-paratype living culture UESTCC 23.0175; *ibid*., on dead twigs of medicinal plant *Dipsacus asper* (Dipsacaceae), 12 October 2021, H.Z. Du & N. Wu, S402 (HUEST 23.0176, paratype), ex-paratype living culture UESTCC 23.0176; *ibid*., on dead leaves of medicinal plant *Iris wattii* (Iridaceae), 11 October 2021, H.Z. Du, S405 (HUEST 23.0178, paratype), ex-paratype living culture CGMCC 3.25618.

*Notes*: *Striatibotrys biguttulatispora* shares similar morphology with *Str. eucylindrospora*, *Str. neoeucylindrosporus* N.P. Schultes, Marra, R.F. Castañeda & D.W. Li, *Str. Rhabdospora*, and *Str. yuccae* L. Lombard & Crous in having macronematous, mononematous conidiophores with bearing 3–6 conidiogenous cells at the apex, long ellipsoidal to subcylindrical conidia. However, *Str. biguttulatispora* differs from them in having longitudinally striate conidia with two guttules (Li 2007; Lombard et al. 2016; Schultes et al. 2021). The conidiophores of *Str. biguttulatispora* (66–106 × 4–6 µm) are much smaller than *Str. eucylindrospora* (up to 200 × 3–5 µm) (Li 2007) and larger than *Str. rhabdospora* (50–70 × 4–6 µm) and *Str. yuccae* (40−70 × 3–6 µm) (Lombard et al. 2016). Moreover, the conidia of *Str. biguttulatispora* (13–15 × 4–5 µm) are larger than *Str. neoeucylindrosporus* (10–12 × 3–4 µm) (Schultes et al. 2021). Based on phylogenetic analyses, five isolates (CGMCC 3.25619, CGMCC 3.25618, UESTCC 23.0175, UESTCC 23.0176, and UESTCC 23.0177) can be recognised as the same species with high bootstrap support (100% MLBS/1.00 BIPP) (Figure 5). In addition, comparisons of the nucleotide base pair similarity between the ex-type isolates of *Str. eucylindrospora*, *Str. biguttulatispora*, *Str. neoeucylindrosporus*, *Str. rhabdosporus*, and *Str. yuccae* are shown in Table 4. Therefore, *Str. biguttulatispora* is introduced as a new species based on morphological and phylogenetic evidence, and associated with medicinal plants of *Carpesium abrotanoides*, *Dipsacus asper*, *Houttuynia cordata*, and *Iris wattii* in China.

**Table 4.** Comparisons of the nucleotide base pair similarity between the ex-type isolates of *Striatibotrys biguttulatispora* (CGMCC 3.25619) and adjoining sister clade.

Species	Isolates number	*cmdA* (%)	*rpb2* (%)	*tef1-α* (%)	*tub2* (%)
*Str. eucylindrosporus*	CBS 203.61	96.8 (601/621 bp)	98.9 (745/753 bp)	94.9 (428/451 bp)	97.3 (293/301 bp)
*Str. neoeucylindrosporus*	UAMH 7211	97.9 (608/621 bp)	98.6 (931/944 bp)	96.6 (420/435 bp)	98.7 (296/300 bp)
*Str. rhabdosporus*	CBS 528.80	96.9 (603/622 bp)	98.8 (713/722 bp)	96.0 (434/452 bp)	98.3 (296/301 bp)
*Str. yuccae*	CBS 390.68	93.6 (584/624 bp)	97.6 (705/722 bp)	92.9 (418/450 bp)	94.7 (285/301 bp)

***Striatibotrys rhabdospora*** L. Lombard & Crous, Persoonia 36: 226 (2016) Figure 23

**Figure 23.**
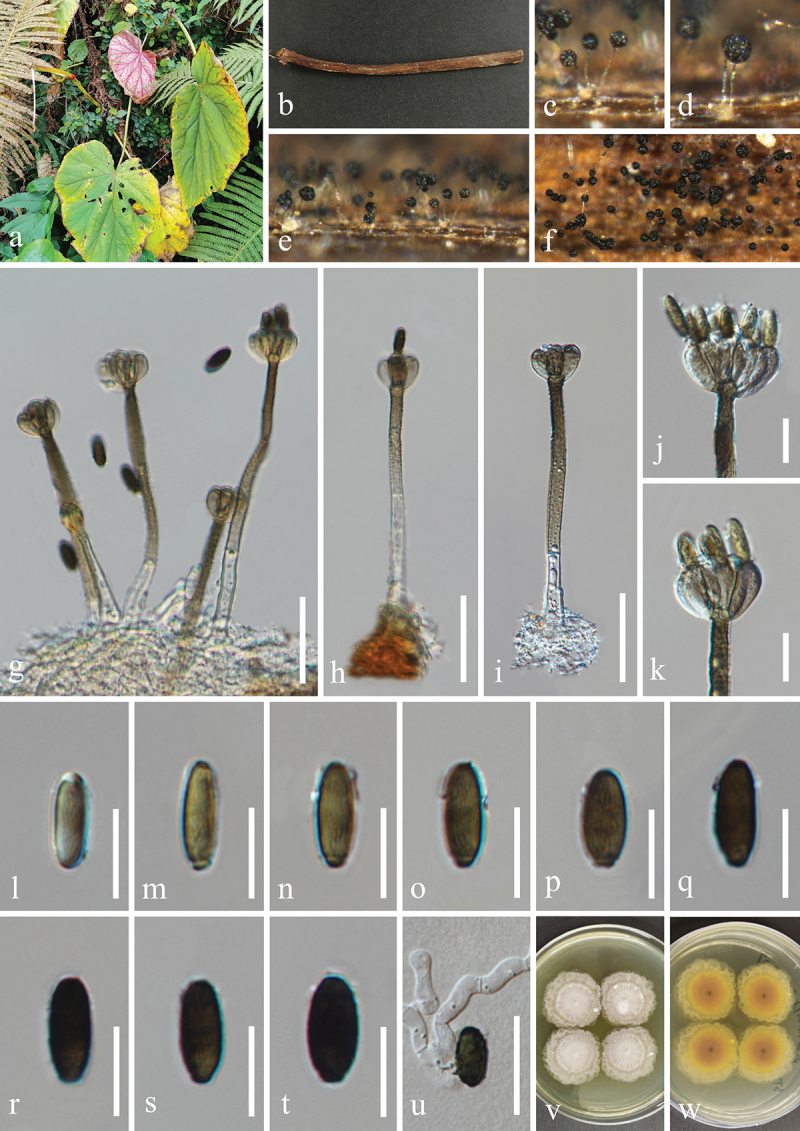
*Striatibotrys rhabdospora* (HKAS 131307, new host and geographical records). (a) Host *Begonia grandis*. (b) Petiole of *Begonia grandis*. (c−f) Sporulation on the host substrate. (g−i) Conidiophores and conidia. (j, k) Conidiogenous cells and conidia. (l−t) Conidia. (u) Germinated conidium. (v, w) Colonies on PDA, above (v) and below (w). Scale bars: g−i = 30 µm, j−t = 10 µm, u = 20 µm.

**Description**: *Saprobic* on dead petioles of *Begonia grandis* (Begoniaceae). **Sexual morph**: Undetermined. **Asexual morph**: Hyphomycetous. *Colonies* on natural substrate effuse, glistening, black, single or clustered, globose nucleus at the apex. *Mycelium* immersed, partly superficial. *Conidiophores* macronematous, mononematous, single or in groups, erect, straight to slightly flexuous, thin-walled, unbranched, 1–2-septate, hyaline at the base, smooth, becoming subhyaline to pale olivaceous brown at the apex, verrucose, 78–111 µm ($\overset{ˉ}{x}$ = 94 µm, *n* = 30) long, 6–7 µm ($\overset{ˉ}{x}$ = 6.5 µm, *n* = 30) wide at the base, tapering to 3–5 µm ($\overset{ˉ}{x}$ = 4 µm, *n* = 30) at the narrowest point near the apex, bearing 3–6 conidiogenous cells at its apex. *Conidiogenous cells* phialidic, clavate, with conspicuous collarettes, not discrete, terminal, smooth, clustered at the apex of conidiophores, olivaceous brown to brown, 9–15 µm ($\overset{ˉ}{x}$ = 11 µm, *n* = 50) long, 4–7 µm ($\overset{ˉ}{x}$ = 5 μm, *n* = 50) wide at the widest part. *Conidia* aggregated in black and glistening heads, acrogenous, aseptate, long ellipsoidal to subcylindrical, smooth, longitudinally striate, with rounded apical hilum and slightly parallel base, olivaceous brown when young, dark brown to black when mature, 10–12.5 × 4–5 µm ($\overset{ˉ}{x}$ = 11.5 × 4.5 μm, *n* = 50).

**Culture characteristics**: Conidia germinated on PDA within 24 h, and germ tubes produced from basal cells. Colonies growing on PDA reached 25–27 mm in diameter after two weeks at 25 °C in the dark, consisting of mostly immersed mycelium producing luteous exudates diffusing into the medium and the entire PDA is light yellow. Colonies from above, white in the whole colony, circular with irregular edge, flat, sparse white mycelium on the outer ring. In reverse, dark brown at the central point, brown in the middle, becoming pale luteous to light brown at the margin, light yellow pigmentation on PDA.

**Material examined**: CHINA, Yunnan Province, Kunming City, Panlong District, Kunming Botanical Garden, 102°44′24′′E, 25°8′27′′N, elevation 1,922 m, on dead petioles of medicinal plant *Begonia grandis* (Begoniaceae), 11 November 2022, H.Z. Du, S744 (HKAS 131307), living culture CGMCC 3.25620.

*Notes*: *Striatibotrys rhabdospora* was introduced by Lombard et al. (2016). Multi-locus phylogeny indicated that our isolate (CGMCC 3.25620) clustered together with the ex-type isolate (CBS 528.80) of *Str. rhabdospora* by strong support (100% MLBS/1.00 BIPP) (Figure 5). Morphological characters of our collection are very similar to the holotype (CBS H-18492) of *Str. rhabdospora* (Lombard et al. 2016). Thus, we identify our collection as *Str. rhabdospora*, and it is the first time reported from medicinal plant of *Begonia grandis* in China.

***Virgatospora*** Finley, Mycologia 59: 538 (1967)

*Notes*: The monotypic asexual genus *Virgatospora* was established by Finley (1967) with *V. echinofibrosa* as the type species. Rossman (1983) transferred *V. echinofibrosa* to the sexual genus *Nectria* (Fr.) Fr. (as *N. spirostriata* Rossman). Later, Rossman et al. (1999) treated *N. spirostriata* as synonymy of *Peethambara spirostriata* (Rossman) Rossman, and *Peethambara* Subram. & Bhat was synonymised under *Didymostilbe* Henn., therefore, a new combination for *V. echinofibrosa* as *D. echinofibrosa* (Finley) Rossman was provided by Rossman et al. (1999) based on similar characteristics in morphology. Subsequently, Lombard et al. (2016) revalidated *Virgatospora* in Stachybotryaceae and it was distant to *Didymostilbe* clade based on multi-locus sequence analyses. *Virgatospora* is characterised by dark brown to olivaceous grey, synnematal conidiomata, and 3-septate, fusiform, olivaceous grey, coarsely striate conidia with papillate tip and truncate base (Finley 1967; Lombard et al. 2016).

***Virgatospora echinofibrosa*** Finley, Mycologia 59: 538 (1967) Figure 24

**Figure 24.**
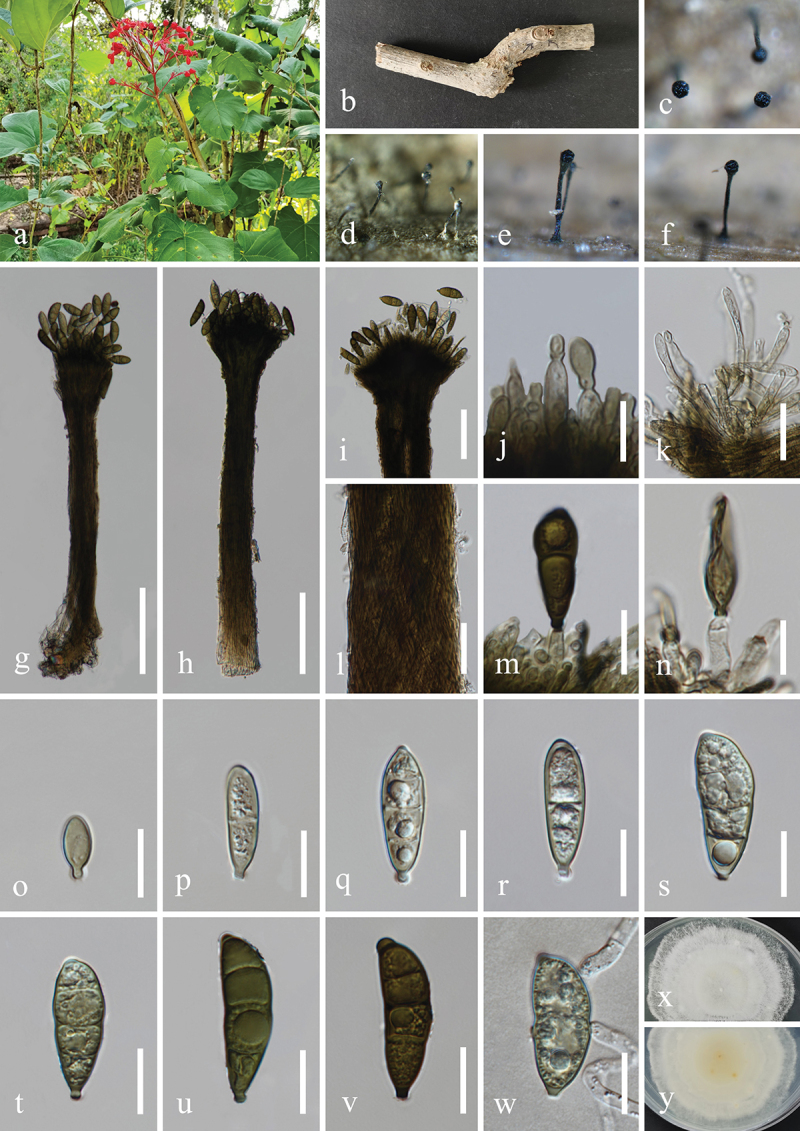
*Virgatospora echinofibrosa* (HUEST 23.0185, new host and geographical records). (a) Host *Clerodendrum japonicum*. (b) Twig of *Clerodendrum japonicum*. (c−f) Sporulation on the host substrate. (g−i) Conidiophores and conidia. (l) Omamentation of conidiophore. (j, k, m, n) Conidiogenous cells and conidia. (o−v) Conidia. (w) Germinated conidium. (x, y) Colony on PDA, above (x) and below (y). Scale bars: g, h = 100 µm, i = 60 µm, j, *n* = 10 µm, k, l = 20 µm, m, o−w = 15 µm.

= *Didymostilbe echinofibrosa* (Finley) Rossman, Studies in Mycology 42: 56 (1999).

= *Nectria spirostriata* Rossman, Mycological Papers 150: 61 (1983).

= *Peethambara spirostriata* (Rossman) Rossman, Studies in Mycology 42: 56 (1999).

**Description**: *Saprobic* on dead twigs of *Clerodendrum japonicum* (Lamiaceae). **Sexual morph**: Undetermined. **Asexual morph**: Hyphomycetous. *Colonies* on natural substrate effuse, single, erect, black. *Mycelium* immersed, partly superficial. *Conidiophores* erect, straight to slightly flexuous, thick-walled, septate, unbranched or branched, smooth below, brown or dark brown to black, cylindrical below the neck, distinctly enlarged towards the flared apex, bearing conidiogenous cells, (340–)600–830 µm ($\overset{ˉ}{x}$ = 692 µm, *n* = 30) long, 34–74 µm ($\overset{ˉ}{x}$ = 52 µm, *n* = 30) wide at the base, 37–78 µm ($\overset{ˉ}{x}$ = 56 µm, *n* = 30) wide in the middle, 40–86 µm ($\overset{ˉ}{x}$ = 62 µm, *n* = 30) wide near the apex, (71–)130–180(−210) µm ($\overset{ˉ}{x}$ = 160 µm, *n* = 30) wide in the flared apex. *Conidiogenous cells* elongate, subcylindrical, straight or slightly curved, smooth, monophialidic, discrete, mostly terminal, sometimes subterminal, 18–25 µm ($\overset{ˉ}{x}$ = 22 µm, *n* = 50) long, 3–4 µm ($\overset{ˉ}{x}$ = 2.5 µm, *n* = 50) wide in the middle, up to 1.5–2 µm ($\overset{ˉ}{x}$ = 2 µm, *n* = 50) wide at the tip, with a distinct collarette 2–3 µm ($\overset{ˉ}{x}$ = 2.5 µm, *n* = 50) wide at the apex. *Conidia* aggregated in black heads, solitary, ellipsoidal to fusiform or timoniform, papillate at both ends, curved, 3-septate (phragmosporous), smooth-walled, with distinct transverse ridges on the surface, pale olive gray or pale olive green to brown when young, dark olive green to dark brown when mature, (10–)25–45 µm ($\overset{ˉ}{x}$ = 36 µm, *n* = 50) long, (5–) 7–15 µm ($\overset{ˉ}{x}$ = 13 µm, *n* = 50) wide in the middle.

**Culture characteristics**: Conidia germinated on PDA within 24 h, and germ tubes produced from basal cells. Colonies growing on PDA reached 64–65 mm in diameter after three weeks at 25 °C in the dark. Colonies from above, white in the whole colony, surface smooth, circular with entire edge, sparse white mycelium on the outer ring. In reverse, white mycelium at the margin, white to light yellow in the centre.

**Material examined**: CHINA, Yunnan Province, Xishuangbanna Dai Autonomous Prefecture, Xishuangbanna Tropical Botanical Garden Chinese Academy of Sciences. 101°15′16′′E, 21°55′51′′N, elevation 505 m, on dead twigs of medicinal plant *Clerodendru japonicum* (Lamiaceae), 9 November 2022, H.Z. Du, S619 (HUEST 23.0185), living culture UESTCC 23.0185.

*Notes*: In this study, our collection (HUEST 23.0185) is morphologically similar to the holotype of *Virgatospora echinofibrosa* by synnematous conidiomata and ellipsoidal to timoniform, papillate, curved, 3-septate conidia (Finley 1967). The phylogenetic analyses (Figure 5) indicated that our isolate (UESTCC 23.0185) grouped with *V. echinofibrosa* (MUCL 39092 and CBS 110115) by absolute bootstrap support (100% MLBS/1.00 BIPP). Therefore, we identify the new collection as *V. echinofibrosa* based on morphology and phylogeny. *Virgatospora echinofibrosa* has been found in Ecuador, Nepal, and Panama (Finley 1967; Lombard et al. 2016), and we report it as a new host and geographical record from medicinal plant *Clerodendrum japonicum* in China.

**Table 5.** Synopsis of the morphological characteristics, distribution, and hosts of *Memnoniella* species.

Species	Morphology of conidia					Distribution and hosts	References
	Shape	Size (µm)	Colour	Septate	Omamentation		
*Memnoniella alishanensis*	Ellipsoidal, reniform or comma	10–12 × 5–6	Olivaceous brown to dark brown	Aseptate	Verrucose, with 1–2 guttules, some mesh texture	China, dead leaves of *Macaranga tanarius* (Euphorbiaceae) and *Morus australis* (Moraceae); China, dead stems of *Dregea volubilis* (Apocynaceae), *Justicia brandegeeana* (Acanthaceae) and *Disporopsis longifolia* (Asparagaceae); China, dead branches of *Baliospermum solanifolium* (Euphorbiaceae) and *Alangium chinense* (Cornaceae)	Tennakoon et al. (2021); In this study
*M. brunneoconidiophora*	Ellipsoidal	7.5–8.5 × 3.5–4.5	Olivaceous brown to dark brown	Aseptate	Verrucose	Venezuela, on decayed leaf	Lombard et al. (2016)
*M. celtidis*	Ovoid to ellipsoidal	9–11 × 4.5–6	Olive green to light brown	Aseptate	2–3 guttules	China, dead leaves of *Celtis formosana* (Cannabaceae)	Tennakoon et al. (2021)
*M. chromolaenae*	Globose to subglobose (chains)	3–4.5 × 3.5–4.8	Olivaceous grey to black	Aseptate	Smooth	Thailand, dead stems of *Chromolaena odorata*	Mapook et al. (2020)
*M. cnidiicola*	Ellipsoidal	9–15 × 5–8	Olivaceous or olivaceous brown to dark brown	Aseptate	Verrucose, mostly 2 guttules	China, dead stems of *Cnidium monnieri* (Apiaceae) and *Gynostemma pentaphyllum* (Cucurbitaceae)	In this study
*M. dichroa*	Ovoid, slightly curved into bean shape	8.5–11.5 × 4.5–6	Olivaceous	Aseptate	Coarsely roughened	England, herbaceous stems; Netherlands, leaf litter of *Ilex aquifolium*	Grove (1886); Wang et al. (2015); Lombard et al. (2016)
*M. echinata*	Globose (chains)	3.2–6.5 × 3.5–5	Olivaceous brown to dark brown	Aseptate	Verrucose	China, dead leaves of *Macaranga tanarius* (Euphorbiaceae); Canada, air; Indonesia, endotracheal tubes in a hospital and filter paper; Japan, contaminated sake lees, isotype of *Spinomyces japonica*; Solomon Islands, tent canvas; Netherlands, *Pulvinula constellation*; America	Lombard et al. (2016); Tennakoon et al. (2021)
*M. ellipsoidea*	Ellipsoidal	8–13 × 4–8	Olivaceous brown to dark brown	Aseptate	Verrucose, gluttulate	Brazil, *Bromelia* sp.; Nepal, dead twig; China, dead stems of *Reynoutria japonica* (Polygonaceae)	Lombard et al. (2016); In this study
*M. guttulatispora*	Ellipsoidal	10–12 × 5–7	Olivaceous, brown to dark brown or black	Aseptate	Verrucose, 2 guttules, rarely 1 guttule	China, on dead petioles of *Alsophila spinulosa* (Cyatheaceae)	In this study
*M. humicola*	Ellipsoidal to reniform	5.5–6.5 × 2–3	Olivaceous brown to dark brown	Aseptate	Verrucose	Suriname, soil under *Elaeis guineensis*	Lombard et al. (2016)
*M. levispora*	Globose to subglobose (chains)	2.5–4.3 × 1.5–3.6	Gray, dark brown to black	Aseptate	Smooth	China, dead leaf of *Musa* sp.	Samarakoon et al. (2021)
*M. longistipitata*	Dimorphic conidia; Sphaerical or subsphaerical (catenate); Oblong or ovoid (non-catenate)	5.8–8.5×6.3–8.3 (catenate); 10.5–12 × 4.8–5.7 (non-catenate)	Dark olivaceous or black (catenate); Dark olivaceous (non-catenate)	Aseptate	Warty (catenate); Smooth-walled to slightly rough (non-catenate)	Japan, solo sylvarum isolatus; Malawi, dead wood	Li et al. 9^2003^); Wang et al. (2015); Lombard et al. (2016)
*M. mori*	Oblong	10–13 × 5–8	Olivaceous brown to dark brown	Aseptate	Verrucose, guttulate	China, dead leaves of *Morus australis* (Moraceae)	Tennakoon et al. (2021)
*M. oblongispora*	Dimorphic conidia; Globose or oblong (chains)	3–5 × 2.5–4.5 (globose); 8–14 × 4.5–7 (oblong)	Olivaceous, brown to dark brown or reddish brown (globose); Hyaline, olive green to dark brown or black (oblong)	Aseptate	Verrucose (globose); Verrucose, with 2 guttules	Thailand, decaying leaf of *Quercus* sp.; Thailand, dead stems of *Clematis subumbellata*	Lin et al. (2016); Phukhamsakda et al. (2020)
*M. oenanthes*	Ellipsoidal to reniform	9–12 × 4.5–8	Smoke grey to black	Aseptate	Verrucose or smooth	India, old stem of *Euphorbia tirukalli*	Ellis (1971); Wang et al. (2015); Lombard et al. (2016)
*M. pseudodichroa*	Ellipsoidal to short-cylindrical	14–15.5 × 6–7	Dark olive to dark brown	Aseptate	2 guttules, sometimes 1 guttule, rugose	China, dead petiole of *Angiopteris lygodiifolia* (Marattiaceae)	Yeh et al. (2023)
*M. pseudonilagirica* (= *M. nilagirica*)	Globose to subglobose	18–22 × 17–21	Olivaceous brown to dark brown	Aseptate	Verrucose	Nepal, dead leaf of *Ceiba pentandra*; Thailand, decaying wood	Lin et al. (2016); Lombard et al. (2016); In this study
*M. putrefolia*	Ellipsoidal to rarely reniform	9–11 × 4.5–5.5	Olivaceous brown to dark brown	Aseptate	Verrucose	Brazil, decayed leaf; Puerto Rico, decayed leaf of Melastomataceae	Lombard et al. (2016)
*M. reniformis*	Ovoid to reniform or comma	9.5–12 × 5–6	Olivaceous brown to dark brown	Aseptate	Verrucose, 2 guttules, rarely 3–4 unequal guttules	China, dead petioles of *Dryopteris* sp. (Dryopteridaceae)	In this study
*M. reynoutriae*	Ellipsoidal	12–17 × 6–10	Subhyaline to olivaceous, brown to dark brown	Aseptate	Verrucose, 2 guttules, rarely 1 guttule	China, dead stems of *Reynoutria japonica* (Polygonaceae)	In this study
*M. sinensis*	Ellipsoidal or sometimes ovoid	10.1–13.4 × 5.12–8.1	Olivaceous brown to dark brown	Aseptate	Verrucose, guttulate	China	Zheng et al. (2019)
*M. verrucosispora*	Ellipsoidal	11–15.5 × 6.5–9	Olive grey or olivaceous, brown to dark brown or black	Aseptate	Verrucose, with 1–2 guttules	China, dead stems of *Fibraurea recisa* (Menispermaceae)	In this study

**Table 6.** Synopsis of the morphological characteristics, distribution, and hosts of *Sirastachys* species.

Species	Morphology of Conidia					Distribution and hosts	References
	Shape	Size (µm)	Colour	Septate	Omamentation		
*Sirastachys aspidistrae*	Obovoid to ellipsoidal	4–6 × 3–4	Olivaceous, brown to dark brown	Aseptate	2 guttules	China, dead petioles of *Aspidistra elatior* (Asparagaceae)	In this study
*Sir. castanedae*	Obovoid	4.5–5.5 × 2–3	Darkly olivaceous to dark brown	Aseptate	Verrucose, rarely with 1–2 guttules	China, dead stems *Lonicera guillonii* var. *macranthoides* (Caprifoliaceae); China, dead leaves of *Ficus septica* (Moraceae) and *Morus* sp. (Moraceae); Canada, soil under *Thuja occidentalis*; Iran, *Morus* sp.; Spain, decaying leaf	Lombard et al. (2016); Tennakoon et al. (2021); In this study
*Sir. cylindrospora*	Cylindrical	8.5–9.5 × 2–3	Hyaline	Aseptate	Smooth	Brazil, decaying leaves	Lombard et al. (2016)
*Sir. cyperacearum*	Ellipsoidal	6–7 × 2.5–3	Dark brown	Aseptate	Verrucose, guttulate	Australia, leaves of Cyperaceae	Crous et al. (2018)
*Sir. ellipsoidispora*	Obovoid to ellipsoidal	5–8 × 3–6	Olivaceous to brown, dark brown to black	Aseptate	Verrucose, mostly 1 guttulate, rarely 3 guttules	China, dead stems of *Disporum longistylum* (Liliaceae)	In this study
*Sir. longispora*	Cylindrical	8.8–12 × 2–2.4	Pale olivaceous	Aseptate	Smooth	–	Matsushima (1975); Wang et al. (2015); Lombard et al. (2016)
*Sir. pandanicola*	Obovoid to ellipsoidal	3.3–5 × 2.2–3.6	Darkly olivaceous to dark brown	Aseptate	Verrucose, mostly 2 guttules	China, dead leaves of *Celtis formosana* (Cannabaceae); Singapore, decaying leaf of *Pandanus* sp. (Pandanaceae)	Lombard et al. (2016); Tennakoon et al. (2021)
*Sir. phaeospora*	Obovoid to ellipsoidal	4–5 × 2–3	Darkly olivaceous to dark brown	Aseptate	Verrucose	Brazil, decaying leaf of unknown host; Cuba, decaying leaves in rain forest; South Africa, *Cycas* sp.; Netherlands, soil	Lombard et al. (2016)
*Sir. phangngaensis*	Obovoid to ellipsoidal	3.5–4 × 1.5–2	Reddish-brown to green-brown	Aseptate	Guttulate, rough-walled	Thailand, dead leaf of *Pandanus* sp.	Tibpromma et al. (2018)
*Sir. phyllophila*	Ellipsoidal	4–5 × 2–3	Darkly olivaceous	Aseptate	Verrucose	Spain, decaying leaves	Lombard et al. (2016)
*Sir. pseudolongispora*	Cylindrical	9.5–10.5 × 2	Hyaline to olivaceous brown	Aseptate	Smooth	Cuba, decaying leaves in rain forest; La Habana, leaf litter	Lombard et al. (2016)

**Table 7.** Synopsis of the morphological characteristics, distribution, and hosts of *Striatibotrys* species.

Species	Morphology of ascospores and conidia					Distribution and hosts	References
	Shape	Size (µm)	Colour	Sepatate	Omamentation		
*Striatibotrys alpina*	Ellipsoidal to fusiform (sexual)	19–24 × 5.5–6.5	Hyaline, yellow to pale orange	1-septate	Finely spinulose	France, dead stem of *Adenostyles alpina*	Lechat et al. (2021)
*Str. asturiensis*	Ellipsoidal to fusiform (sexual)	19–24 × 6.5–8.5	Orange	1-septate	Conspicuously spinulose, surrounded by a sheath	Spain, dead stems of *Veratrum album*	Lechat et al. (2017)
*Str. atypica*	Ellipsoidal to subcylindrical	7–9 × 3–4	Olivaceous brown to dark brown	Aseptate	Smooth, rarely longitudinally striate	France, *Iris* sp.	Lombard et al. (2016)
*Str. biguttulatispora*	Subcylindrical to cylindrical	12–15 × 4–6	Bright yellow to olivaceous brown, brown to dark brown	Aseptate	Smooth, 2 guttules, longitudinally striate	China, dead stems of *Houttuynia cordata* (Saururaceae), *Carpesium abrotanoides* (Asteraceae) and *Dipsacus asper* (Dipsacaceae); China, dead leaves of *Iris wattii* (Iridaceae)	In this study
*Str. eucylindrospora*	Cylindrical or cylindrical ellipsoid	12.8–16 × 3.4–5.5	Gray to dark olive grey	Aseptate	Smooth, longitudinal striations, bi-guttulate	Canada, soil under *Thuja occidentalis*; Turkey, Izmir; America, plant debris	Li (2007); Lombard et al. (2016)
*Str. humicola*	Fusiform	7.5–8.5 × 3.5–4.5	Olivaceous brown to dark brown	Aseptate	Smooth, longitudinally striate	America, soil	Lombard et al. (2016)
*Str. neoeucylindrosporus*	Clavate, subcylindrical or cylindrical ellipsoid, or dumbbell- shaped	10.3–12.3 × 3–3.8	Olivaceous grey to dark brown	Aseptate	Smooth, longitudinally striate, bi-guttulate	Canada, *Populus tremuloides* (Salicaceae); America, paper towelling in moist chamber with rotting agaric	Schultes et al. (2021)
*Str. oleronensis*	Ellipsoidal to fusiform (sexual)	16–18.5 × 4.5–5	Orange	1-septate	Spinulose, 1–2 guttules, without sheath	France, dead leaf of *Iris pseudacorus*	Crous et al. (2013); Lombard et al. (2016)
*Str. rhabdospora*	Ellipsoidal to subcylindrical	9.5–10.5 × 3–4	Olivaceous brown to dark brown	Aseptate	Smooth, longitudinally striate	China, dead petioles of *Begonia grandis* (Begoniaceae); Belgium, asbestos cement tile on building roof; Germany, soil under *Triticum* sp.; Spain, plant debris; Switzerland, soil with *Trichophaea woolhopeia*; America, petiole of *Caltha palustris*	Lombard et al. (2016); In this study
*Str. yuccae*	Ellipsoidal to subcylindrical	9–9.5 × 3–4	Olivaceous brown to dark brown	Aseptate	Smooth, longitudinally striate	Netherlands, dead leaf of *Yucca flaccida*	Lombard et al. (2016)

## 4. Discussion

Thirty-nine isolates of Dictyosporiaceae and Melanommataceae (Pleosporales), Stachybotryaceae (Hypocreales) were obtained from medicinal plants in Southwestern China (Yunnan, Guizhou, and Sichuan Provinces), and they were identified as nineteen species based on morphological characteristics and phylogenetic analyses of combined *cmdA*, ITS, LSU, *rpb2*, *tef1-α*, and *tub2* sequence data. This study included the discovery of twelve novel species and the identification of seven known species. All collections were reported from terrestrial habitats and associated with medicinal plants belonging to twenty-one families. Additionally, the fresh samples of microfungi were primarily collected from dead leaves, petioles, stems, twigs, and vines. Besides their medicinal value, some host plants also possess economic significance, such as *Bletilla striata* (Orchidaceae) (Wiart 2012; Wang et al. 2015; He et al. 2016; Gou et al. 2022), edible value, such as *Houttuynia cordata* (Saururaceae) (Seal et al. 2016; Jin et al. 2019; Zhu et al. 2020) and *Disporopsis longifolia* (Asparagaceae) (du Toit et al. 2010; Nguanchoo et al. 2014). Some hosts are cherished and endangered medicinal plants, such as *Alsophila spinulosa* (Cyatheaceae) (Tryon 1970; Fu and Jin 1992), known as living fossils.

In this study, we summarised the main morphological characteristics of conidia, distribution, and hosts of *Memnoniella*, *Sirastachys*, and *Striatibotrys* (Tables 5–7). Based on the conidial morphology, *Memnoniella* can be divided into two categories: globose conidia and non-globose (including ellipsoidal, cylindrical, oblong, ovoid, and reniform) conidia. In our phylogenetic analyses (Figure 5), *Memnoniella* species with globose conidia grouped in Clade B and Clade C. However, *M. longistipitata* and *M. oblongispora* with dimorphic conidial, also produced oblong conidia (Li et al. 2003; Lombard et al. 2016; Phukhamsakda et al. 2020). While most *Memnoniella* species with non-globose conidia clustered in Clade A. *Memnoniella humicola*, with ellipsoidal to reniform conidia, formed a basal lineage (Clade D) in *Memnoniella* group. *Striatibotrys* also can be divided into two categories: cylindrical conidia and ellipsoidal conidia. Species with cylindrical conidia formed a well-supported clade (Clade E), while other species with ellipsoidal conidia clustered in Clade F and Clade G. Two types of conidia (cylindrical and obovoid) are reported in *Sirastachys*. However, there are no obvious patterns like those observed in *Memnoniella* and *Striatibotrys*. Three species, *Sir. cylindrospora* (Clade I), *Sir longispora* (Clade J), and *Sir. pseudolongispora* (Clade H) with cylindrical conidia were nested within other *Striatibotrys* species with obovoid conidia.

Through the nucleotide base pair comparisons of the individual loci defined for *Memnoniella*, the *rpb2*, *tef1-α*, and *tub2* gene regions exhibited significant differences among species, whereas ITS locus failed to identify most of the species. For example, comparisons of the *rpb2*, *tef1-α*, and *tub2* sequences of *Memnoniella alishanensis* (UESTCC 23.0159) and *M. guttulatispora* (UESTCC 23.0164) revealed 97.0% (1,049/1,082 bp), 93.9% (387/412 bp), and 96.6% (286/296 bp) similarity, respectively, but ITS sequence showed 99.4% (500/503 bp) similarity. For *Striatibotrys* species, the *cmdA*, *rpb2*, and *tef1-α* gene regions provided significantly differences with well-supported statistical values, but ITS sequence data did not resolve the phylogenetic status of similar clades. For instance, a comparison of *Str. biguttulatispora* (CGMCC 3.25619) and *Str. rhabdospora* (CBS 528.80) showed that nearly identical ITS sequences (542/543 bp, 99.8%), but the *cmdA*, *rpb2*, and *tef1-α* genes revealed sequence similarities of 96.9% (602/621 bp), 98.9% (713/721 bp), and 95.0% (420/442 bp), respectively. For *Str. biguttulatispora* (CGMCC 3.25619) and *Str. neoeucylindrosporus* (UAMH 7211). Comparisons of the *cmdA*, *rpb2*, and *tef1-α* sequences, showed 97.7% (606/620 bp), 99.0% (938/947 bp), and 95.3% (388/407 bp) similarity, respectively. But ITS locus, revealed high similarity (541/543 bp, 99.6%). These examples highlight the limitations of using ITS sequences to identify *Memnoniella* and *Striatibotrys* species.

Lombard et al. (2016) stated that partial sequences of the *rpb2*, *cmdA*, and *tub2* gene regions provided the best statistical support to resolve *Myrothecium* s.l. species, while *tef1-α* was excluded due to its inclusion of numerous ambiguous regions. The *cmdA* and *rpb2* genes were the best loci to resolve *Stachybotrys* s.l. species. In our study, single-gene analyses of *cmdA*, *rpb2*, *tef1-α*, and *tub2* produced similar topologies without significant conflictions for *Memnoniella*, *Sirastachys*, and *Striatibotrys*, respectively. The *tef1-α* gene region proved to be vital for identifying loci at the generic and species level. Therefore, it is necessary to test single gene analyses to select suitable loci when dealing with Stachybotryaceae, as some genes may be unsuitable for specific groups within this family (Lin et al. 2016; Lombard et al. 2016; Samarakoon et al. 2021).

Jeewon and Hyde (2016) emphasised the importance of phylogenetic assumptions and morphological characters for species delineation. However, for Stachybotryaceae species, due to their high morphological similarities, it is challenging to identify species without molecular evidence. For example, the holotype of *Memnoniella mori* (MFLU 18-2575) is morphologically similar to *M. sinensis* (YMFT 1.05582) (Zheng et al. 2019; Tennakoon et al. 2021), but they formed distinct lineages as different phylogenetic species (Figure 5). Comparing the holotype of *Striatibotrys rhabdospora* (CBS H-18492) and *Str. yuccae* (CBS H-22470), it is difficult to find significant morphological differences between them (Lombard et al. 2016). Additionally, some species, like *Memnoniella* sp. (MUCL 50191), lack morphological descriptions and have not yet been identified. Therefore, taxonomic work based on herbaria and fresh collections is continually needed, and phylogenetic evidence and nucleotide pairwise comparison provide adequate justification for the novelty of species following the recommendation of Jeewon and Hyde (2016).
